# A non-randomized trial to assess the safety, tolerability, and pharmacokinetics of posaconazole oral suspension in immunocompromised children with neutropenia

**DOI:** 10.1371/journal.pone.0212837

**Published:** 2019-03-26

**Authors:** Antonio C. Arrieta, Lillian Sung, John S. Bradley, C. Michel Zwaan, Davis Gates, Hetty Waskin, Patricia Carmelitano, Andreas H. Groll, Thomas Lehrnbecher, Eric Mangin, Amita Joshi, Nicholas A. Kartsonis, Thomas J. Walsh, Amanda Paschke

**Affiliations:** 1 Children’s Hospital of Orange County, 455 S. Main St, Orange County, CA, United States of America; 2 The Hospital for Sick Children, Paediatric Oncologist, Haematology/Oncology, Toronto, Ontario Canada; 3 University of California, Division of Infectious Disease, Department of Pediatrics/Rady Children’s Hospital, San Diego, CA, United States of America; 4 Erasmus MC-Sophia Children’s Hospital, Department of Pediatric Oncology, Rotterdam, Netherlands; 5 Merck & Co., Inc., Merck Research Laboratories, Kenilworth, NJ, United States of America; 6 University Children’s Hospital Münster, Infectious Disease Research Program, Center for Bone Marrow Transplantation and Department of Pediatric Hematology/Oncology, Münster, Germany; 7 Universitäts Klinikum Frankfurt, Goethe-Universität, Frankfurt, Germany; 8 Transplantation-Oncology Infectious Diseases Program, Departments of Medicine, Pediatrics, and Microbiology & Immunology, Weill Cornell Medicine of Cornell University, New York, NY, United States of America; University of Newcastle, AUSTRALIA

## Abstract

**Background:**

Posaconazole (POS) is a potent triazole antifungal agent approved in adults for treatment and prophylaxis of invasive fungal infections (IFIs). The objectives of this study were to evaluate the pharmacokinetics (PK), safety, and tolerability of POS oral suspension in pediatric subjects with neutropenia.

**Methods:**

This was a prospective, multicenter, sequential dose-escalation study. Enrolled subjects were divided into 3 age groups: AG1, 7 to <18 years; AG2, 2 to <7 years; and AG3, 3 months to <2 years. AG1 and AG2 were divided into 3 dosage cohorts: DC1, 12 mg/kg/day divided twice daily (BID); DC2, 18 mg/kg/day BID; and DC3, 18 mg/kg/day divided thrice daily (TID). AG3 was also divided into DC1 and DC2; however, no subjects were enrolled in DC2. Subjects received 7–28 days of POS oral suspension. PK samples were collected at predefined time points. The POS PK target was predefined as ~90% of subjects with C_avg_ (AUC /dosing interval) between 500 and 2500 ng/mL, with an anticipated mean steady state C_avg_ exposure of ~1200 ng/mL.

**Results:**

The percentage of subjects meeting the PK target was <90% across all age groups and dosage cohorts (range: 31% to 80%). The percentage of subjects that achieved the C_avg_ target of 500 to 2500 ng/mL on Day 7 ranged from 31% to 80%, with the lowest proportion in subjects 2 to <7 years receiving 12 mg/kg/day BID (AG2/DC1) and the highest proportion in subjects 7 to <18 years receiving 18 mg/kg/day TID (AG1/DC3). At all three dose levels (12 mg/kg/day BID, 18 mg/kg/day BID and 18 mg/kg/day TID), subjects in AG1 (7 to <18 years old) had higher mean PK exposures at steady state than those in AG2. High variability in exposures was observed in all groups. POS oral suspension was generally well tolerated and most of the reported adverse events were related to the subjects’ underlying diseases.

**Conclusion:**

The POS PK target of 90% of subjects with C_avg_ between 500 and 2500 ng/mL was not achieved in any of the age groups across the different dosage cohorts. New formulations of the molecule with a greater potential to achieve the established PK target are currently under investigation.

**Trial registration:**

ClinicalTrials.gov identifier: NCT01716234

## Introduction

Pediatric subjects who receive dose-intensive chemotherapy for cancer are at an increased risk of invasive fungal infections (IFIs). The pediatric population at high risk for developing IFIs includes, but is not limited to, allogeneic hematopoietic stem cell transplant (HSCT) recipients, patients with acute myeloid leukemia (AML), relapsed acute lymphoblastic leukemia (ALL), or severe aplastic anemia, and patients receiving immunosuppressant or high-dose corticosteroids for severe graft-versus-host disease (GVHD) or other conditions [1–6]. IFIs are responsible for considerable morbidity, mortality, and healthcare utilization and may prevent or delay the delivery of appropriate chemotherapy [7, 8]. Additionally, the incidence of IFIs in the pediatric population appears to have increased over the past few decades, primarily because of the prolonged survival of pediatric patients with primary or secondary immune deficiencies [9–11]. Prophylaxis for IFIs is indicated in high-risk pediatric patients, and recommended agents include fluconazole, liposomal amphotericin, and micafungin; however, more clinical data supporting use of newer antifungal agents in this population are needed, particularly with regard to appropriate pediatric dosing [12].

Posaconazole (POS) is a broad-spectrum triazole antifungal compound which exhibits potent antifungal activity against a variety of yeasts and molds, including strains that are resistant to amphotericin B, fluconazole, voriconazole, or itraconazole. The efficacy of POS in both prophylaxis and treatment has been established in adults using the oral suspension [13, 14]. Two prior prophylaxis studies in adults helped in defining the basic pharmacokinetic (ADME) and safety parameters for the pediatric study (6,14). As the pharmacokinetic (PK), safety, and efficacy data for POS in pediatric subjects are sparse, dosing recommendations for patients <13 years old has not been established. Thus, we conducted the first dedicated clinical trial of POS in the pediatric population. The primary objective was to evaluate the PK of POS oral suspension administered orally at three dosage levels to immunocompromised children aged 3 months to <18 years with neutropenia or expected neutropenia. The PK criteria were based on data from the adult prophylaxis and treatment studies in which doses above 800 mg per day were not found to be beneficial because of the plateau in POS exposure, which may also occur in pediatric subjects. The secondary objectives were to evaluate the safety and tolerability of POS oral suspension at these three dosage levels.

## Materials and methods

### Study design

This study (MK 5592–032, NCT01716234) was a prospective, non-randomized, multicenter, open-label, sequential dose-escalation study to evaluate the PK, safety, and tolerability of POS oral suspension in children aged 3 months to <18 years with neutropenia or expected neutropenia (defined as an absolute neutrophil count [ANC] ≤500/mm^3^). This study was conducted at centers in the US, Germany, Canada, Greece, and the Netherlands. An Institutional Review Board/independent ethics committee for each clinical site approved the study protocol, a full list can be found in the S1 Table. All clinical investigations were conducted according to principles expressed in the Declaration of Helsinki and Good Clinical Practice Guidelines. Patients were enrolled starting 06 Jun 2008 and last subject visit for follow up was 01 Apr 2015. At enrollment, informed consent was obtained from each participant and his/her legal guardian, and assent was obtained where appropriate. The authors confirm that all ongoing and related trials for this drug/intervention are now registered. An initial delay in the trial registration occurred due to the transfer of this study from Schering Plough to Merck and the differences in the trial registration requirements of the two companies.

**Fig 1:**
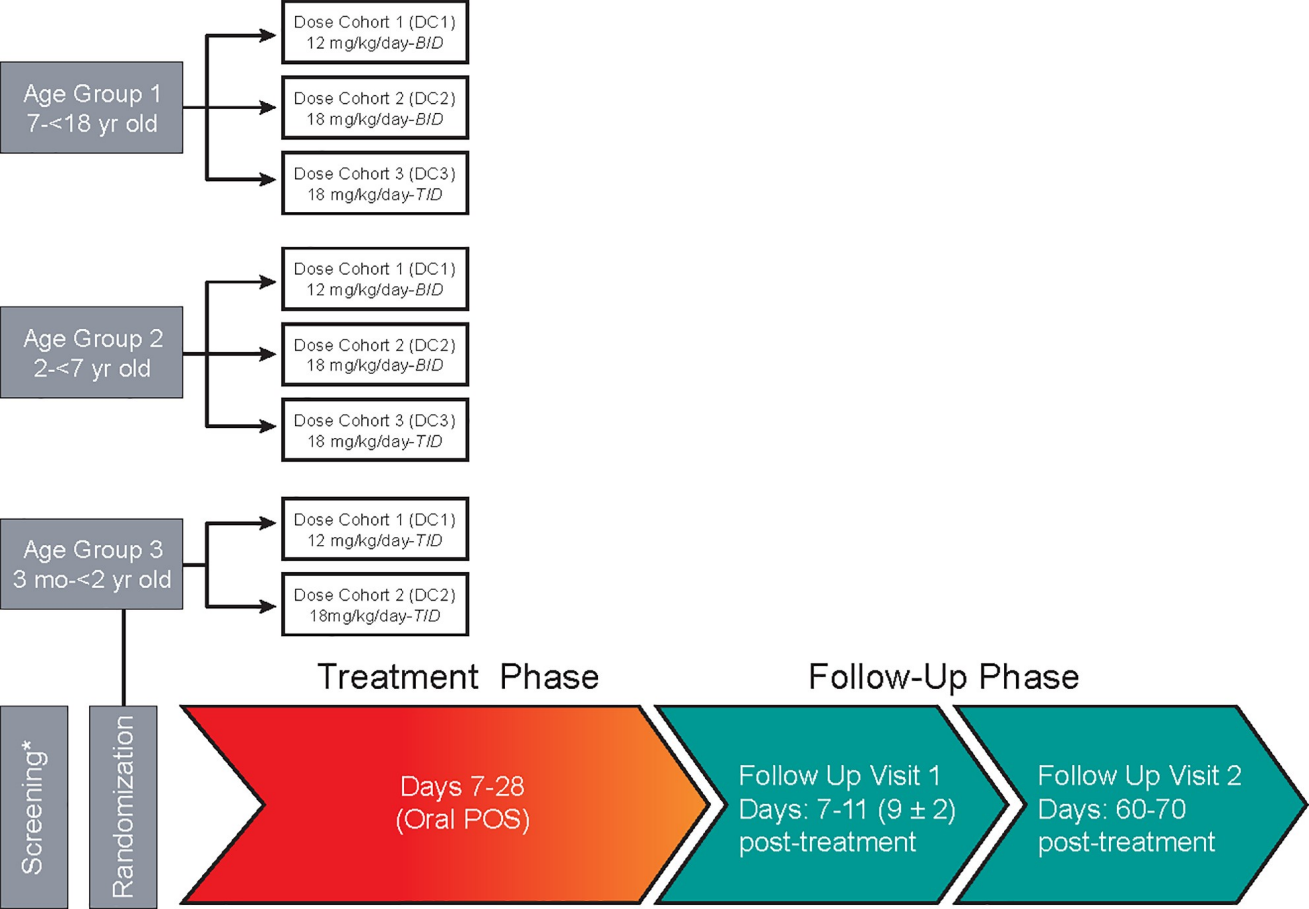
Study design. * Initial screening phase lasted up to 7 days; vital signs, medical history, and blood draws for hematology and serum chemistry assessments were taken.# Minimum duration of treatment was 7 days and continued until resolution of neutropenia or initiation of SOC treatment for IFI, up to a maximum of 28 days. Note: Blood draws for full PK assessments were obtained on Day1 and Day 7, immediately prior to oral administration of POS. POS trough samples were also obtained immediately prior to dosing on Days 3, 5, 8, 14, AND 28 or within 24 hours after the last dose of study drug for earlier discontinuations.

Subjects were stratified into 3 age groups and 3 dosing cohorts as shown in **Fig 1**. Age Group 1 (AG1) consisted of subjects 7 to <18 years of age; Age Group 2 (AG2), subjects 2 to <7 years of age; and Age Group 3 (AG3), subjects 3 months to <2 years of age. AG1 and AG2 were divided into 3 sequential dosing cohorts (DCs): DC1, 12 mg/kg/day divided twice daily (BID), up to a maximum of 800 mg/day; DC2, 18 mg/kg/day divided twice daily (BID), up to a maximum of 1200 mg/day; and DC3, 18 mg/kg/day divided 3 times daily (TID), up to a maximum of 1200 mg/day.

Enrollment in AG3, the youngest subjects [3 months to <2 years], was not initiated until enrollment in the first 2 dosage cohorts in AG1 and AG2, and a preliminary review of safety and PK data was completed. The data from the subjects administered POS oral suspension at a dose of 12 mg/kg/day BID and 18 mg/kg/day BID informed which of the planned doses would be used for AG3. In AG3, the planned dosage cohorts were 12 mg/kg/day of oral POS divided TID (DC1) and 18 mg/kg/day of oral POS divided TID (DC2). All administrations in AG3 were provided orally or via enteral tube; the feasibility of administration via enteral tube was tested by the Sponsor prior to initiation of AG3. Subjects were provided a high fat meal or snack prior to dosing and most subjects did consume some food majority in the medium and high fat categories.

Each subject who enrolled in the study participated in the trial for approximately 80 days from the time the subject signed the informed consent form (ICF) through the final contact. As shown in **Fig 1**, an initial screening phase of up to 7 days was planned, during which vital signs, medical history, and blood draws for hematology and serum chemistry assessments were taken. At baseline, a physical examination with vital signs, body weight, electrocardiogram (ECG), and measurement of ANC were performed. This was followed by a treatment phase during which subjects received study drug for a minimum of 7 days and a maximum of 28 days; in general, treatment continued until recovery from neutropenia (absolute neutrophil count [ANC] >500/mm^3^) or until initiation of standard of care antifungal therapy. During the first 7 days of the treatment phase, vital signs and adverse events were assessed every 2 days, followed by weekly assessments for ongoing treatment through Day 28. Blood draws for serum chemistry, hematology, and PK were performed on the same schedule. At the end of the treatment phase there were two follow-up visits; a first follow-up visit performed 9 (±2) days after treatment ended, and a second follow-up visit on Day 65 (±5 days), which focused on an assessment of survival.

### Patient population

Eligible subjects were required to have documented or anticipated neutropenia (ANC ≤500/mm^3^) expected to last for at least 7 days. In addition, subjects were required to be recipients of an autologous or allogeneic HSCT or have an eligible cancer diagnosis. Eligible diagnoses included acute leukemia (new or relapse), myelodysplastic syndrome, severe aplastic anemia, high risk neuroblastoma, or advanced stage non-Hodgkin’s lymphoma. Recipients of allogeneic HSCT were eligible during the pre-engraftment (neutropenic) period. Subjects with proven IFIs at baseline, as defined by the European Organization for Research and Treatment of Cancer- Mycoses Study Group criteria [14], were excluded from the study. Other exclusion criteria were severe nausea and/or vomiting at screening (grade 3 or 4); study drug (POS) administration within 10 days before screening; inability to receive study drug by mouth or enteral tube; significant elevations of transaminase (>5x upper limit of normal (ULN)) and/or bilirubin levels at baseline (>2.5x upper limit of normal (ULN)); significant renal dysfunction at baseline (Calculated creatinine clearance <30 mL/min.); and history of anaphylaxis attributed to the azole class of antifungal agents. Medications known to interact with azoles and potentially leading to life-threatening adverse effects (such as astemizole, cisapride, beastie, halofantrine, pimozide, quinidine, and terfenadine) and medications known to affect the serum concentration/efficacy of triazole antifungal agents (barbiturates, carbamazepine, cimetidine, isoniazid, phenytoin, rifabutin, rifampin, and St. John’s wort (*Hypericum perforatum*) were prohibited.

### Assessments

Blood samples for PK assessment of POS were obtained on Days 1 and 7 immediately prior to oral administration of POS and at 3, 5, 8, and 12 hours from the time of oral administration of the morning dose. For the TID dosage cohorts, the 12-hour sample was not collected, and the 8-hour sample was drawn prior to the next dose. In addition, POS trough samples (C_min_) were obtained immediately prior to dosing on Days 3, 5, 8, 14, and 28, or within 24 hours after the last dose of study drug for earlier discontinuations. For subjects weighing <6.5 kg, Day 1 blood samples were not collected in order to minimize blood collection in this group. Approximately 1 mL blood was collected at each PK time point for each subject and plasma samples were sent to a central laboratory for analysis. For the POS assay, a validated liquid chromatography coupled to tandem mass spectrometry detection method [15] was used, with a calibration/validation range of 5 to 5,000 ng/mL. The acceptance criteria was set at ±15%. There were minimal samples above the upper limit of quantitation, but those that exceeded 5000 ng/mL were diluted to fall within the validated range. All dilutions were validated.

### PK evaluations

Subjects who received at least one dose of POS were included in the PK analysis. Key PK measures of POS were C_avg_ or the average plasma concentration; defined as the AUC (calculated using Phoenix v.6.3 software, that utilizes the linear-linear trapezoidal method for ascending concentrations up to C_max_ and log-linear trapezoidal method for descending concentrations) divided by the dosing interval on Day 7 (which was considered steady state); AUC time curve from time zero to the time of the final quantifiable sample (AUC_tf_); maximum [peak] plasma concentration (C_max_); time to C_max_ (T_max_); and ratio of AUC_tf_ on Day 7 to AUC_tf_ Day 1. The POS PK target was predefined as ~90% of subjects with C_avg_ between 500 and 2500 ng/mL, with an anticipated mean steady state C_avg_ exposure of ~1200 ng/mL. All PK parameters were calculated using non-compartmental methods with Phoenix WinNonlin 6.3. AUC_tf_ was calculated using the linear trapezoidal method for ascending concentrations and the log trapezoidal method for descending concentrations. C_max_ and T_max_ were obtained by inspection of the plasma concentration data.

### Safety

Safety assessments were conducted on all subjects that received at least one dose of the study drug. These assessments included reports of all adverse events (AEs). Adverse events were classified as treatment-emergent AEs (TEAEs), treatment-related (or drug-related) TR-TEAEs, AEs leading to discontinuation of study drug, and SAEs. Treatment emergent adverse events were defined as an untoward medical occurrence in a subject administered a pharmaceutical product, which does not necessarily have a causal relationship with the treatment and occurred *after start of treatment* with posaconazole. TR-TEAEs were defined as TEAEs judged by the investigator to be related to treatment with posaconazole. Serious adverse events (SAE) were defined as any adverse drug experience occurring at any dose that results in any of the following outcomes: death; life-threatening AE; persistent or significant disability/incapacity; requires in-patient hospitalization or prolongs hospitalization; congenital anomaly or birth defect. Additionally, important medical events that may not result in death, be life-threatening, or require hospitalization may be considered an SAE when, based upon appropriate medical judgment, they may jeopardize the subject and may require medical or surgical intervention to prevent one of the outcomes listed in this definition.

SAEs were captured from the time of signing of the ICF through 30 days after the administration of the last dose of study drug. All other AEs were collected from screening through the first follow-up study visit, which occurred 9 (±2) days after the last dose of study drug. A survival assessment was conducted at the second follow-up visit, on Day 65 (±5 days). TEAEs and TR-TEAEs were tabulated by body system/organ class, with summaries tabulated for each age group/dosage cohort. In addition to AE reporting, electrocardiography (ECG) was performed at baseline and Day 3; ECG summary statistics included the numbers of subjects with change from baseline in QTcF categorized as <0, 0 to 30, 31 to 60, and >60 msec and the number of subjects with a treatment-emergent QTcF interval >500 msec. Laboratory safety reporting based on hematology and serum chemistries were assessed at screening on Days 1, 3, 5, 7, 14, 21, and 28 of treatment, at end of treatment, and at the first follow-up visit.

### Statistical analysis

For C_avg_, the proportion of subjects achieving the target exposure between 500 and 2500 ng/ml was calculated for the comparison of proportions across age group and dosage cohort. A comparison between adult and pediatric subjects with respect to the proportion of PK evaluable subjects (as defined in S1 File) achieving the target exposure was also conducted. For C_avg,_ the arithmetic mean and its %CV and SD) was assessed. For the other PK parameters (C_avg_, C_max,_ T_max,_ and AUC_tf_), summary statistics were provided, including medians (min, max) for each of these parameters as well as the accumulation ratio for AUC (AUC_tf_ Day 7/AUC_tf_ Day 1). Log-transformed ratio estimates with 90% confidence intervals were calculated for the differences between doses and ages. The steady-state analysis was conducted using available PK (C_min_) trough values.

Preliminary PK data were reviewed from DC1 and DC2 in the 2 older age groups, AG1 and AG2. Subsequently, an analysis on DC1 in AG3 and DC3 in AG1 and AG2 were conducted. This manuscript summarizes the data from all of these analyses.

## Results

### Population

**Fig 2:**
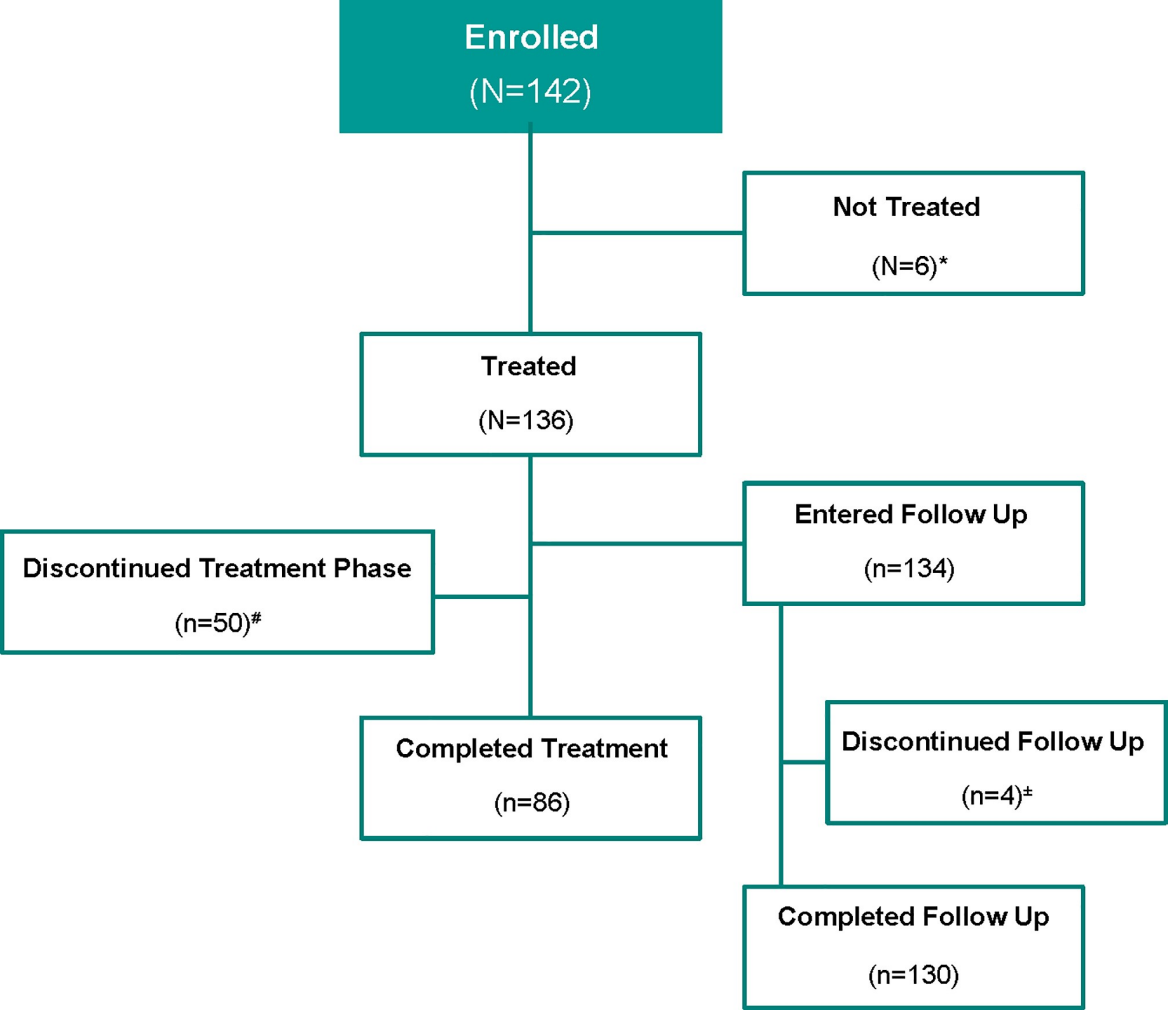
Subject disposition. * Of the 6 subjects not treated, 4 subjects did not wish to continue due to reasons unrelated to assigned study treatment, 1 subject did not meet protocol eligibility, and 1 subject had an adverse event (AE). ^#^ Most common reasons for discontinuation of the treatment phase were: AE (n = 35), subject not willing to continue due to reasons unrelated to treatment (n = 7), subject not meeting protocol requirement to continue therapy (due to recovery from neutropenia before 7 days of therapy, n = 4) and non-compliance with protocol (n = 3). ^±^ Three subjects discontinued follow up due to an AE, and one subject was non-compliant with the protocol.

The disposition of the subjects in the study is shown in **Fig 2**. A total of 142 subjects were enrolled in the study; of these, 136 subjects received at least one dose of POS. There were a total of 86 (63%) treated subjects who completed the treatment phase of the study; i.e., they no longer required prophylaxis [having recovered from neutropenia after at least 7 days of study drug] or received up to a maximum of 28 days of study drug. Among the 50 (37%) treated subjects that discontinued in the treatment phase, the most common reason for discontinuation was an AE (35 subjects, 26%), followed by discontinuation for reasons unrelated to study treatment (7 subjects, 5%). Only 2 of the 136 treated subjects did not complete the first follow-up visit, at day 9 (±2) post-treatment. A total of 130 (97%) subjects completed the study through the second follow-up visit.

A summary of demographic data by age group and dosage cohort is provided in **Table 1**. Of the 136 treated subjects, the majority were male (58%) and were white (88%). The overall median age was 8.5 years, and the overall median weight was 29.8 kg. Only 1 subject was enrolled in AG3, as the study was stopped prematurely once there was evidence that the target PK could not be achieved in the 2 older age groups. Overall, slightly more than half of the subjects had a primary diagnosis of acute leukemia (52%), followed by HSCT (24%), neuroblastoma (10%), and non-Hodgkin’s lymphoma (7%). Of the subjects with a primary diagnosis of acute leukemia, nearly twice as many subjects had AML as compared to ALL (32% versus 17%, respectively).

**Table 1:** Subject characteristics across different age groups and posaconazole dosing cohorts.

	AG1: 7 to <18 y			AG2: 2 to <7 y			AG3: 3 mo to <2 y	Total
	DC1: 12 mg/kg/d (BID) n (%)	DC2: 18 mg/kg/d (BID) n (%)	DC3: 18 mg/kg/d (TID) n (%)	DC 1: 12mg/kg/d (BID) n (%)	DC2: 18 mg/kg/d (BID) n (%)	DC3: 18mg/kg/d (TID) n (%)	DC1: 12 mg/kg/d (TID) n (%)	N(%)
Treated	21 (100)	28 (100)	30 (100)	22 (100)	19 (100)	15 (100)	1 (100)	136 (100)
Sex								
**Female**	11 (52)	9 (32)	14 (47)	9 (41)	8 (42)	6 (40)	0	57 (42)
**Male**	10 (48)	19 (68)	16 (53)	13 (59)	11 (58)	9 (60)	1 (100)	79 (58)
Race, n (%)								
**White**	17 (81)	22 (79)	26 (87)	20 (91)	18 (95)	15 (100)	1 (100)	119 (88)
**Non-White**	4 (19)	6 (21)	4 (13)	2 (9)	1 (5)	0	0	17 (13)
**Asian**	1 (5)	3 (11)	1 (3)	2 (9)	0	0	0	7 (5)
**Black or African**	1 (5)	2 (7)	2 (7)	0	1 (5)	0	0	6 (4)
**Multiracial 0**	2 (10)	1 (4)	1 (3)	0	0	0	0	4 (3)
Primary Diagnosis								
**Acute Leukemia**	11 (52)	15 (54)	20 (67)	8 (36)	9 (47)	7 (47)	1 (100)	71 (52)
**ALL**	5 (24)	4 (14)	5 (17)	4 (18)	2 (11)	3 (20)	0	23 (17)
**AML**	6 (29)	11 (39)	14 (47)	3 (14)	7 (37)	3 (20)	0	44 (32)
**Unspecified**	0	0	1 (3)	1 (5)	0	1 (7)	1 (100)	4 (3)
**Myelodysplastic Syndrome**	2 (10)	0	0	0	2 (11)	0	0	4 (3)
**Aplastic Anemia**	0	3 (11)	0	1 (5)	0	0	0	4 (3)
**Hematopoietic Stem Cell Transplantation**	5 (24)	5 (18)	6 (20)	5 (23)	7 (37)	5 (33)	0	33 (24)
**Allogeneic**	0	0	0	0	2 (11)	2 (13)	0	4 (3)
**Autologous**	1 (5)	1 (4)	0	0	1 (5)	0	0	3 (2)
**Unspecified**	4 (19)	4 (14)	6 (20)	5 (23)	4 (21)	3 (20)	0	26 (19)
**Neuroblastoma**	1 (5)	1 (4)	0	8 (36)	1 (5)	2 (13)	0	13 (10)
**Non-Hodgkin’s Lymphoma**	1 (5)	4 (14)	4 (13)	0	0	1 (7)	0	10 (7)
**Missing**	1 (5)	0	0	0	0	0	0	1 (1)

ALL = acute lymphocytic leukemia; AML = acute myelogenous leukemia; BID = twice daily; TID = three times daily

n = number of subjects.

### PK evaluations

A summary of the percentage of PK evaluable subjects who achieved the PK target (C_avg_ at steady state [Day 7] between 500 and 2500 ng/mL) is presented in **Table 2** by age group and dosage cohort. Overall, 70 PK-evaluable subjects were available for assessment at steady state. None of the dosage cohorts in this study achieved the target PK of 90% of subjects with C_avg_ between 500 and 2500 ng/mL; 30 of the 70 PK-evaluable subjects (43%) had steady-state C_avg_ level <500 ng/ml, while only 3 (4%) subjects had C_avg_ ≥2500 ng/mL. Furthermore, there were no data to support a trend of higher steady-state C_avg_ exposure with either increasing the dose (from 12 to 18 mg/kg) or frequency of administration (from BID to TID) of POS oral suspension.

**Table 2:** Distribution of C_avg_ by age group and dose cohort in subjects with evaluable PK on day 7.

Age & Dose Cohorts			C_avg_ (ng/ml)				
Age Group	Dose Cohorts	N	< 200 % (n/m)	200 to < 500 % (n/m)	500 to <2500 % (n/m)	2500 to < 3650 % (n/m)	>3650 % (n/m)
**AG1:** **(7 to 18 years)**	**DC1:** 12 mg/kg/day (BID)	14	14% (2/14)	21% (3/14)	64% (9/14)	0	0
	**DC2:** 18 mg/kg/day (BID)	12	8% (1/12)	25% (3/12)	50% (6/12)	8% (1/12)	8% (1/12)
	**DC3:** 18 mg/kg/day (TID)	10	20% (2/10)	0	80% (8/10)	0	0
**AG2:** **(2 to <7 years)**	**DC1:** 12 mg/kg/day (BID)	16	19% (3/16)	44% (7/16)	31% (5/16)	6% (1/16)	0
	**DC2:** 18 mg/kg/day (BID)	12	25% (3/12)	25% (3/12)	50% (6/12)	0	0
	**DC3:** 18 mg/kg/day (TID)	5	20% (1/20)	20% (1/20)	60% (3/5)	0	0
**AG3:** **(3 months to <2 years)**	**DC1:** 12 mg/kg/day (TID)	1	0	100% (1/1)	0	0	0

Numbers in parentheses = (Number of subjects in category / Total number of subjects)

Target C_avg_ range (500-<2500 ng/ml) required for ~90% of subjects to meet criteria for study success

The percentage of subjects that achieved the C_avg_ target of 500 to 2500 ng/mL on Day 7 ranged from 31% to 80%, with the lowest proportion in subjects 2 to <7 years receiving 12 mg/kg/day BID (AG2/DC1) and the highest proportion in subjects 7 to <18 years receiving 18 mg/kg/day TID (AG1/DC3). The one subject enrolled in the youngest age group (3 months to <2 years age, AG3) also did not meet the target.

**Fig 3:**
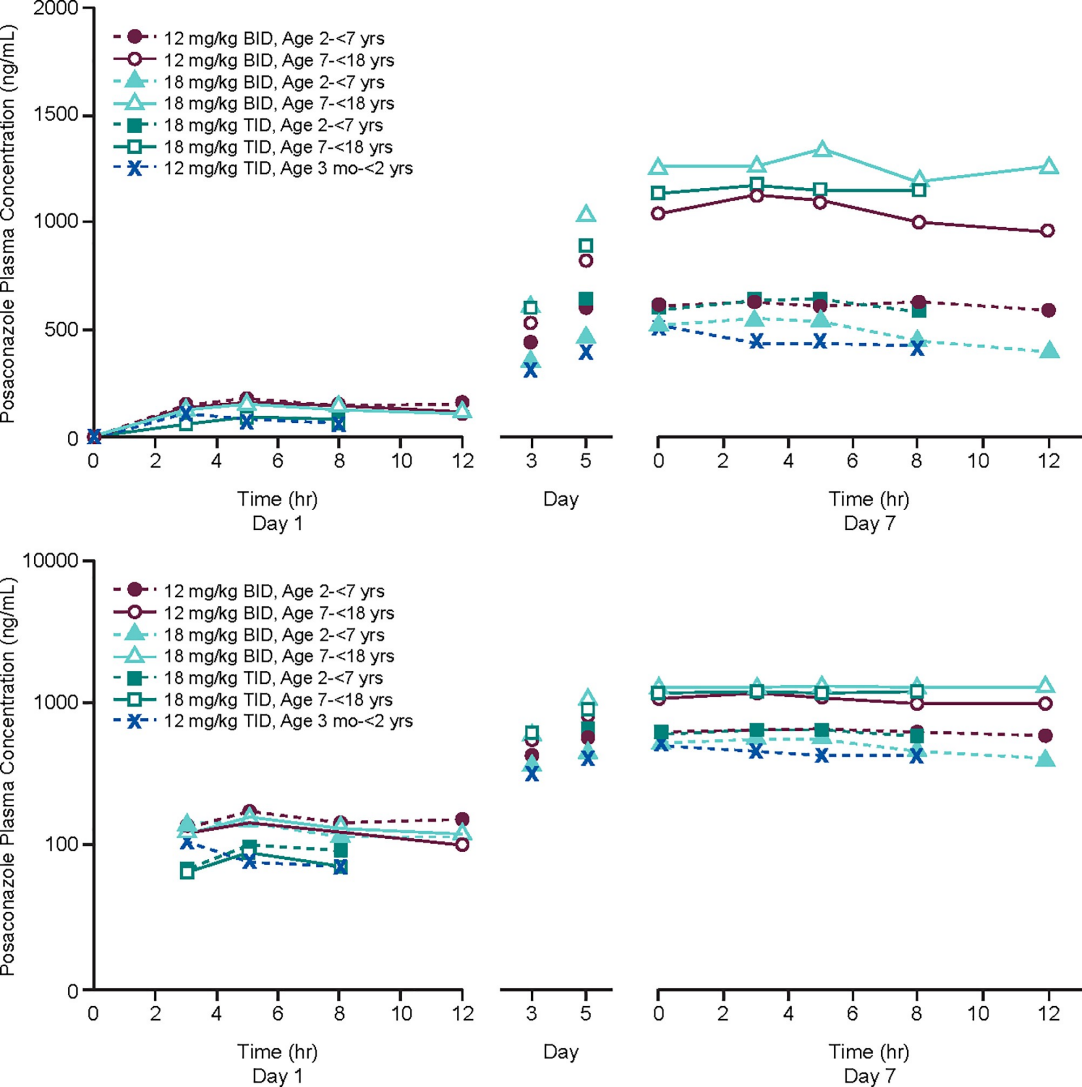
Combined Mean plasma concentration profile by age group and dosing cohort following single (day 1) and multiple dose (day 7) administration of posaconazole oral suspension.

An assessment of all the PK parameters for the various age groups and dose cohorts are summarized in **Table 3.** The combined mean (±SD) plasma concentration profiles of POS in all age groups and dose cohorts are shown in **Fig 3**. At all three dose levels (12 mg/kg/day BID, 18 mg/kg/day BID, and 18 mg/kg/day TID), subjects in AG1 (7 to <18 years old) had higher mean PK exposures at steady state than those in AG2 (2 to <7 years old). The Day 1 PK exposures were similar between the two age groups at all three dose levels. No definitive conclusions can be drawn in AG3 (3 months to <2 years) as there was only 1 subject in this group.

**Table 3:** Pharmacokinetic parameter values of posaconazole by age group and dosing cohort following single and multiple dose administration of posaconazole oral suspension.

	AG1: 7 to 18 y						AG2: 2 to <7 y						AG3: 3 mo to <2 y	
	DC1: 12 mg/kg/d (BID)		DC2: 18 mg/kg/d (BID)		DC3: 18 mg/kg/d (TID)		DC1: 12 mg/kg/d (BID)		DC2: 18 mg/kg/d (BID)		DC3: 18 mg/kg/d (TID)		DC1: 12 mg/kg/d (TID)	
Day	1	7	1	7	1	7	1	7	1	7	1	7	1	7
n	19	14	12^d^	12	10	10	22	16^c^	12	12	5	5	1	1
C_max_ (ng/mL)	156 (78.1)	1200 (75.5)	162 (86.7)	1390 (111.4)	93.2 (60.8)	1230 (64.2)	196 (93.9)	726 (125.5)	175 (70.5)	581 (61.0)	109 (61.3)	705 (60.9)	103	520
T_max_^a^ (hr)	5.0 (2.97, 12.0)	4.58 (0, 7.75)	3.12 (2.92, 8.00)	4.03 (0.0, 28.5)	4.88 (2.92, 8.08)	2.63 (0.00, 7.62)	5.01 (2.92, 11.60)	4.13 (0.0, 11.17)	3.99 (2.98, 11.08)	3.00 (0.0, 8.08)	7.95 (2.98, 8.00)	3.00 (0.0, 5.08)	3.38	0.00
AUC_tf_ (hr*ng/mL)	1140 (93.7)	11,800 (75.4)	1270 (98.1)	13500 (115.8)	424 (49.5)	8310 (74.9)	1300 (91.4)	6770 (138.9)	1210 (76.88)	5350 (62.0)	544 (59.6)	4920 (67.1)	574	3590
tf^a^ (hr)	11.88 (7.92, 12.25)	11.59 (7.95, 12.08)	11.54 (2.92, 12.08)	11.60 (7.33, 12.12)	7.92 (4.83, 8.10)	7.77 (4.67, 8.00)	11.04 (7.98, 12.15)	11.42 (8.00, 12.00)	11.23 (9.07, 12.07)	11.5 (8.0, 12.03)	7.95 (7.83, 8.00)	7.92 (7.82, 8.00)	8.38	7.92
Mean C_avg_^b^ (ng/mL)	107 (86.5, 92.5)	1050 (76.2, 789)	113 (89.1, 100)	1240 (113.4, 1400)	57.9 (52.2, 30.2)	1150 (65.4, 750)	122 (83.1, 101)	604 (129.0, 779)	112 (77.6, 86.9)	485 (63.0,306)	68.4 (59.2, 40.4)	620 (66.2, 411)	68.5	453
Median C_avg_^a^ (ng/mL)	89.7 (16.4, 398)	979 (65.4, 2420)	74.4 (33.8, 393)	698 (181, 4660)	56.2 (12.0, 105)	1300 (127, 2340)	94.7 (15.7, 437)	414 (37.8, 3350)	519 (48.3, 926)	519 (48.3, 926)	75.4 (28.10, 124)	529 (191, 1280)	68.5	453
R	–	11.2 (69.8)	–	11.7 (75.8)	–	22.0 (70.8)	–	4.42 (47.9)	–	5.33 (80.8)	–	12.0 (73.0)		6.25

n: Number of subjects; C_max_: maximum concentration; T_max_: time to maximum observed concentration; AUCtf: area under the concentration-time curve from time 0 to the time of the final quantifiable sample; tf: last time point with PK sample collected and concentration >LLOQ; C_avg_: AUCtf/tf; R: accumulation ratio (AUCtf Day 7/AUCtf Day 1).

a Median (min-max).

b Arithmetic mean (%CV, SD).

c One subject had quantifiable pre-dose concentration greater than 10% C_max_.

d One subject had the first dose split into two and the second half of the dose was administered 3.25 hours after the first half of the dose. PK exposure was calculated based on PK sample collection time elapsed from the first half of the dose.

### Safety

A total of 136 subjects received at least one dose of study drug and were included in the study safety analyses. TEAEs, treatment-related TEAEs, SAEs, treatment-related SAEs, and AEs leading to early study discontinuation are summarized in **Table 4.** Overall, TEAEs and TR-TEAEs were reported in 94% (n = 128) and 37% (n = 50) of subjects, respectively. The most common TR-TEAEs observed in ≥5% of subjects overall were nausea (13%) and vomiting (13%). Treatment-related severe/life threatening (LT) TEAEs were reported for 14 (10%) subjects. The most commonly reported treatment-related severe/LT TEAEs (representing ≥2% of subjects overall) were alanine aminotransferase increased (ALT, 4%) and aspartate aminotransferase increased (AST, 2%). Discontinuations of the study treatment due to an AE occurred in 26% of the treated subjects, with the highest rate observed with the lowest dose, (37% in those receiving POS 12 mg/kg/day BID). Serious AEs were reported for 35 subjects (26%), with the highest frequency (40%) observed at the highest dose, POS 18 mg/kg/day TID. A diverse variety of SAEs were reported, with most events pertaining to the underlying disease or complications relating to the underlying condition. The most common reported SAE was febrile neutropenia in 15 subjects (11%), of which the majority (10 subjects; 22%) were in the POS 18 mg/kg/day TID group. Only 3 subjects, including 2 (5%) receiving POS 12 mg/kg/day BID (both in AG2, the 2 to <7-year age group) and 1 (2%) receiving POS 18 mg/kg/day TID (in AG1, the 7 to <18-year group), experienced SAEs considered by the investigator as treatment related. In AG2/DC1 subjects (n = 2), treatment related SAEs included: electrocardiogram T-wave inversion, increased levels of alanine aminotransferase (ALT), increased aspartate aminotransferase (AST), and increased chemotherapeutic (methotrexate) drug levels. The single treatment-related SAE in an AG1/DC3 subject was inversion of the electrocardiogram T wave.

**Table 4:** Summary of Safety by age group and dose cohorts and most common treatment-related adverse events by age group.

	AG1: 7 to <18 y			AG2: 2 to <7 y			AG3: 3 mo to <2 y	Total
	DC1: 12 mg/kg/d (BID) n (%)	DC2: 18 mg/kg/d (BID) n (%)	DC3: 18 mg/kg/d (TID) n (%)	DC1: 12 mg/kg/d (BID) n (%)	DC2: 18 mg/kg/d (BID) n (%)	DC3: 18 mg/kg/d (TID) n (%)	DC3: 12 mg/kg/d (BID) n (%)	
Treatment- emergent adverse event (TEAE)	21 (100)	26 (93)	30 (100)	21 (95)	16 (84)	13 (87)	1 (100)	128 (94)
Treatment-related TEAE (TR-TEAE)	5 (24)	16 (57)	10 (33)	11 (50)	5 (26)	3 (20)	0	50 (37)
Serious adverse event (SAE)	3 (14)	7 (25)	11 (37)	5 (23)	1 (5)	7 (47)	1 (100)	35 (26)
Death	0	1 (4)	1 (3)	0	0	1 (7)	0	3 (2)
Severe/life-threatening TEAE	10 (48)	16 (57)	20 (67)	14 (64)	5 (26)	7 (47)	0	72 (53)
Study drug discontinuation due to AE	9 (43)	9 (32)	6 (20)	7 (32)	2 (11)	3 (20)	0	36 (26)
**Treatment-Related Treatment–Emergent Adverse Events (TR-TEAE) of >5% Frequency**								
	**AG1: 7 to <18 y** **(N = 79)**			**AG2: 2 to <7 y** **(N = 56)**			**AG3: 3 mo to** **< 2 y** **(N = 1)**	**Total** **(N = 136)**
Any Adverse Event	31 (55)			19 (24)			0	50 (37)
Nausea	14 (18)			4 (7)			0	18 (13)
Vomiting	11 (14)			6 (11)			0	17 (13)
Abdominal Pain	4 (5)			2 (4)			0	6 (4)
Diarrhea	4 (5)			1 (2)			0	5 (4)
Stomatitis	1 (1)			3 (5)			0	4 (3)
ALT increased	5 (6)			4 (7)			0	9 (7)
AST increased	3 (4)			3 (5)			0	6 (4)

n: number of subjects; POS: posaconazole; BID = two times per day; TID = three times per day

Note: Deaths are also included in serious adverse event count.

There were also three reported deaths across all treated subjects, including 2 in the POS 18 mg/kg/day TID group (DC3) and 1 in the POS 18 mg/kg/day BID group (DC2). In DC3, 1 death was related to Burkitt’s lymphoma occurring on Day 114 (95 days after last dose of POS) and the other death was related to multi-organ failure on Day 132 (122 days after last dose of POS). In DC2, the 1 death occurred on Day 65 (58 days after the last dose of POS) as a result of acute respiratory distress syndrome and multi-organ failure. None of the deaths were considered by the investigators as related to POS.

The frequency of TR-TEAEs reported in ≥5% of subjects within any age group was generally similar between the 2 older age groups (AG 1 [7to <18 years] and AG2 [2to <7years]). One notable exception was a higher frequency of nausea (18% vs. 7%) reported in AG1 vs. AG2. Overall, there was no apparent pattern to suggest a difference in the safety profiles among the three dosing cohorts and 2 older age groups. Safety in AG3 (3 months to <2 years) could not be assessed, with only 1 subject treated in this youngest age group.

No safety concerns were noted in other safety parameters, including ECG measurements and laboratory testing. No subject met the pre-specified protocol criteria for significant QT effect (QTc >500 msec). Laboratory abnormalities of interest were increased ALT in 14/133 subjects (11%), increased AST in 8/133 subjects (6%), and increased creatinine in 2/133 subjects (2%).

## Discussion

The PK and safety of the POS oral suspension in the pediatric population was characterized in this study. POS oral suspension was administered at dosages of 12 mg/kg/day (DC1) in two divided doses, and 18 mg/kg/day in two (DC2) and three divided doses (DC3). Despite assessments at different dosages and varying dose intervals, the POS oral suspension failed to achieve the PK target for the study (i.e., >90% of pediatric subjects with a steady state C_avg_ of 500 ng/ml– 2500 ng/ml). Nonetheless, some individual subjects did attain the target levels of 500 to 2500 ng/mL on Day 7 with the lowest proportion in AG2(2y to <7 y) /DC1(12mg/kg/d, BID) subjects and the highest proportion in subjects being AG1(7y to <18 y) /DC3(18mg/kg/d, TID). At all three dose levels, subjects in AG1(7y to <18y) had higher mean PK exposures at steady state than those in AG2(2y to < 7y).

The PK target was based on previous identification of steady state C_avg_ as a key PK parameter for POS, based on data from the adult prophylaxis and treatment studies in which efficacy was correlated with certain C_avg_ exposures (16). The target level of the steady-state POS plasma concentration was based on observations from earlier registration clinical trials conducted with the POS oral suspension in adult patients, in which POS plasma concentrations at steady state appeared to be correlated with efficacy in both the prophylaxis and treatment settings. Using data from adult pivotal trials, a mean steady state C_avg_ exposure of approximately 1200 ng/mL with approximately 90% of subjects with values between 500 and 2500 ng/mL was proposed as a target exposure for the POS pediatric program. As POS has a long terminal-phase half-life of ~35 hours, steady-state is achieved by 7 days with a relatively flat plasma concentration-time profile. Data in healthy adult volunteers [13] demonstrate that at steady-state, any concentration within a dosing interval when multiplied by the dosing interval, reliably estimates POS AUC. In addition, at steady-state, the fluctuation between POS maximum observed concentration (C_max_) and minimum observed plasma concentration (C_min_ or trough) is relatively small. Thus, the steady-state mean trough concentrations, C_avg_, and C_max,_ are essentially the same for POS, thereby allowing for C_avg_ to be selected in clinical studies as the primary PK parameter of interest. In a controlled trial involving neutropenic adult subjects (study P01899), POS oral suspension was superior to standard triazoles (fluconazole and itraconazole) in reducing the incidence of IFI, including aspergillosis and candidiasis, and in reducing 100-day all-cause mortality. In that study, the mean plasma concentration at steady state was 583 ng/mL [6]. In another study of IFI treatment (P00041), POS oral suspension was also found to be effective for the treatment of adult subjects with refractory IFI, including aspergillosis, and for the treatment of IFI in adult subjects who were intolerant to certain antifungals, with a better response rate and survival benefit when compared to historical control cases [16]. In that study, the mean plasma concentration was 808 ng/mL. An association between POS plasma concentration achieved with POS oral suspension at steady state and efficacy in these pivotal clinical trials was identified, with adult subjects with POS steady state exposures >500 ng/mL having higher response rates than controls [16]. Hence, a minimum C_avg_ was selected for the pediatric study summarized herein.

In the current study, the percentage of subjects meeting the target C_avg_ range of 500–2500 ng/mL was low across all age groups and dose cohorts, ranging from 31% in AG2 (2y to <7 y) and DC1, (12mg/kg/d, BID) to 80% in AG1 (7y to <18 y) and DC3 (18 mg/kg/d, TID). There was no clear trend toward higher exposure with increasing the daily dose, nor with increasing the frequency of dosing. The TID regimen had higher probability of target attainment regardless of age group; this finding was more apparent in AG1 (7y to <18y) which had 80% DC3 (18mg/kg/d, TID) compared to 50% DC2 (18mg/kd/d, BID) and 64% DC1 (12 mg/kd/d, BID, thus suggesting faster, less predictable clearance in younger children. Although geometric mean C_avg_ confidence intervals were substantially wider among both age groups due to limitations in sample size (**Table 2**), there is an apparent trend of lower C_avg_ among the younger age group (AG2, 2 to <7 years). Overall, these results are not different from those seen in previously published data regarding the use of POS oral suspension in adults [6]; in one adult study, only 49% of subjects achieved steady state exposures within the target C_avg_ range [17]. The adult C_avg_ reported in the earlier study fell within the range of the younger age group included in the current pediatric study, though it is not certain that the doses are directly comparable, as the adult subjects were administered doses ranging from 600 mg to 1200 mg/day (divided TID). As with other triazole antifungals, POS is a known CYP3A4 inhibitor and is also a substrate and inhibitor for Pgp-mediated transport. However, it is unlikely that these interactions would result in the differences in PK exposure across age groups at steady-state, given the similar exposure seen across age groups in the current study on Day 1. Finally, it is worth noting that food intake has been shown to increase the PK exposure of POS oral suspension in adults by 3–4 fold [13]. The food intake of the pediatric subjects enrolled in the present study was monitored by the subject or legal guardian and there were, as expected, slight variances between subjects. However, it is unclear if the slight differences in food intake could lead to the observed differences in PK exposures across age groups. Ultimately, due to the small sample size and early termination of the study, the cause for the higher exposures in AG 1 (7 to <18 years) compared to AG2 (2 to <7 years) is inconclusive.

Some pediatric subjects were included in the earlier POS trials, thereby helping to inform the starting dosage of 12 mg/kg/day in the current study. In the adult IFI treatment study (P00041), all adult subjects received 800 mg POS/day, corresponding to 11 mg/kg/day for a 70-kg adult. The majority of pediatric subjects (8 to 17 years of age) enrolled in this study received the full adult dosing regimen, resulting in a range of 15 mg/kg/day to 24 mg/kg/day. Despite slight differences in dose, the C_avg_ achieved in adults (844 ng/mL) was similar to the C_avg_ achieved in the pediatric subjects (776 ng/mL) in this trial. In two prior prophylaxis studies (CI98-316 and P01899) [6], all subjects received 600 mg/day, corresponding to a dose of 8.5 mg/kg/day for a 70-kg adult. This dose provided similar exposure in adults (C_avg_ = 578 ng/mL) as in pediatric subjects (13 to 17 years of age; C_avg_ = 694 ng/mL). Given that the systemic exposure to POS was similar between adolescent subjects and adults, and the exposures did not appear to be influenced by age, weight, or body surface area, it was anticipated that the starting dose of 12 mg/kg/day in the current pediatric study would result in exposures that are similar to those observed in the pediatric and adult populations at this approximate dose, and for which efficacy had already been established in adults. In adult healthy volunteers, dosing above 800 mg/day resulted in a plateau in POS exposure due to limitations in absorption of POS oral suspension. Similar limitations are also likely to occur in pediatric subjects [14]. Walsh et al found that in a group of patients treated with posaconazole for refractory aspergilosis, the group with the highest clinical response (70%), the average steady-state concentration of posaconazole was 1.25 μg/ml (1250 ng/ml; the highest concentration quartile). Patients with concentrations in the middle two quartiles (average concentrations, 0.5 to 0.7 μg/ml or 500 to 700 ng/ml) were successfully treated 53% of the time (17). Others have suggested a trough of 0.7 mcg/ml (700 ng/ml) as a target for therapeutic drug monitoring while using posaconazole (18).

POS oral suspension was generally well tolerated in pediatric subjects enrolled in this study, with a safety profile similar to that previously reported for this POS formulation [6]. As anticipated, nearly all pediatric subjects treated with POS had one or more TEAEs reported, with 26% of subjects reporting an SAE. This safety profile reflects the underlying severity of illness present in the pediatric oncology population studied. In the setting of a weakened immune system, such patients are at risk not only for a fungal infection but other infections, multi-organ failure, progression of underlying hematologic malignancy, and graft failure. Of the treated subjects, 37% (50/136) had a TEAE reported that was judged to be possibly or probably related to study drug. There was no predominant type of related TEAE, although the most commonly reported terms were those involving the gastrointestinal system. Treatment-related nausea and vomiting were reported in 13% (18/136) and 13% (17/136) of subjects, respectively. There was no apparent pattern that would indicate a clear difference among the dose cohorts or age groups; specifically, treatment-related TEAEs did not appear to be dose related.

In conclusion, the PK target (i.e., a C_avg_ at steady state [Day 7] between 500 and 2500 ng/mL in ~90% of subjects) was not achieved in any of the pediatric age groups across the different dosage cohorts receiving POS oral suspension in this study. However, it is encouraging to note that many patients individually attained target C_avg_ concentrations, while very few patients exceeded 2500 ng/ml (n = 2; 2.9%), and in the older age group an 18 mg/kg/day dose achieved C_avg_ within the desired PK parameter.

Currently, there are no approved dosing recommendations for POS oral suspension for the pediatric population. Given this, clinicians who use posaconazole based on their assessment of benefit-risk in a particular child may consider obtaining plasma concentrations and discussing results with an expert in pediatric fungal infections.

Given the potential for POS to prevent and treat IFIs in the pediatric population, and the generally favorable safety profile established in this study for POS, future pediatric development of POS will need to focus on new formulations of the molecule and dosing regimens with a greater potential to achieve the established PK target (i.e., IV formulation, oral granule) equivalent of the current adult tablet formulation. A recent report investigating the PK using a dosing regimen based on body surface area in the pediatric population has shown promising results [18]. Additional studies using new formulations and dosing regimens of the molecule are currently under investigation.

## Supporting information

S1 Table Independent ethics committee and institutional review board details. (DOCX)

S1 File Criteria for subjects considered to be Day 7 PK Evaluable. (DOCX)

S2 File TREND statement checklist.pdf. (PDF)

S3 File MK-5592-032-or-Prot_Final Redaction.pdf. (PDF)

## References

[1] Wiley JM, Smith N, Leventhal BG, Graham ML, Strauss LC, Hurwitz CA, et al Invasive fungal disease in pediatric acute leukemia patients with fever and neutropenia during induction chemotherapy: a multivariate analysis of risk factors. J Clin Oncol. 1990;8(2):280–6. 10.1200/JCO.1990.8.2.280 . 2299371

[2] Rosen GP, Nielsen K, Glenn S, Abelson J, Deville J, Moore TB. Invasive fungal infections in pediatric oncology patients: 11-year experience at a single institution. J Pediatr Hematol Oncol. 2005;27(3):135–40. . 15750444 10.1097/01.mph.0000155861.38641.ca

[3] Castagnola E, Cesaro S, Giacchino M, Livadiotti S, Tucci F, Zanazzo G, et al Fungal infections in children with cancer: a prospective, multicenter surveillance study. Pediatr Infect Dis J. 2006;25(7):634–9. 10.1097/01.inf.0000220256.69385.2e . 16804435

[4] Hovi L, Saarinen-Pihkala UM, Vettenranta K, Saxen H. Invasive fungal infections in pediatric bone marrow transplant recipients: single center experience of 10 years. Bone Marrow Transplant. 2000;26(9):999–1004. 10.1038/sj.bmt.1702654 . 11100280

[5] Dvorak CC, Steinbach WJ, Brown JM, Agarwal R. Risks and outcomes of invasive fungal infections in pediatric patients undergoing allogeneic hematopoietic cell transplantation. Bone Marrow Transplant. 2005;36(7):621–9. 10.1038/sj.bmt.1705113 . 16044133

[6] Cornely OA, Maertens J, Winston DJ, Perfect J, Ullmann AJ, Walsh TJ, et al Posaconazole vs. fluconazole or itraconazole prophylaxis in patients with neutropenia. N Engl J Med. 2007;356(4):348–59. 10.1056/NEJMoa061094 . 17251531

[7] Hol JA, Wolfs TF, Bierings MB, Lindemans CA, Versluys AB, Wildt de A, et al Predictors of invasive fungal infection in pediatric allogeneic hematopoietic SCT recipients. Bone Marrow Transplant. 2014;49(1):95–101. 10.1038/bmt.2013.136 . 24121212

[8] Johnston DL, Lewis V, Yanofsky R, Gillmeister B, Ethier MC, Mitchell D, et al Invasive fungal infections in paediatric acute myeloid leukaemia. Mycoses. 2013;56(4):482–7. 10.1111/myc.12063 . 23437849

[9] Arendrup MC, Fisher BT, Zaoutis TE. Invasive fungal infections in the paediatric and neonatal population: diagnostics and management issues. Clin Microbiol Infect. 2009;15(7):613–24. 10.1111/j.1469-0691.2009.02909.x . 19673972

[10] Steinbach WJ. Epidemiology of invasive fungal infections in neonates and children. Clin Microbiol Infect. 2010;16(9):1321–7. 10.1111/j.1469-0691.2010.03288.x . 20840541

[11] Tragiannidis A, Roilides E, Walsh TJ, Groll AH. Invasive aspergillosis in children with acquired immunodeficiencies. Clin Infect Dis. 2012;54(2):258–67. 10.1093/cid/cir786 . 22075793

[12] Groll AH, Castagnola E, Cesaro S, Dalle JH, Engelhard D, Hope W, et al Fourth European Conference on Infections in Leukaemia (ECIL-4): guidelines for diagnosis, prevention, and treatment of invasive fungal diseases in paediatric patients with cancer or allogeneic haemopoietic stem-cell transplantation. Lancet Oncol. 2014;15(8):e327–40. 10.1016/S1470-2045(14)70017-8 . 24988936

[13] Merck & Co. I. Posaconazole Prescribing Information. Merck & Co., Inc., Whitehouse Station, NJ: Merck Sharp & Dohme Corp., a subsidiary of Merck & Co., Inc. p. 33.

[14] Maertens J, Cornely OA, Ullmann AJ, Heinz WJ, Krishna G, Patino H, et al Phase 1B study of the pharmacokinetics and safety of posaconazole intravenous solution in patients at risk for invasive fungal disease. Antimicrob Agents Chemother. 2014;58(7):3610–7. 10.1128/AAC.02686-13 24733463 PMC4068540

[15] Shen JX, Krishna G, Hayes RN. A sensitive liquid chromatography and mass spectrometry method for the determination of posaconazole in human plasma. J Pharm Biomed Anal. 2007;43(1):228–36. 10.1016/j.jpba.2006.06.011 . 16859858

[16] Walsh TJ, Raad I, Patterson TF, Chandrasekar P, Donowitz GR, Graybill R, et al Treatment of invasive aspergillosis with posaconazole in patients who are refractory to or intolerant of conventional therapy: an externally controlled trial. Clin Infect Dis. 2007;44(1):2–12. 10.1086/508774 . 17143808

[17] Cornely OA, Helfgott D, Langston A, Heinz W, Vehreschild JJ, Vehreschild MJ, et al Pharmacokinetics of different dosing strategies of oral posaconazole in patients with compromised gastrointestinal function and who are at high risk for invasive fungal infection. Antimicrob Agents Chemother. 2012;56(5):2652–8. 10.1128/AAC.05937-11 22290953 PMC3346642

[18] Vanstraelen K, Colita A, Bica AM, Mols R, Augustijns P, Peersman N, et al Pharmacokinetics of posaconazole oral suspension in children dosed according to body surface area. Pediatr Infect Dis J. 2016;35(2):183–8. 10.1097/INF.0000000000000963 . 26544987

