# Supplementary material for: A non-randomized trial to assess the safety, tolerability, and pharmacokinetics of posaconazole oral suspension in immunocompromised children with neutropenia
Source: PLoS One. 2019 Mar 26;14(3):e0212837. doi: 10.1371/journal.pone.0212837 (PMC6435162; doi:10.1371/journal.pone.0212837)
Supplement: S1 Table — (DOCX) [file pone.0212837.s001.docx]

Supporting information

S1 Table. Independent Ethics Committee and Institutional Review Board Details

| Primary Investigator/ Address | IEC Name & Address |
| --- | --- |
| William J. Steinbach,M.D., Ph.D. Duke University Health System  Research Drive Rm 00541 Blue Zone, Duke South 2424 Erwin Road Room 405 Hock Plaza  Division of Pediatric Infectious Diseases Institutional Review Board  Durham, NC, 27710 Durham, NC 27705  USA | Duke University Health System  2424 Erwin Road Room 405 Hock Plaza Institutional Review Board  Durham, NC 27705 USA |
| Theoklis Zaoutis, M.D.  3535 Market Street, Suite 1527 CHOP North Pediatric Infectious Diseases  Philadelphia, PA, 19104 USA | The Children's Hospital of Philadelphia  3535 Market Street, Suite 1527 CHOP North 3535 Market Street, Suite 1200  Pediatric Infectious Diseases Internal Review Board  Philadelphia, PA, 19104 Philadelphia, PA 19104  USA |
| Reuven Schore, M.D. Children's National Medical Center  111 Michigan Avenue NW West Wing 4th Floor 111 Michigan Avenue, NW  Center for Cancer and Blood Disorders Division of Oncology Institution Review Board  Washington, DC, 20010 USA | Children's National Medical Center  111 Michigan Avenue NW West Wing 4th Floor 111 Michigan Avenue, NW  Center for Cancer and Blood Disorders Division of Oncology Institution Review Board  Washington, DC, 20010 Washington, DC 20010 USA |
| Biomedical Research Alliance of New York  269-01 76th Avenue 225 Community Drive Suite 100  New Hyde Park, NY, 11040 Great Neck, NY 11021 USA | Biomedical Research Alliance of New York  269-01 76th Avenue 225 Community Drive Suite 100  New Hyde Park, NY, 11040 Great Neck, NY 11021 USA |
| Andreas H. Groll EK Arztekammer Westfalen-Lippe& Medizinischen Fak  Albert-Schweitzer-Str. 33 Gartenstrasse 210-214  Klinik U. Polikl. F. Kinderheilkunde Muenster 48147 Germany | EK Arztekammer Westfalen-Lippe& Medizinischen Fak  Albert-Schweitzer-Str. 33 Gartenstrasse 210-214  Klinik u. Polikl. f. Kinderheilkunde Muenster 48147 Muenster, 48149 Germany |
| Thomas Lehrnbecher,Prof., DR., Med. EK Arztekammer Westfalen-Lippe& Medizinischen Fak  Theodor-Stern-Kai 7 Gartenstrasse 210-214 Kinder und Jugendmedizin KKJM 32-8 Paediatrische Haematologie und Onkologie Muenster 48147  Frankfurt/Main, 60590 Germany | EK Arztekammer Westfalen-Lippe& Medizinischen Fak  Albert-Schweitzer-Str. 33 Gartenstrasse 210-214  Klinik u. Polikl. f. Kinderheilkunde Muenster 48147 Muenster, |
| Antonio Arrieta,M.D. Children's Hospital of Orange County IRB  455 South Main Street 4th Floor 455 South Main Street  Oncology Research PSF Pulmonary  Orange, CA, 92868 USA | Children's Hospital of Orange County IRB  455 South Main Street 4th Floor 455 South Main Street  Oncology Research PSF Pulmonary  Orange, CA, 92868 USA |
| Frank Berthold,Prof., DR., Med./Thorsten Simon,PD, DR.,  Med. EK Arztekammer Westfalen-Lippe& Medizinischen Fak  Westfalischen Kerpener Str. 62 Gartenstrasse 210-214  Kinderonkologie und haematologie Muenster 48147 Koeln, 50924 Germany | EK Arztekammer Westfalen-Lippe& Medizinischen Fak  Westfalischen  Kerpener Str. 62 Gartenstrasse 210-214  Kinderonkologie und haematologie Muenster 48147  Koeln, 50924 Germany |
| Marc Lebel,M.D. Comite d'Ethique a la Recherche CHU Ste-Justine  3175 chemin Cote-Ste-Catherine 3175 chemin Cote Ste-Catherine  Montreal, QC, H3T 1C5 Room B-116  Canada | Comite d'Ethique a la Recherche CHU Ste-Justine  3175 chemin Cote-Ste-Catherine 3175 chemin Cote Ste-Catherine  Montreal, QC, H3T 1C5 Room B-116  Canada Montreal, QC H3T 1C5 |
| Lillian Sung The Hospital for Sick Children  555 University Ave. 555 University Avenue Room 5256B, Black Wing  Toronto, ON, M5G 1X8 Toronto, ON M5G 1X8 Canada | The Hospital for Sick Children  555 University Ave. 555 University Avenue Room 5256B, Black Wing  Toronto, ON, M5G 1X8 Toronto, ON M5G 1X8  Canada |
| Elio Castagnola,M.D. Ospedale Pediatrico G. Gaslini  Largo G. Gaslini, 5 Largo G. Gaslini, 5  U.O. Malattie Infettive Comitato Etico  Genova, 16147 Genova 16147  Italy | Ospedale Pediatrico G. Gaslini  Largo G. Gaslini, 5 Largo G. Gaslini, 5 U.O. Malattie Infettive Comitato Etico, Genova, 16147 Genova 16147  Italy |
| Corina E. Gonzalez,M.D. Georgetown University IRB Donna MarieCookmeyer 1  3800 Reservoir Road 3900 Reservoir Road, NW SW104 Med Dent Building  Washington, DC, 20007 Washington, DC 20057 USA  Katherine M Knapp,DR. St. Jude Children's Research Hospital  262 Danny Thomas Place Mail stop 600 262 Danny Thomas Place  Department of Infectious Diseases Institutional Review Board  Memphis, TN, 38105-2794 Memphis, TN 38105 USA | Georgetown University IRB Donna MarieCookmeyer 1  3800 Reservoir Road 3900 Reservoir Road, NW SW104 Med Dent Building  Washington, DC, 20007 Washington, DC 20057, USA  St. Jude Children's Research Hospital  262 Danny Thomas Place Mail stop 600 262 Danny Thomas Place  Department of Infectious Diseases Institutional Review Board  Memphis, TN, 38105-2794 Memphis, TN 38105, USA |
| Edythe Albano,M.D. Western Institutional Review Board  13123 East 16th Avenue, B115 3535 7th Ave. SW  c/o Debra Schissel Olympia, WA 98502-5010 Aurora, CO, 80045 USA | Western Institutional Review Board  13123 East 16th Avenue, B115 3535 7th Ave. SW  c/o Debra Schissel Olympia, WA 98502-5010  Aurora, CO, 80045 USA  USA Western Institutional Review Board  3535 7th Ave. SW  Olympia, WA 98502-5010 USA |
| C. Michel Zwaan,M.D., Ph.D. CCMO  Dr Molewaterplein 60 Parnassusplein, 5  Rotterdam, 3015 GJ Den Haag 2511 VX  Netherlands | CCMO  Dr Molewaterplein 60 Parnassusplein, 5  Rotterdam, 3015 GJ Den Haag 2511 VX  Netherlands Netherlands  CCMO Parnassusplein 5  Den Haag 2511 VX Netherlands |
| M.D. Van de Wetering,M.D., Ph.D. CCMO  Meibergdreef 9 5 Parnassusplein  Room G1-111 Den Haag 2511VX  Amsterdam, 1105 AZ Netherlands | CCMO  Meibergdreef 9 5 Parnassusplein  Room G1-111 Den Haag 2511VX  Amsterdam, 1105 AZ Netherlands  CCMO Parnassusplein 5  Den Haag 2511 VX Netherlands |
| John S. Bradley, M.D. UCSD Human Research Protection Program  3020 Children's Way MC 5041 9500 Gilman Drive Dept. 0052  San Diego, CA, 92123 Human Subjects Committee USA | UCSD Human Research Protection Program  3020 Children's Way MC 5041 9500 Gilman Drive Dept. 0052  San Diego, CA, 92123 Human Subjects Committee  La Jolla, CA 92093 USA |
| Ingo Mueller EK Arztekammer Westfalen-Lippe& Medizinischen Fak  Westfalischen  Martinist. 52 Gartenstrasse 210-214  Klinik und Poliklinik fur Paediatrische Haematologie und Onkologie  Muenster 48147 Hamburg, 20246 Germany | EK Arztekammer Westfalen-Lippe& Medizinischen Fak  Westfalischen  Martinist. 52 Gartenstrasse 210-214  Klinik und Poliklinik fur Paediatrische Haematologie und  Onkologie Muenster 48147 Hamburg, 20246 Germany |
| Friedhelm Schuster EK Arztekammer Westfalen-Lippe& Medizinischen Fak  Westfalischen  Moorenstrasse 5 Gartenstrasse 210-214  Klinik f. Kinder-Onkol. -Haemat. u. -Immunol. Muenster 48147  Duesseldorf, 40225 Germany | EK Arztekammer Westfalen-Lippe& Medizinischen Fak  Westfalischen  Moorenstrasse 5 Gartenstrasse 210-214  Klinik f. Kinder-Onkol. -Haemat. u. -Immunol. Muenster 48147  Duesseldorf, 40225 Germany |
| Michaela Doering EK Arztekammer Westfalen-Lippe& Medizinischen Fak  Westfalischen  Hoppe-Seyler-Str. 1 Gartenstrasse 210-214  Abteilung Haematologie/Onkologie. Ebene CO2, Raum 301 Muenster 48147  Tuebingen, 72076 Germany | EK Arztekammer Westfalen-Lippe& Medizinischen Fak  Westfalischen  Hoppe-Seyler-Str. 1 Gartenstrasse 210-214  Abteilung Haematologie/Onkologie. Ebene CO2, Raum 301 Muenster 48147  Tuebingen, 72076 Germany |
| Jeffrey L. Blumer,M.D., Ph.D. ProMedica Health System Institutional Review Board  2142 N. Cove Blvd. 2142 N. Cove Blvd Conrad Jobst Tower  Toledo, OH, 43606 USA | ProMedica Health System Institutional Review Board  2142 N. Cove Blvd. 2142 N. Cove Blvd Conrad Jobst Tower  Toledo, OH, 43606 USA |
| Philip Toltzis,M.D./David Speicher,M.D. Office of Institutional Review (UHCMC IRB), University Donna MarieCookmeyer 1 Hospitals Case Medical Center, Case Western  11100 Euclid Ave 11100 Euclid Avenue Lakeside Room 1400  Room # 3131 IRB, For Human Investigation  Cleveland, OH, 44106 USA | Office of Institutional Review (UHCMC IRB), University Donna MarieCookmeyer 1  Hospitals Case Medical Center, Case Western  11100 Euclid Ave 11100 Euclid Avenue Lakeside Room 1400  Room # 3131 IRB, For Human Investigation  Cleveland, OH, 44106 USA |
| Dawn Nolt,M.D. Oregon Health Science University  3181 SW Sam Jackson Park Rd 3181 SW Jackson Park Road L106-RI  Portland, OR, 97201 Research Integrity Office USA | Oregon Health Science University  3181 SW Sam Jackson Park Rd 3181 SW Jackson Park Road L106-RI  Portland, OR, 97201 Research Integrity Office  USA Portland, OR 97239 USA |
| Christine Duncan,M.D. Dana Farber Cancer Institute Institutional Review Board Off. for Human Rsrch Studies  450 Brookline Avenue 450 Brookline Ave, Suite 5, Flr 229  Boston, MA, 02215 USA | Dana Farber Cancer Institute Institutional Review Board  Off. for Human Rsrch Studies  450 Brookline Ave, Suite 5, Flr 229  Boston, MA 02215 USA |
| Jessica Pollard,M.D./Phoenix Ho,M.D. Seattle Childrens Hospital Research Foundation - IRB  4800 Sand Point Way NE PO Box 5371  Seattle Childrens CW8-5A  Seattle, WA, 98105 Seattle, WA 98145  USA | Seattle Childrens Hospital Research Foundation - IRB  4800 Sand Point Way NE PO Box 5371  Seattle Childrens CW8-5A  Seattle, WA, 98105 Seattle, WA 98145 USA |
| William B. Slayton  1600 SW Archer Rd  4th Floor, Rm. 4179  Gainesville, FL, 32610 USA | University of Florida IRB  1600 SW Archer Road  Division of Infectious Disease  Gainesville, FL 32610 USA |
| Jennifer Welch,DR./Cindy Schwartz,M.D. LifeSpan Institutional Review Board  Dudley Street Pediatric Hematology/Oncology RI Hosp 593 Eddy Street  Multi-Phasic Bldg, Rm117 Research Protection Office, Office of Research  Administration  Providence, RI, 02903 Providence, RI 02903 USA | LifeSpan Institutional Review Board  Dudley Street Pediatric Hematology/Oncology RI Hosp 593 Eddy Street  Multi-Phasic Bldg, Rm117 Research Protection Office, Office of Research Administration  Providence, RI, 02903 Providence, RI 02903 USA |
| Helen V. Kosmidis,M.D./Apostolos Pourtsidis,DR., Med. National Ethics Committee  Oncology Department, "Elpida" building, 2nd floor 284, Mesogeion Avenue  2 Levadias Str., Ampelokipi Holargos 15562 Athens, 115 27 Greece | National Ethics Committee  Oncology Department, "Elpida" building, 2nd floor 284, Mesogeion Avenue  2 Levadias Str., Ampelokipi Holargos 15562  Athens, 115 27 Greece |
