# Supplementary material for: A non-randomized trial to assess the safety, tolerability, and pharmacokinetics of posaconazole oral suspension in immunocompromised children with neutropenia
Source: PLoS One. 2019 Mar 26;14(3):e0212837. doi: 10.1371/journal.pone.0212837 (PMC6435162; doi:10.1371/journal.pone.0212837)
Supplement: S1 File — (DOCX) [file pone.0212837.s002.docx]

# Supplementary Information

**S1.** Criteria for subjects considered to be Day 7 PK Evaluable

To be PK evaluable at Day 7 (primary PK analysis) the subject was required to meet the following criteria:

1. Subject should have at least 7 sequential and complete days of POS dosing, and at least 90% of each individual dose (i.e., subject didn't refuse to take half of the POS dose).

2. The Day 7 & 8 trough samples must be collected before the next POS dose was taken.

3. Subject must have a minimum of 3 PK samples including 1 pre-dose sample from the full PK profile drawn on Day 7. Post-dose samples (i.e., 3-hour, 5-hour, and 8-hour* samples) must be obtained prior to the next scheduled dose.

4. On Days 1, 3, 5, and 7 the subject was administered their POS dose within 3 hours of the scheduled time for BID dosing and within 4-13 hours of the previous dose of POS for TID dosing.

5. The date and time of PK samples and POS doses were recorded completely.

6. Subject had not taken any prohibited concomitant medications affecting the PK of POS while on treatment: phenytoin, rifabutin, cimetidine, efavirenz, atazanavir, fosamprenavir, ritonavir, omeprazole, or other proton-pump inhibitors or non-study POS.

7. Subject had received the correct dose by weight or within 10% of the correct dose.

8. Trough samples for Days 7 and 8 were taken within 9-15 hours after the previous dose administration for BID dosing, within 7-12 hours (+/-15 minutes) after the previous dose administration for TID dosing, and prior to the next dose.

9. Subject must have a pre-Day 1 POS concentration of <5% of their C_max_ value.

Note: * For TID dosing, if the 8-hour sample could not be obtained due to dosing schedule, then

the sample should have been obtained after the 5-hour sample and prior to the next dose. In case the 8-hour sample was not obtained prior to the next scheduled dose, the 8-hour sample was excluded from the PK analysis.
