# Supplementary material for: A non-randomized trial to assess the safety, tolerability, and pharmacokinetics of posaconazole oral suspension in immunocompromised children with neutropenia
Source: PLoS One. 2019 Mar 26;14(3):e0212837. doi: 10.1371/journal.pone.0212837 (PMC6435162; doi:10.1371/journal.pone.0212837)
Supplement: S3 File — (PDF) [file pone.0212837.s004.pdf]

1.0 TITLE PAGE

20 AUG 2007 – FINAL
23 JAN 2008 - AMENDMENT #1
17 NOV 2008 - AMENDMENT #2
13 OCT 2010 - AMENDMENT #3
05 APR 2013 – AMENDMENT #4

SPONSOR:	Merck Sharp & Dohme Corp., a Subsidiary of Merck & Co., Inc. (hereafter referred to as the Sponsor or Merck) One Merck Drive P.O. Box 100 Whitehouse Station, NJ 08889-0100, U.S.A.
STUDY TITLE:	Phase 1B Study of the Safety, Tolerance, and Pharmacokinetics of Oral Posaconazole in Immunocompromised Children With Neutropenia
PROTOCOL NO.:	P03579
IND NO.:	51,662
SCH NO.:	56592
EUDRACT NO.:	2007-004645-15
TRIAL PROJECT PHYSICIAN:	PPD

SUMMARY OF CHANGES

PRIMARY REASONS FOR THIS AMENDMENT:

Section Numbers	Section Titles	Description of Changes
2.0;<br>2.1.1;<br>2.1.2;<br>5.2.2;<br>7.1;<br>7.1.1.3;<br>7.3;<br>7.4.1;<br>7.4.1.1;<br>8.0;<br>8.3.1;<br>8.5;<br>8.6	Protocol Synopsis,<br>Study Diagram for Dosing Schedule;<br>Study Diagram for Dose Groups and Pharmacokinetics;<br>Study Rationale;<br>Design of the Study/Methodology;<br>Treatment Day(s);<br>Study Population;<br>Study Treatments;<br>Treatments Administered;<br>Statistical and Analytical Plans;<br>Pharmacokinetic Parameters;<br>Determination of Sample	Several key changes have been made based on a review of the currently available clinical PK and safety pediatric data from this ongoing study. The data suggest that BID dosing regimens fail to achieve the PK exposure target for this study (i.e., ~90% of subjects with POS steady-state C_{avg} in the range of 500 ng/mL to 2500 ng/mL) (see Appendix 4). Data from adult studies suggest that dividing the daily dose TID may increase the POS exposure level. As a result, the key changes in this amendment include the following:<br>• Enrollment in Age Group 1 Dose Group 2 (7 to <18 years, receiving 18 mg/kg/day divided BID) was stopped early based on available Dose Group 2 PK data, so that Age Group 1 subjects can advance to Dose Group 3 at 18 mg/kg/day divided TID.<br>• For Age Group 3 Dose Group 1 (3 months to <2 years), the dosing schedule was changed to 12 mg/kg/day divided TID from 12 mg/kg/day divided BID. Similarly, for Age Group 3 Dose Group 2, the dosing schedule was changed to 18 mg/kg/day divided TID from 18 mg/kg/day divided BID.<br>• For Age Group 3 (3 months to <2 years), Dose Group 3, was deleted. Age Group 3 will have two dose groups only.<br>• An additional PK and safety analysis of all available TID data across age groups was added after Age Group 2 Dose Group 3 (7 to <18 years, receiving 18 mg/kg/day divided TID) completes enrollment of 12 PK-evaluable subjects. This will be done to evaluate the probability of success that TID dosing will meet the POS exposure target.<br>The statistical methods section was updated to reflect that the target steady-state

	Size/Power/Level of Significance;<br>Interim Analysis	C_{avg} is greater than or equal to 500 ng/mL.<br><br>The sample size description was edited to reflect the updates to the study design.
2.0;<br>2.2;<br>7.1;<br>7.1.1.3;<br>7.1.2;<br>7.5;<br>7.6	Protocol Synopsis;<br>Study Flow Chart;<br>Design of the Study/Methodology;<br>Treatment Day(s);<br>Plasma/Serum Samples;<br>Blood Sampling;<br>Study Procedures	For Age Group 3 subjects (3 months to <2 years of age) weighing <6.5 kg, there will be no PK samples drawn on Day 1. This change was made to minimize blood sampling in the youngest age group subjects, as per a regulatory commitment and the Committee for Medicinal Products for Human Use (CHMP) 2008 guidance document, “Ethical Consideration for Clinical Trials on Medicinal Products Conducted with the Paediatric Population.”

ADDITIONAL CHANGES FOR THIS AMENDMENT:

Section Numbers	Section Titles	Description of Changes
Title page		The DOC ID row was deleted because this is no longer applicable. The study director's and the Sponsor's names were updated. The confidentiality statement was updated.
2.0	Protocol Synopsis	The number of study centers was expanded to 30. The duration of the study was expanded to 80 months.
2.0;<br>5.1.3.3	Protocol Synopsis;<br>Pediatric Experience	The number of subjects less than 18 years of age that received POS was updated based on the information from most recent Investigator's Brochure.
2.0	Protocol Synopsis	Text in the methodology section was added to show that subjects that are recipients of allogeneic HSCT during the pre-engraftment period (neutropenia period) are eligible to be included in the study population. This change was already made to Section 7.3.1 (Subject Inclusion Criteria) in the previous amendment.<br>The phrase "PK-evaluable" was added to indicate that the target of 12 subjects enrolled in each age group is to be those subjects that are PK-evaluable.
2.0;<br>7.3.2	Protocol Synopsis;<br>Subject Exclusion Criteria	A note was added to Exclusion Criterion Number 11 to explain that if a medication received a regulatory approval for use in adults, then the medication would be considered to have received a regulatory approval for the purpose of the criterion. A reminder was included to indicate that any medication received by eligible subjects must also be aligned with the protocol guidance for prohibited medications.
2.0;<br>5.2.3;	Protocol Synopsis;<br>Study and Dose Rationale;	For clarification, the word "external" was used to describe the Data Monitoring Committee throughout the protocol.

7.1	Design of the Study/Methodology	
2.0;<br>7.4.1.1	Protocol Synopsis; Treatments Administered	For clarification in these sections, the phrase "oral suspension" was added after "POS" in the name of the test product.
2.0;<br>7.7.1	Protocol Synopsis;<br>Pharmacokinetics	These sections were updated to note that the C_{avg} will be used to compare the pediatric PK with the adult PK. The text that states steady-state trough samples will be compared against adult data was deleted. The text that referred to exploring the use of a population PK approach to estimate AUC was deleted because this approach is not necessary for extensive PK sampling schemes.
2.0;<br>8.4.1	Protocol Synopsis;<br>Adverse Events	In the statistical methods and the statistical and analytical plans sections, the text for the AEs noted during the study was updated to reflect that the number of subjects reporting each AE will be presented by dose and age group (not treatment group).
2.2	Study Flow Chart	A new footnote b was added as a reminder that two baseline ECGs must be performed at least 5 minutes apart at the Baseline Visit for purposes of protocol eligibility. The footnotes in the study flow chart after this new additional footnote were re-lettered accordingly.
2.2;<br>7.6	Study Flow Chart;<br>Study Procedures	The footnote in the study flow chart that states "During periods of neutropenia (ANC ≤500/mm^3)" was deleted from the Baseline Visit (Day -1) for the absolute neutrophil count row for clarification. In the study procedures for absolute neutrophil count, the phrase "during periods of neutropenia (ANC ≤500/mm^3)" was deleted. These changes were made for clarification because an absolute neutrophil count is to be performed for all subjects at the Baseline Visit.
5.1.1;<br>5.1.3.1;<br>5.1.3.3;	Class or Type of Drug Being Studied/Description of Drug; Pharmacokinetics;	References to sections in the Investigator's Brochure were updated to the corresponding sections in the most recent version of the brochure.

11	Pediatric Experience; References	
5.1.3.3;<br>Appendix 4	Pediatric Experience;<br>Summary of Safety Data and POS Preliminary Pharmacokinetic from P03579	A summary of the safety and preliminary PK results from the Age Groups 1 and 2 from the first two dose groups of P03579 was added.
7.1	Design of the Study/Methodology	The word “consecutive” was added to clarify that the doses are to be consecutive doses when considering whether a replacement subject may be entered. This is consistent with other sections of the protocol.
7.4.1.1	Treatments Administered	A statement was added providing the results of a dose recovery study by the Sponsor which indicated that oral dosing syringes of six different sizes may be used for dosing.
7.4.1.1	Treatments Administered	A recommendation was included that POS oral suspension may be administered via enteral tubes (e.g., gastric or naso-gastric tubes) if these feeding tubes are inserted for nutritional support in children who do not have any contraindication for enteral feeding. The recommendation was based on results of stability and compatibility tests that support the administration of POS oral suspension via naso-gastric or gastric tubes made of PVC, silicone, or polyurethane.
7.4.1.2	Timing of Dose for Each Subject	The section was modified to note that if for any reason the timing of the medication needs to be adjusted after the first dose, the dose time may be adjusted corresponding timeframes were given for BID and TID dosing.
7.4.1.5.3	Labeling, Storage and Dispensing	An update was made to the instructions on the label of each POS bottle.
7.4.1.5.5	Drug Accountability	Instructions were added to allow the investigator or designee to destroy unused or partially used study drug, provided that the investigator retains sufficient records of

		the destruction of the study drug.
7.4.2.1;<br>7.4.2.1.1;<br>7.4.2.1.2	Prior and Concomitant Medications;<br>Medications Prohibited Prior to Study Drug Administration and During the Study Treatment Phase;<br>Medications Allowed During the Study	Under antiretroviral agents, the sentence that stated that POS was contra-indicated in patients taking efavirenz was updated to state that coadministration of POS with atazanavir, efavirenz, fosamprenavir, or ritonavir is not permitted during treatment phase of the study.<br>Atazanavir, fosamprenavir, and ritonavir were added to the row with efavirenz in Table 3, "Prohibited Medications Prior to Study Drug Administration and During the Study Treatment Phase", and the washout period for these is 24 hours.<br>Atazanavir was deleted from the list of examples of antiretroviral therapies that are allowed during the study.
7.4.2.1.1;<br>7.4.2.1.2	Medications Prohibited Prior to Study Drug Administration and During the Study Treatment Phase;<br>Medications Allowed During the Study	"Antiarrhythmics: procainamide, sotalol, dofetilide, and any approved agent known to prolong QTc or reported to cause TdP" were removed from Table 3 (Prohibited Medications Prior to Study Drug Administration and During the Study Treatment Phase) and added to medications allowed during the study with clinical and/or QTc monitoring recommended. If any of these agents are used concurrently with POS, then it is recommended that there be clinical and/or QTc monitoring. This is indicated in the protocol.
7.4.2.1.1;<br>7.4.2.1.2	Medications Prohibited Prior to Study Drug Administration and During the Study Treatment Phase	Cimetidine was added to Table 3 (Prohibited Medications Prior to Study Drug Administration and During the Study Treatment Phase) to the medications known to lower the serum concentration/efficacy of azole antifungals. Omeprazole or other proton-pump inhibitors or cimetidine for stress ulcer prophylaxis or treatment row was deleted from the table, as there is no washout period for these medications.
7.4.2.1.1	Medications Prohibited Prior to Study Drug	Table 3 (Prohibited Medications Prior to Study Drug Administration and During the Study Treatment Phase) was further updated, as follows:

	Administration and During the Study Treatment Phase	• Quinidine was added to the 3rd row of the table, to the medications that are known to interact with azoles and may lead to life-threatening side effects. The washout period for quinidine is now 24 hours.<br>• The separate row for quinidine was deleted. The anthracycline row of the table was edited to be more generally descriptive.<br>• The row on lipid formulations of anthracycline chemotherapy in the table was deleted.
7.4.2.2.4	Caffeine	There are no diet restrictions with regard to caffeine- or xanthine-containing beverages or drinks. Hence, this section was deleted.
7.7.2.3;<br>Appendix 2	Reporting of Investigational Medicinal Product Quality Complaints;<br>Posaconazole Oral Administration	The name of the form used to report any defect or possible defect in the investigational medicinal product to the Sponsor was updated.
7.7.2.4	Data Monitoring Committee	This new subsection was added for information to indicate that the safety of the subjects is monitored on an ongoing basis by an external Data Monitoring Committee.
11	References	Reference list was updated to include the Sponsor's technical report on the POS oral suspension pediatric device dose recovery study.
Appendix 2	Posaconazole Oral Administration	The section was updated to note that weighing of the bottles is recommended but not required. If the investigator site is unable to follow this compliance/reconciliation check, then the site will need to discuss an alternate method with the site's local Sponsor monitor. The alternate method needs to be approved and documented in the site's materials and on the final study drug compliance/reconciliation documentation.

2.0 SYNOPSIS

<table>
<tr><td colspan="2">Title of Study: Phase 1B Study of the Safety, Tolerance, and Pharmacokinetics of Oral Posaconazole in Immunocompromised Children With Neutropenia (Protocol No. P03579)</td></tr>
<tr><td colspan="2">EUDRACT Number: 2007-004645-15</td></tr>
<tr><td colspan="2">Study Centers: Approximately 30 centers in the US, Canada, and EU</td></tr>
<tr><td>Duration of Study: Approximately 80 months</td><td>Clinical Phase: 1B</td></tr>
<tr><td colspan="2">Objectives

Primary Objective: The primary objective of this study is to evaluate the pharmacokinetics (PK) of posaconazole (POS) administered orally at three dosage levels to immunocompromised children aged 3 months to <18 years with neutropenia or expected neutropenia.

Secondary Objective: The secondary objective is to evaluate the safety and tolerability of POS administered orally at three dosage levels to immunocompromised children with neutropenia or expected neutropenia aged 3 months to <18 years and to compare the exposures to POS in pediatric subjects to those from an adult population with similar underlying conditions.</td></tr>
<tr><td colspan="2">Rationale: Invasive fungal infections (IFIs) are a leading cause of infectious disease morbidity and mortality in immunocompromised patients. Like adults, the pediatric patient population at risk for developing IFIs is similar, primarily due to neutropenia and T-cell dysfunction, which includes, but is not limited to allogeneic stem cell transplant (SCT) recipients, patients with acute leukemias, myelodysplasia, severe aplastic anemia, and advanced stage non-Hodgkin’s lymphoma. Incidence of IFIs in pediatric patients with cancer ranges from 5% to 20%, but this incidence is higher in certain subgroups such as recipients of stem cell transplants (up to 16%), patients with acute leukemia (10% to 20%), or acute myelogenous leukemia (9%), for example. POS is a potent triazole antifungal agent with a wide spectrum of activity against both pathogenic yeasts and moulds, and may be useful in pediatric patients who are at risk for developing IFIs. Clinical studies in subjects (≥13 years of age) using POS oral suspension support the efficacy of POS in the treatment of severely immunocompromised patients, either to prevent IFIs (prophylaxis) or for treatment of IFIs. Furthermore, exposure to POS appears to be correlated with efficacy. Clinical failure in the pivotal prophylaxis trial that showed survival benefit in neutropenic subjects administered 600 mg/day appeared to be correlated with the extent of POS exposure (average plasma concentrations at steady state [C_{avg}] = 583 mg/dL). In addition, approximately 80% of the subjects in this study achieved POS concentrations greater than or equal to 285 ng/mL. The mean plasma concentrations in HIV-subjects with oropharyngeal candidiasis (OPC) treated with POS 100 mg/day was 254 ng/mL. In subjects with IFIs after receiving 800 mg/day, POS C_{avg} was 817 ng/mL.

Up to 31 MAR 2012, a total of 226 subjects <18 years of age received treatment with POS. The two youngest subjects exposed to POS were 22 and 23 months old, and were enrolled under special exemption in a compassionate-use protocol (P02095). While the data obtained thus far in pediatric subjects suggest POS has been well tolerated and will provide therapeutic benefit in the proposed patient population, the data for pediatric subjects <18 years of age are limited in number. Additionally, limited PK data are available for pediatric subjects <7 years of age, and no safety or PK data are available for pediatric subjects <2 years of age. Thus, neither the prophylaxis dose nor the treatment dose for Aspergillus and Candidiasis have been established for patients <13 years of age. The treatment dose for OPC has not been established for patients <18 years of age.

The proposed starting dose for this trial, 12 mg/kg/day, was chosen based on previous experience with POS in adult and pediatric subjects ranging in age from 7 years to 17 years. In the treatment study (P00041) all subjects received 800 mg POS per day, or 11 mg/kg/day for a 70 kg adult. The majority of pediatric patients (8 to 17 years of age) received the full adult dosing regimen, within the range of 15 mg/kg/day to 24 mg/kg/day. Despite slight differences in dose, the C_{avg} achieved in adults (844 ng/mL) was similar to the C_{avg} achieved in pediatrics (776 ng/mL). In the prophylaxis studies, subjects received 600 mg/day, or 8.5 mg/kg/day (for a 70 kg adult), which also provided similar exposure in adults (C_{avg} = 578 ng/mL) as in pediatric subjects (13 to 17 years of age; C_{avg} = 694 ng/mL). Given that the systemic exposure to POS was similar between adolescent subjects and adults, and was not influenced by age, weight or body surface area, it was anticipated that the starting dose of 12 mg/kg/day may result in exposures that are similar to those observed in the pediatric and adult populations at this approximate dose, and for which efficacy has already been established in adults. In addition, in healthy</td></tr>
</table>

Title of Study: Phase 1B Study of the Safety, Tolerance, and Pharmacokinetics of Oral Posaconazole in Immunocompromised Children With Neutropenia (Protocol No. P03579)

volunteers and patients, no increase in the incidence or severity of adverse events was observed at higher exposures compared with lower exposures.

Methodology: This will be a nonrandomized, multicenter, open-label, sequential dose-escalation study to evaluate the safety, tolerance, and PK of oral POS, and will be conducted in conformance with Good Clinical

Practices (GCP). Subjects enrolled will be immunocompromised children 3 months to <18 years of age with neutropenia or expected neutropenia (absolute neutrophil count [ANC] ≤500/mm^3). Study drug will be administered following chemotherapy given for a diagnosis of leukemia (newly diagnosed or relapsed), myelodysplastic syndrome, advanced stage non-Hodgkin's lymphoma, high risk neuroblastoma, patients undergoing autologous HSCT, in subjects with severe aplastic anemia, and in subjects that are recipients of allogeneic HSCT during the pre-engraftment period (neutropenia period).

The subject population will be divided into 3 age groups. Age Group 1 will be children 2 years to <7 years, Age Group 2 will be children 7 years to <18 years, and Age Group 3 will be children 3 months to <2 years. The youngest age group, Age Group 3, will not be enrolled until the PK and safety data from the 2 older age groups and first 2 dose groups have been independently reviewed by the Sponsor and the external Data Monitoring Committee.

At each dosage level, a total of 12 PK-evaluable subjects will be enrolled into each age group. The planned maximum sample size is 96 PK-evaluable subjects (12 subjects each in Age Groups 1, 2, and 3 for Dose Groups 1 and 2 plus 12 subjects each in Age Groups 1 and 2 for Dose Group 3). For Age Groups 1 and 2, dose escalation will be done in parallel and independently from each other, and the criteria for dose escalation will be the same. Thus, all subjects per dosage level do not have to be enrolled and assessed before escalation to the next dosage level is made. While the dose escalation criteria are the same for all 3 age groups, the actual doses achieved in each age group may be different. For example, infants may absorb, distribute or metabolize POS differently and may therefore require higher doses than the older age groups.

In Age Groups 1 and 2, the first dose group will receive 12 mg/kg/day of oral POS divided into 2 doses (BID), up to a maximum of 800 mg per day, and the second dose group will receive 18 mg/kg/day of oral POS divided into 2 doses (BID), up to a maximum of 1200 mg per day. The third dose group will receive 18 mg/kg/day orally divided into 3 doses (TID), up to a maximum of 1200 mg/day.

In Age Group 3, the first dose group will receive 12 mg/kg/day of oral POS divided into 3 doses, up to a maximum of 800 mg per day, and the second dose group will receive 18 mg/kg/day of oral POS divided into 3 doses, up to a maximum of 1200 mg per day. There will only be 2 dose groups for Age Group 3.

The PK criteria for dose escalation are based on data from the adult population that found similar exposure following repeated administration of 400 mg BID and 600 mg BID. Doses above 800 mg per day were not found to be beneficial due to the plateau in POS exposure, which may also occur in pediatric subjects.

In subjects weighing >6.5 kg, PK samples will be drawn on Days 1 and 7, immediately prior to oral administration of POS and at approximately 3, 5, 8, and 12 hours from the time of oral administration of the morning dose for BID dosing. If TID dosing occurs, the 12-hour time point will not be drawn, and the 8-hour sample for subjects receiving TID dosing must be taken prior to the next dose. POS C_{min} or trough samples (immediately prior to dosing) will be obtained on Days 3, 5, 8, 14, and on Day 28 or within 24 hours after the last dose of study drug for early discontinuation. For Age Group 3 subjects (3 months to <2 years of age) weighing <6.5 kg, the samples will be taken as described above; however, there will be no PK samples taken on Day 1. In all subjects, both vital signs and laboratory blood tests will be performed throughout the treatment period and at 9 ±2 days of follow-up to assess the safety and tolerance of POS.

Type of Blinding: This is an open-label study with no blinding.

Sample Size (Including Ratio of Subjects Assigned to Treatments)/Power: The planned maximum sample size is 96 PK-evaluable subjects (12 subjects each in Age Groups 1, 2, and 3 for Dose Groups 1 and 2 plus 12 subjects each in Age Groups 1 and 2 for Dose Group 3). With a goal of achieving a C_{avg} in the targeted (500 ng/mL-2500 ng/mL) range in ~90% of subjects, a dose may be considered a success from a statistical point of view if it is observed that at least 10 out of 12 subjects (≥83%) fall within the range for a particular age group. The probability of observing 83% or higher is 89%, 74%, or 56% if the true probability of being within the range is 90%, 85%, or 80%, respectively.

Title of Study: Phase 1B Study of the Safety, Tolerance, and Pharmacokinetics of Oral Posaconazole in Immunocompromised Children With Neutropenia (Protocol No. P03579)

Subject Replacement Strategy: If a subject in a given dosage level and age group receives fewer than 14 consecutive doses of POS for BID dosing or 21 doses for TID dosing, or fails to complete the Day 8 trough PK sample collection for reasons other than drug intolerance, a replacement subject may be entered at that dosage level and age group. Subjects will not be re-enrolled on this study.

Randomization: None.

Stratification: Subjects will be stratified into the following age groups:

Age Group 1: 2 to <7 years of age

Age Group 2: 7 to <18 years of age

Age Group 3: 3 months to <2 years of age

Diagnosis and Criteria for Inclusion: Pediatric subjects with an expected diagnosis or an existing diagnosis of neutropenia will be selected for the study.

Key Inclusion Criteria:

1. Children of either sex and of any race, 3 months to <18 years of age.
2. Subjects' parent or legally authorized representative must be willing to give written informed consent. Assent will be obtained from minors according to institutional practices.
3. Subjects must have documented or anticipated neutropenia (ANC ≤500/mm^3 [0.5 x 10^9/L]) expected to last for at least 7 days and only in the following clinical situations:
   a. Acute leukemia (including new and relapse),
   b. Myelodysplasia,
   c. Severe aplastic anemia,
   d. Autologous HSCT recipients,
   e. High risk neuroblastoma,
   f. Advanced stage non-Hodgkin's lymphoma,
   g. Recipients of allogeneic HSCT during the pre-engraftment period (neutropenia period).
4. Male and female subjects of child-bearing potential must agree to use a medically accepted method of contraception throughout the study and for at least 30 days after stopping the medication, unless they are surgically or medically sterile or agree to abstain from sexual intercourse. Acceptable methods of contraception include **2** of the following:
   a. Condoms (male or female) with spermicide,
   b. Diaphragm or cervical cap (if acceptable according to local standard of care) with spermicide (females),
   c. Hormonal contraceptives or intrauterine device with spermicide (females).

Key Exclusion Criteria:

1. Subjects with proven IFI, as defined by the MSG/EORTC criteria (**Appendix 3**), prior to study entry.
2. Subjects with Grade 3 or Grade 4 nausea and/or vomiting at Screening.
3. Subjects who have received POS within the past 10 days prior to Screening.
4. Subjects receiving prohibited drugs (**Table 3**).
5. Subjects whose laboratory tests are outside normal limits, as follows:
   a. AST or ALT >5 times the upper limit of normal (ULN)
   b. Serum total bilirubin >2.5 x ULN
   c. Calculated creatinine clearance <30 mL/min. Creatinine clearance will be calculated using the following equation:
      Creatinine clearance = k*height (cm)/serum creatinine (mg/dL)
      Where k = 0.45 for a full term baby less than 1 year old; 0.55 for children up to 12 years old; 0.55 for females between the ages of 13 and 21 years; 0.7 for males between the ages of 13 and 21 years.
6. Subjects with QTc prolongation:
   a. Symptomatic QTc prolongation >450 msec (males) or >470 msec (females)
   b. Any QTc prolongation of >500 msec

Title of Study: Phase 1B Study of the Safety, Tolerance, and Pharmacokinetics of Oral Posaconazole in Immunocompromised Children With Neutropenia (Protocol No. P03579)

7. Subjects who are unable to receive study drug enterally.
8. Female subjects who are pregnant, intend to become pregnant during the course of the study, or are breast-feeding.
9. Subjects with a history of anaphylaxis attributed to the azole class of antifungal agents.
10. Subjects with any clinically significant condition or situation, other than the condition being studied that, in the opinion of the investigator, would interfere with the study evaluations or optimal participation in the study, including receiving less than 7 days of POS.
11. Subjects who have already participated in this study or are participating in any Phase 1 clinical study or any study for a medication that has not yet received regulatory approval. **<u>Note</u>*:*** If the medication has received a regulatory approval for use in adults, then the medication would be considered to have received a regulatory approval for the purpose of this criterion. Any medication received by eligible subjects must also be aligned with the protocol guidance for prohibited medications (Table 3).
12. Subjects who are part of the study staff personnel or family members of the study staff personnel.

Test Product, Dose, Mode of Administration:

<u>Age Group 1 (2 to <7 years) and Age Group 2 (7 to <18 years)</u>

Dose Group 1: POS oral suspension 12 mg/kg/day orally divided into 2 doses, up to a maximum of 800 mg per day.

Dose Group 2: POS oral suspension 18 mg/kg/day orally divided into 2 doses, up to a maximum of 1200 mg per day.

Dose Group 3: POS oral suspension 18 mg/kg/day orally divided into 3 doses, up to a maximum of 1200 mg per day.

<u>Age Groups 3 (3 months to <2 years)</u>

Dose Group 1: POS oral suspension 12 mg/kg/day orally divided into 3 doses, up to a maximum of 800 mg per day.

Dose Group 2: POS oral suspension 18 mg/kg/day orally divided into 3 doses, up to a maximum of 1200 mg per day.

There is no Dose Group 3 for Age Group 3.

Reference Therapy, Dose, Mode of Administration: Not applicable.

Duration of Treatment: Subjects will receive study drug for at least 7 days and will continue to receive treatment until recovery from neutropenia (ANC>500/mm^3) or until initiation of standard of care for either empirical antifungal therapy, or for proven, probable, or suspected IFI. Subjects may receive a maximum of 28 days of study drug and the last study visit will be 9 days ±2 days after administration of the last dose of study drug. Survival assessment, if the subject is alive or dead, will be performed any day from Days 60 to 70. The overall study ends when the last remaining subject has completed or has been discontinued from the study.

Criteria for Evaluation

Pharmacokinetics: The POS plasma concentration-time data will be used to estimate the following PK parameters as the data allow:

- C_{min}: POS trough level immediately before a subject receives the dose on the day specified in the protocol.
- C_{max}: Maximum plasma concentration.
- T_{max}: Time of maximum plasma concentration.
- AUC(tf): Area under the plasma concentration versus time curve from time 0 to the time of the final quantifiable sample.
- AUC(τ): Area under the plasma concentration versus time curve during a dosing interval (τ) at the steady state.
- CL/F: Total apparent body clearance at the steady state.

Title of Study: Phase 1B Study of the Safety, Tolerance, and Pharmacokinetics of Oral Posaconazole in Immunocompromised Children With Neutropenia (Protocol No. P03579)

The current blood samples schedule is an optimized schedule based on the adult steady-state PK data. If all blood samples can be collected at steady-state as scheduled, this optimized schedule will allow a calculation of the steady-state AUC by using non-compartmental trapezoidal method.

If more than one blood sample will be available for a patient, the concentration values will be averaged (C_{avg}). In case, all the planned blood samples can be collected from pediatric patients and an AUC can be calculated as described above, the C_{avg} will be calculated both by averaging all the concentrations in each subject and by dividing the AUC with dosing interval.

Safety: Safety will be assessed based on clinical laboratory test results, adverse events, physical examinations, vital signs, and electrocardiogram (ECG) results.

The occurrence of azole-associated toxicity will be evaluated.

Statistical Methods: Single dose and multiple dose plasma concentrations and derived PK parameters for POS will be listed and summarized by dose and age group using descriptive statistics.

The PK parameters, C_{avg} (and C_{max} and AUC if data allow), will be log transformed for statistical analyses. Point estimates and 90% confidence intervals will be provided for each age group and dose level. If the confidence intervals indicate that ages do not produce a statistical difference on a PK parameter, then age groups will be pooled. To assess preliminary dose proportionality, log transformed, dose normalized C_{avg} (and C_{max} and AUC if data allow) will be analyzed using analysis of variance (ANOVA) extracting the effect due to treatment. Ratio estimates and 90% confidence intervals will be calculated for the differences between doses and ages (if age groups are not pooled). The steady state analysis will be conducted using data from available PK trough values.

Comparison of the exposure from previous experience in adults will be conducted at steady state using graphics. A comparison between adult and pediatric patients with respect to the proportion of patients with steady-state C_{avg} greater than or equal to 500 ng/mL will also be conducted. If appropriate, comparisons between BID and TID dosing with respect to the steady-state C_{avg} and the proportion of patients achieving exposure above 500 ng/mL will be made.

Preliminary analysis will include examining the PK parameters for extreme values by reviewing the standardized ranges of deviations from the expected value derived from the model to see if any value exceeds 3. The impact of any outlier on the results of the analyses will be evaluated.

All AEs noted during the study will be listed. The number of subjects reporting each AE (by dose and age group) and severity will also be presented. Treatment emergent and treatment-related AEs will be tabulated by body system/organ class. The results of hematology and blood chemistry and physical examinations will be listed for each subject. Key toxicities, including hepatotoxicity and nephrotoxicity, will be tabulated by dose and age groups. If 4 or more out of 12 subjects within a dose/age group experience Grade 3 and Grade 4 toxicities in the same organ class which result in definitive discontinuation of study drug treatment and are believed to be potentially cause-related to study drug, then DLT is reached. Assuming the chance of having grade 3/4 toxicity for a subject is 50%, then the probability of having 0, 1, 2, 3 or 4 subjects experiencing grade 3/4 toxicity within a dose/age group is 0%, 0%, 2%, 7% and 19%, respectively.

Day and Visit Structure: See **Section 2.2**, Study Flow Chart for further details.

2.1 Study Design Diagrams

2.1.1 Study Diagram for Dosing Schedule

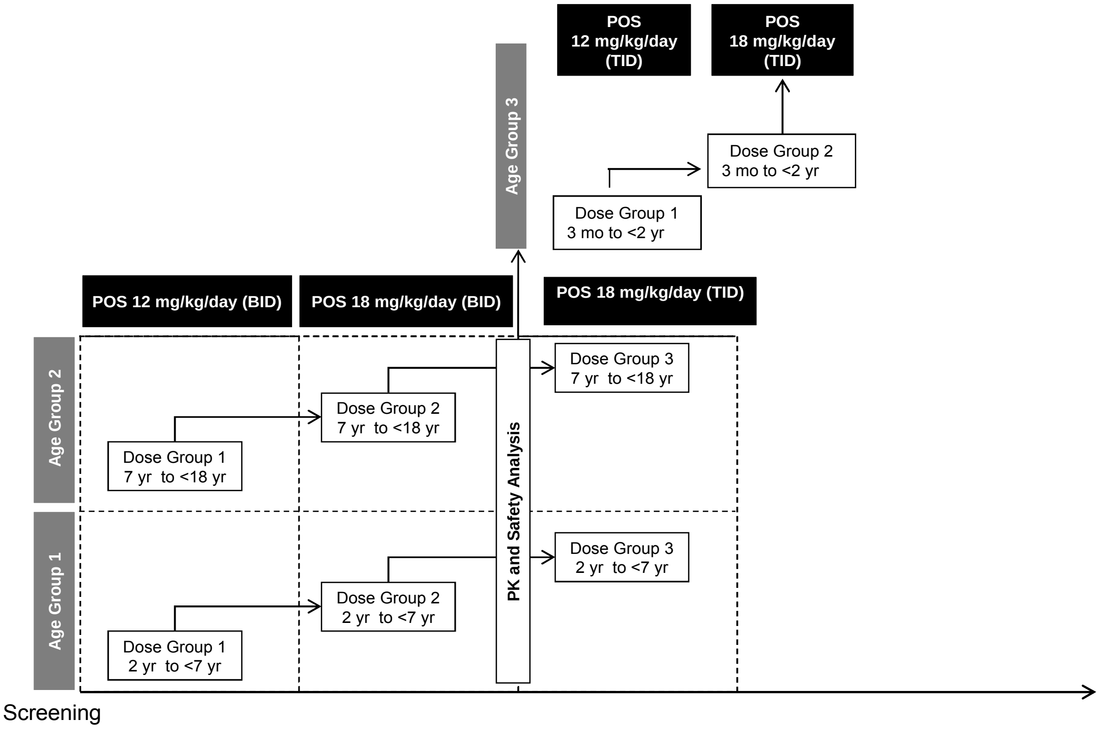

Note: Rising, multiple-dose, sequential-group study.
The arrows represent that all escalation dose groups are to be progressed based on safety and tolerance.
Each age group will escalate independently of the other.
A PK and safety analysis of all available TID data across age groups will be performed after Age Group 2 Dose Group 3 completes enrollment of 12 PK-evaluable subjects.

2.1.2 Study Diagram for Dose Groups and Pharmacokinetics

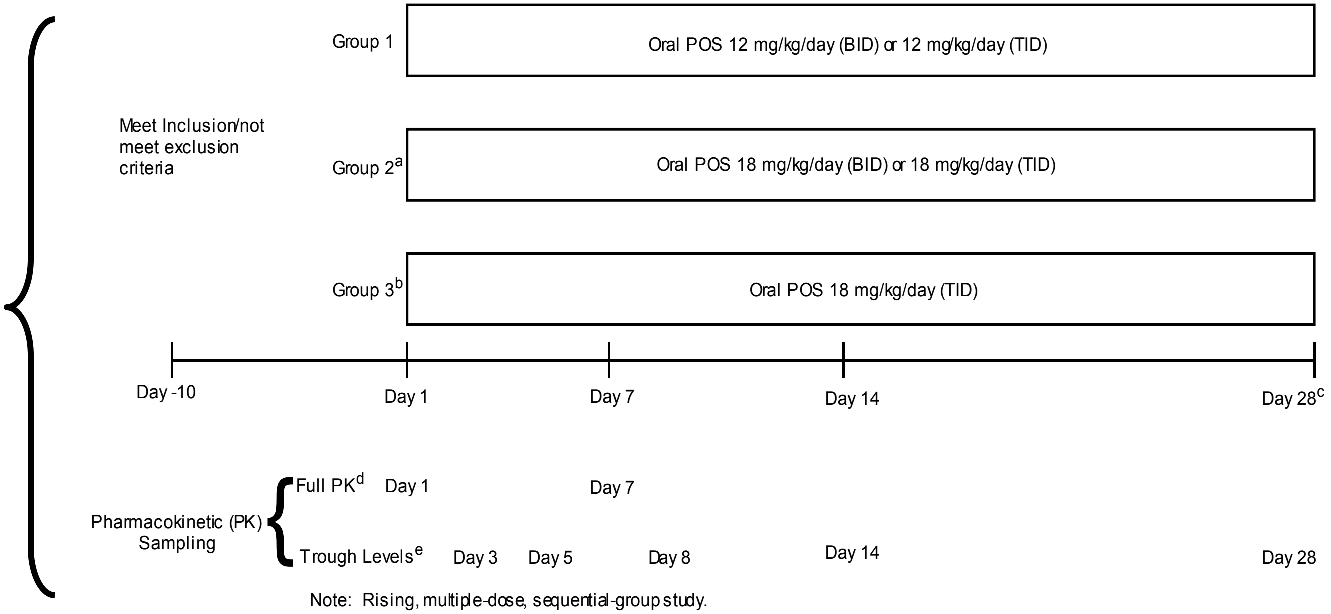

Note: Rising, multiple-dose, sequential-group study.

a: Each of the higher POS dose regimens in the progression will not be administered until the safety and tolerability of the previously administered dose regimens have been determined by the sponsor.
b: Group 3 will run after Groups 1 and 2. Group 3 is for Age Group 1 and Age Group 2 only.
c: All patients may remain on study drug until recovery from neutropenia (ANC>500/mm^3) or for a maximum duration of 28 days.
d: Full PK Sampling time points: predose, and at approximately 3, 5, 8, and 12 hours postdose (12-hour sample will not be obtained if dosing is TID, and the 8-hour sample for subjects receiving TID dosing must be taken prior to the next dose).
For Age Group 3 subjects (3 months to <2 years of age) weighing <6.5 kg, the samples will be taken as described above; however, there will be no PK samples taken on Day 1.
e: Trough levels will be obtained on Days 3, 5, 8, 14, and 28 or within 24 hours after the last day of study drug administration for early discontinuation.

2.2 Study Flow Chart

	Screening	Baseline	Treatment Phase								Follow-up Phase	
			Week 1				Week 2		Week 3	Week 4	Last Study Visit	Survival Assessment
Day Relative to First Dose of Study Drug[a]		Day -1	Day 1	Day 3	Day 5	Day 7	Day 8	Day 14 ± 2 Days	Day 21 ± 2 Days	Day 28/EOT	9 Days ± 2 Days	Day 60 to 70
Obtain Informed Consent/Assent	X											
Screening Number Assignment	X											
Review Inclusion/Exclusion Criteria	X	X										
Demographic Profile	X											
Medical History	X											
Physical Examination	X	X										
Body Weight (kg)		X								X		
Height (cm)	X											
Vital Signs	X	X	X	X	X	X		X	X	X	X	
Electrocardiogram		X[b]		X								
Chest Radiograph		X[c]										
Pregnancy Test (Serum)	X[d]											
Hematology/Serum Chemistry	X		X[e]	X	X	X		X	X	X	X	
Absolute Neutrophil Count		X	X[f]	X[f]	X[f]	X[f]		X[f]	X[f]	X[f]	X[f]	
Pharmacokinetic Samples			X[g,h]			X[h]						
Pharmacokinetic Trough Samples				X[i]	X[i]		X[i]	X[i]		X[i]		
Study Drug Administration[j]			X	X	X	X	X	X	X	X		
Adverse Events[k]	X	X	X	X	X	X	X	X	X	X	X	
Concomitant Medications	X	X	X	X	X	X	X	X	X	X	X	

			Treatment Phase								Follow-up Phase	
	Screening	Baseline	Week 1				Week 2		Week 3	Week 4	Last Study Visit	Survival Assessment
Day Relative to First Dose of Study Drug[a]		Day -1	Day 1	Day 3	Day 5	Day 7	Day 8	Day 14 ± 2 Days	Day 21 ± 2 Days	Day 28/EOT	9 Days ± 2 Days	Day 60 to 70
Dietary Food Intake Assessment			X[l]	X[l]	X[l]	X[l]	X[l]	X[l]	X[l]	X[l]		
Survival Assessment												X[m]

ANC = absolute neutrophil count; C_{min} = minimum observed plasma concentration; ECGs = electrocardiograms; eCRF = electronic case report form; EOT = end of treatment; PK = pharmacokinetic; POS = posaconazole; SAE = serious adverse event.

a Last study visit is 9 days ±2 days after administration of the last dose of study drug.

b Two baseline ECGs must be performed at least 5 minutes apart at Baseline Visit for purposes of protocol eligibility.

c As clinically indicated to evaluate for fungal infection.

d Pregnancy test will be done on females of childbearing potential.

e Prior to first dose.

f During periods of neutropenia (ANC ≤500/mm^3).

g For Age Group 3 subjects (3 months to <2 years of age) weighing <6.5 kg, there will be no PK samples taken on Day 1.

h PK samples will be obtained at 0 hour (predose), and then at approximately 3, 5, 8, and 12 hours after administration of the morning dose. The 12-hour sample must be taken prior to evening dose. If TID dosing is required, the 12-hour sample will not be obtained, and the 8-hour sample for TID must be taken prior to the next dose.

i POS C_{min} or trough samples will be obtained at 0 hour (predose) on Days 3, 5, 8, 14, and 28 or within 24 hours after the last dose of study drug for early discontinuation.

j Study drug will be administered according to the assigned dosing schedule daily on Days 1 through 28, or end of treatment.

k SAEs will be captured beginning with signature of the informed consent until 30 days after the administration of the last dose of study drug.

l A food diary will be completed by the subject and/or the subject's parent(s) or legal guardian and analyzed by a dietitian/knowledgeable healthcare provider with each dose.

m Survival assessment, if the subject is alive or dead, will be performed any day from Days 60 to 70. If the subject dies, the date of death will be recorded.

3.0 TABLE OF CONTENTS

1.0 TITLE PAGE ... 1
SUMMARY OF CHANGES ... 2
2.0 SYNOPSIS ... 9
2.1 Study Design Diagrams ... 14
2.1.1 Study Diagram for Dosing Schedule ... 14
2.1.2 Study Diagram for Dose Groups and Pharmacokinetics ... 15
2.2 Study Flow Chart ... 16
3.0 TABLE OF CONTENTS ... 18
LIST OF TABLES ... 22
LIST OF FIGURES ... 23
LIST OF APPENDICES ... 24
4.0 LIST OF ABBREVIATIONS AND DEFINITIONS OF TERMS ... 25
5.0 INTRODUCTION ... 28
5.1 Background ... 28
5.1.1 Class or Type of Drug Being Studied/Description of Drug ... 28
5.1.2 Preclinical Profile ... 28
5.1.3 Clinical Profile ... 29
5.1.3.1 Pharmacokinetics ... 29
5.1.3.1.1 Pediatric Pharmacokinetics ... 30
5.1.3.2 Safety and Tolerance ... 32
5.1.3.2.1 Comprehensive Clinical Program ... 32
5.1.3.3 Pediatric Experience ... 33
5.2 Rationale ... 34
5.2.1 Project Rationale ... 34
5.2.2 Study Rationale ... 34
5.2.3 Study and Dose Rationale ... 35
6.0 STUDY OBJECTIVES ... 36
6.1 Primary Objective ... 36
6.2 Secondary Objective(s) ... 36
7.0 INVESTIGATIONAL AND ANALYSIS PLAN ... 36
7.1 Design of the Study/Methodology ... 36
7.1.1 Study Plan ... 38
7.1.1.1 Screening ... 38
7.1.1.2 Baseline ... 39
7.1.1.3 Treatment Day(s) ... 39
7.1.1.4 Last Study Visit ... 39

7.1.1.5 Survival Assessment 40
7.1.2 Plasma/Serum Samples 40
7.2 Participation in and Completion of the Study 40
7.3 Study Population 41
7.3.1 Subject Inclusion Criteria 41
7.3.2 Subject Exclusion Criteria 42
7.3.3 Subject Discontinuation Criteria 43
7.3.4 Replacement of Subjects 44
7.4 Treatments 44
7.4.1 Study Treatments 44
7.4.1.1 Treatments Administered 46
7.4.1.2 Timing of Dose for Each Subject 47
7.4.1.3 Method of Treatment Assignment, Randomization, and/or Stratification 47
7.4.1.4 Management of Blinding of Study Treatments 48
7.4.1.5 Investigational Product 48
7.4.1.5.1 Identity of Investigational Product(s) 48
7.4.1.5.2 Source 48
7.4.1.5.3 Labeling, Storage and Dispensing 48
7.4.1.5.4 Packaging 49
7.4.1.5.5 Drug Accountability 49
7.4.2 Other Treatments 50
7.4.2.1 Prior and Concomitant Medications 50
7.4.2.1.1 Medications Prohibited Prior to Study Drug Administration and During the Study Treatment Phase 52
7.4.2.1.2 Medications Allowed During the Study 54
7.4.2.2 Dietary, Tobacco, Alcohol, and Other Restrictions 55
7.4.2.2.1 Diet 55
7.4.2.2.2 Tobacco 55
7.4.2.2.3 Alcohol 55
7.4.2.2.4 Exercise 55
7.4.2.2.5 Other Restrictions 56
7.4.3 Procedures for Monitoring Subject Compliance 56
7.5 Blood Sampling 56
7.6 Study Procedures 56
7.7 Study Analysis Plan 62
7.7.1 Pharmacokinetics 62
7.7.2 Safety 63
7.7.2.1 Specification of Safety Variables 63

7.7.2.2 Assessment and Reporting of Adverse Events 63
7.7.2.2.1 Assessment of Adverse Event Severity and Relationship to Treatment 64
7.7.2.2.2 Monitoring Adverse Events 65
7.7.2.2.3 Known Adverse Events Relating to the Underlying Clinical Condition 67
7.7.2.2.4 Known Potential Toxicities of Study Drug 67
7.7.2.2.5 Definition of Serious Adverse Events 68
7.7.2.2.6 Reporting of Subject Death 69
7.7.2.2.7 Reporting of Pregnancies 69
7.7.2.2.8 Preplanned Hospitalizations or Procedures 69
7.7.2.2.9 Reports of Overdose 70
7.7.2.2.10 Protocol-Specific Exceptions to SAE Reporting to Drug Safety Surveillance 70
7.7.2.3 Reporting of Investigational Medicinal Product Quality Complaints 71
7.7.2.4 Data Monitoring Committee 71
8.0 STATISTICAL AND ANALYTICAL PLANS 71
8.1 Data Sets 72
8.2 Demographic and Other Baseline Characteristics 72
8.3 Pharmacokinetic Analyses 72
8.3.1 Pharmacokinetic Parameters 72
8.4 Safety 73
8.4.1 Adverse Events 73
8.4.2 Clinical Laboratory Tests 73
8.4.3 Vital Signs 73
8.4.4 Physical Examination 73
8.4.5 Electrocardiogram 73
8.4.6 Other Safety 74
8.5 Determination of Sample Size/Power/Level of Significance 74
8.6 Interim Analysis 74
8.7 Other Analyses 74
9.0 ADHERENCE TO ETHICAL, REGULATORY, AND ADMINISTRATIVE CONSIDERATIONS 75
9.1 Ethical Conduct of the Study 75
9.1.1 Independent Ethics Committee or Institutional Review Board 75
9.1.2 Subject Information and Consent 75
9.1.3 Protocol-Related Regulatory and Ethical Considerations/Issues 76
9.2 Reporting to Sponsor 76

9.3 Publications and Other Rights 77
9.3.1 Rights to Publish by the Investigator 77
9.3.2 Use of Proprietary or Confidential Information in a Publication 78
9.3.3 Use of Trial Information in a Publication 78
9.3.4 Authorship of Publications 78
9.4 Shipping of Hazardous or Dangerous Goods 79
9.5 Trial Documents and Records Retention 79
10.0 INVESTIGATORS AND STUDY ADMINISTRATIVE STRUCTURE 80
10.1 Sponsor 80
10.2 Investigators 80
10.2.1 Selecting Investigators 80
10.2.2 Financial Disclosure Requirement 81
10.2.3 Clinical Study Report Coordinating Investigator 81
10.3 Central Organizations 81
11.0 REFERENCES 82

LIST OF TABLES

Table 1 Glossary of Abbreviations and Terms 25
Table 2 A Summary of POS Plasma Concentration in Pediatric Patients Compared to Adults 30
Table 3 Prohibited Medications Prior to Study Drug Administration and During the Study Treatment Phase 53
Table 4 Allowed Medications 54
Table 5 A Summary of POS Plasma Concentrations in Pediatric Subjects ..99
Table 6 Summary of Adverse Events by Category, All Treated Subject 100
Table 7 Summary of Treatment-Emergent Adverse Events, All Treated Subject 101
Table 8 Summary of Treatment-Related Treatment-Emergent Adverse Events, All Treated Subject 113

LIST OF FIGURES

Figure 1 Comparison of Average Posaconazole Plasma Concentration by Age 31
Figure 2 Comparison of Average Posaconazole Plasma Concentration by Weight 31
Figure 3 Comparison of Average Posaconazole Plasma Concentration by Body Surface Area 32

LIST OF APPENDICES

Appendix 1 Specimen Handling and Shipping Instructions 84
Appendix 2 Posaconazole Oral Administration 86
Appendix 3 Defining Opportunistic Invasive Fungal Infections 89
Appendix 4 Summary of Safety and POS Preliminary Pharmacokinetic Data from P03579 98

4.0 LIST OF ABBREVIATIONS AND DEFINITIONS OF TERMS

Table 1 Glossary of Abbreviations and Terms

Protocol No. P03579

AE	Adverse event
ALT	Alanine aminotransferase (SGPT)
AMB	Amphotericin B
ANC	Absolute neutrophil count
ANOVA	Analysis of variance
AST	Aspartate aminotransferase (SGOT)
AUC	Area under the concentration-time curve
AUC(tf)	Area under the plasma concentration versus time curve from time 0 to the time of the final quantifiable sample
AUC(τ)	Area under the plasma concentration versus time curve during a dosing interval (τ) at the steady state
BID	*bis in die*; twice a day
BSA	Body surface area
BUN	Blood urea nitrogen
C_{avg}	Average steady-state plasma concentration
CFR	Code of Federal Regulations
CL/F	Apparent total body clearance
cm	Centimeter
C_{max}	Maximum observed plasma concentration
C_{min}	Minimum observed plasma concentration
CRF	Case Report Form
CSR	Clinical Study Report
CTCAE	Common Terminology Criteria for Adverse Events
CTD	Clinical Trial Directive
CYP	Cytochrome P450
dL	Deciliter
DLT	Dose limiting toxicity
DSS	Drug Safety Surveillance; the Schering Plough department responsible for the receipt, regulatory assessment, and, in the USA, reporting to FDA of all post-marketing adverse events and all serious adverse events from clinical trials
EC	Ethic's Committee
ECG	Electrocardiogram
eCRF	Electronic Case Report Form
EDC	Electronic Data Capture
EORTC	European Organization for Research and Treatment of Cancer

EOT	End of treatment
EU	European Union
F	Female
FDA	Food and Drug Administration, USA
FLU	Fluconazole
GCP	Good Clinical Practice
GMP	Good Manufacturing Practice
GVHD	Graft versus host disease
hCG	Human chorionic gonadotropin
HIV	Human immunodeficiency virus
HMG-CoA	3-hydroxy-3-methylglutaryl coenzyme A
HSCT	Hematopoietic stem cell transplantation
IATA	International Air Transport Association
ICH	International Conference on Harmonisation of Technical Requirements for Registration of Pharmaceuticals for Human Use
ICMJE	International Committee of Medical Journal Editors
IEC	Independent Ethics Committee
IFI	Invasive fungal infection
IMP	Investigational Medicinal Product
IND	Investigational New Drug Application; legal instrument in the USA that allows study of unapproved, investigational new drugs in human subjects
IRB	Institutional Review Board
ITZ	Itraconazole
IV	Intravenous
kg	Kilogram
L	Liter
LDH	Lactate dehydrogenase
M	Male
min	Minute
mg	Milligram
µg	Microgram
MIC	Minimum inhibitory concentration
mL	Milliliter
mm^3	Cubic millimeter
MSG	Mycoses Study Group
MTD	Maximum tolerated dose
NCI	National Cancer Institute
ng	Nanogram
NNRTI	Non-nucleoside reverse transcriptase inhibitors
OPC	Oropharyngeal candidiasis

PDF	Portable Document Format
PK	Pharmacokinetics
POS	Posaconazole
POS oral	Posaconazole aqueous oral suspension
PVC	Polyvinyl chloride
QTc	QT interval corrected for rate
RBC	Red blood cell
RCQ	Research and Development/Commercialization Quality
rIFI	Refractory invasive fungal infection
SAE	Serious adverse event
SCT	Stem cell transplantation
SGOT	Serum glutamic oxaloacetic transaminase (AST)
SGPT	Serum glutamic pyruvic transaminase (ALT)
SOP	Standard Operating Procedure
SPRI	Schering-Plough Research Institute
$t_{1/2}$	Terminal half-life
TAAL	Test Article Accountability Ledger
TASIR	Test Article Summary Inventory Record
TdP	Torsade de pointes
TID	Three times daily
T_{max}	Time to maximum observed plasma concentration
ULN	Upper limit of normal
WBC	White blood cell

5.0 INTRODUCTION

5.1 Background

Posaconazole (POS; SCH 56592) is an oral broad-spectrum antifungal compound discovered by Schering-Plough Research Institute (SPRI). The mechanism of action is selective inhibition of the enzyme lanosterol 14 demethylase (CYP51A1) which is involved in ergosterol biosynthesis in yeasts and moulds. In adults, POS demonstrated superior response and survival in refractory aspergillosis compared to standard therapies.[(1)] Clinical success was also achieved in other difficult-to-treat refractory invasive fungal infections (IFIs), including fusariosis,[(2)] chromoblastomomycosis[(3)] and coccidioidomycosis.[(4)] POS demonstrated superior prophylaxis against IFIs and survival in neutropenic patients compared to fluconazole (FLU) or itraconazole (ITZ),[(5)] and superior prophylaxis and survival in hematopoietic stem cell transplantation (HSCT) recipients with graft versus host disease (GVHD) compared to FLU.[(6)] POS is also indicated as first-line therapy in the treatment of oropharyngeal candidiasis (OPC).[(7)] It has comprehensive in vitro activity equal to or superior to standard therapies as well. It is well tolerated with no dose adjustment required for renally impaired patients, and has limited potential for drug-drug interactions. It is anticipated that POS will provide the same therapeutic benefits in children as has already been demonstrated in adults.

5.1.1 Class or Type of Drug Being Studied/Description of Drug

POS is a broad-spectrum triazole antifungal compound that is being developed for the treatment and prophylaxis of IFIs.

POS oral suspension is provided as a 40 mg/mL aqueous suspension, the components of which are described in the Investigator's Brochure.[(8)] Please refer to Section 8.1 of the Investigator's Brochure for additional information.[(8)]

5.1.2 Preclinical Profile

POS administered orally is supported by an extensive preclinical and clinical program. POS causes several toxicologic effects that occur with other antifungal substances in the azole class, ie, hyperplasia of the adrenal glands (rats, mice, and dogs), phospholipidosis of lung and lymphoid tissues (all species), disseminated intravascular coagulation (dogs only), bone thinning/fractures (rats only), hepatocellular adenomas (mice only), findings secondary to the interruption of steroidogenesis and fetal toxicity (rats and rabbits). POS oral findings not reported with other marketed antifungal agents are neuronal phospholipidosis (only in dogs

with no functional consequence), increased urinary calcium excretion in dogs and rats, histiocytic hyperplasia of lymph nodes of mice, and adrenal cortical and medullary tumors in rats, resulting from interrupted steroidogenesis and altered calcium homeostatis, respectively.

POS oral had no effect on fertility of male or female rats. In studies of embryo and fetal development in the rat at POS oral doses of 3 mg/kg, 9 mg/kg, or 27 mg/kg, the highest dose caused skeletal malformations, while the no-effect dose was 9 mg/kg POS oral. In the rabbit, the no-effect dose of POS oral was 20 mg/kg, while high doses of 40 mg/kg and 80 mg/kg caused an increase in resorptions. A POS oral dose of 80 mg/kg also caused a reduction in female body weight gain and in litter size. No malformations were noted in rabbits. In a peri- and post-natal study, adverse reproductive effects including dystocia, prolonged parturition, reduced F1 mean live litter size and reduced F1 postnatal viability occurred at POS oral doses of 18 mg/kg and above, but not at 6 mg/kg. However, there were no effects on functional battery endpoints in the F1 generation. Embryo resorptions, post-implantation losses, delayed parturition, as well as fetal skeletal malformations and variations are class effects of azole antifungal drugs.

Although maternal or pup exposure to POS was not determined in the peri- and post-natal developmental toxicity study, a lactational transfer study confirmed exposure to POS in pups, and exposure data are available at similar doses from the rat carcinogenicity study that was conducted at 5 (male [M] + female [F]), 10 (M + F), 20 (F) and 30 mg/kg (M). The average steady-state plasma concentration (C_{avg}) achieved in the 10 mg/kg dose group represents exposure multiples of 3.21 and 7.67, for males and females, respectively, relative to the C_{avg} achieved in pediatric subjects administered 800 mg per day POS in divided doses for the treatment of refractory IFIs (P00041; N=12; 8 to 17 years of age). When compared with the C_{avg} achieved in 10 adolescents (13 to 17 years of age) who received 600 mg/day in divided doses for prophylaxis (P01899), the exposure multiples are 3.59 and 8.57 for males and females, respectively. Therefore, the exposure achieved in the animal peri- and post-natal developmental toxicity study was ~3- to ~9-times greater than the exposure achieved in pediatric subjects.

5.1.3 Clinical Profile

5.1.3.1 Pharmacokinetics

The pharmacokinetics (PK) of POS oral is linear following single- and multiple-dose administration up to 800 mg. No further increases in exposure are observed above 800 mg in patients and healthy volunteers. Dividing the total POS daily dose (800 mg) as 400 mg twice daily (BID) results in a 192% higher mean average concentration relative to once-a-day administration in patients (P01893). The area under the plasma concentration-time curve (AUC) of POS is 2.6 times greater when

administered with a nonfat meal or nutritional supplement (14 g fat) and 4 times greater when administered with a high-fat meal (~50 g fat) relative to the fasted state. POS should be administered with food or a nutritional supplement. POS has a large apparent volume of distribution (1774 L) suggesting extensive penetration into the peripheral tissues. Of the circulating metabolites, glucuronide conjugates of POS are major metabolites, with only minor amounts of Phase 1 (CYP450) metabolites. POS is slowly eliminated with a mean half-life (t½) of 35 hours. POS is predominantly excreted in the feces (77% of the radiolabeled dose) with the major component eliminated as parent drug (66% of the radiolabeled dose). Steady state is attained following 7 to 10 days of multiple-dose administration.

Please refer to Section 8.1.1 of the Investigator's Brochure for additional details.[8]

5.1.3.1.1 Pediatric Pharmacokinetics

The POS exposure data obtained thus far from pediatric subjects outside of the current study is summarized in **Table 2**:

Table 2 A Summary of POS Plasma Concentration in Pediatric Patients Compared to Adults

Study	POS Dose	Age Range (yr)	n[a]	Mean C_{avg} (ng/mL)	Range C_{avg} (ng/mL)
C/I98-316	200 mg TID	13-17	2[b]	1302	84.5-2840
		≥18	239	1130	0-4420
P01899	200 mg TID	13-17	8	694	254-1450
		≥18	207	578	89.7-2400
P00041	400 mg BID	8-17	12	776	85.3-2891
		≥18	219	844	0-3710

BID = twice daily; C_{avg} = average plasma concentration; IFI = invasive fungal infection; POS = posaconazole; TID = three times daily.

a Subjects with evaluable samples.

b Includes two pediatric subjects with no IFI; one pediatric subject (7 years of age with an IFI) had plasma concentrations of 524 ng/mL and 857 ng/mL at 3.5 and 5.8 hours postdose on Days 15 and 25, respectively.

Analysis of POS concentration by age, weight and body surface area (BSA) did not show any correlation (**Figure 1**, **Figure 2**, and **Figure 3**).[9]

Limited PK data are available from pediatric subjects less than 7 years of age.

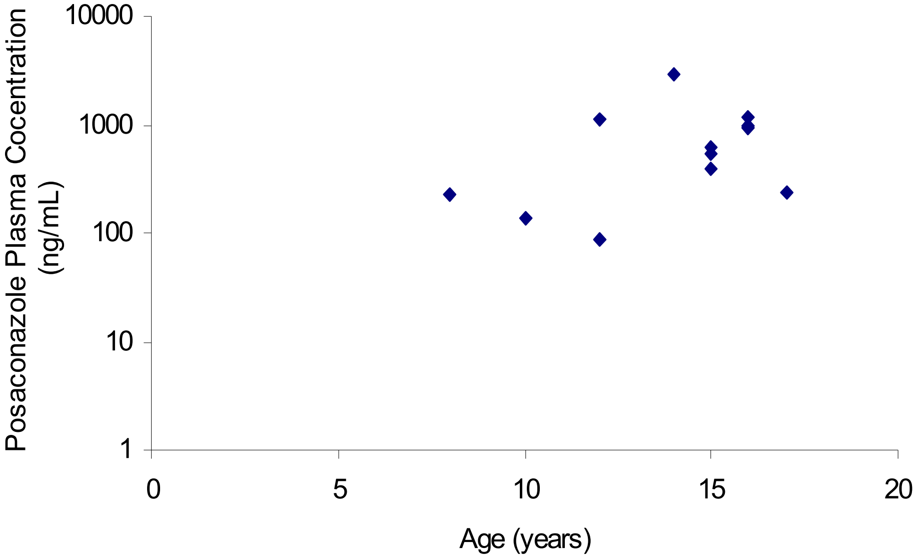

Figure 1 Comparison of Average Posaconazole Plasma Concentration by Age

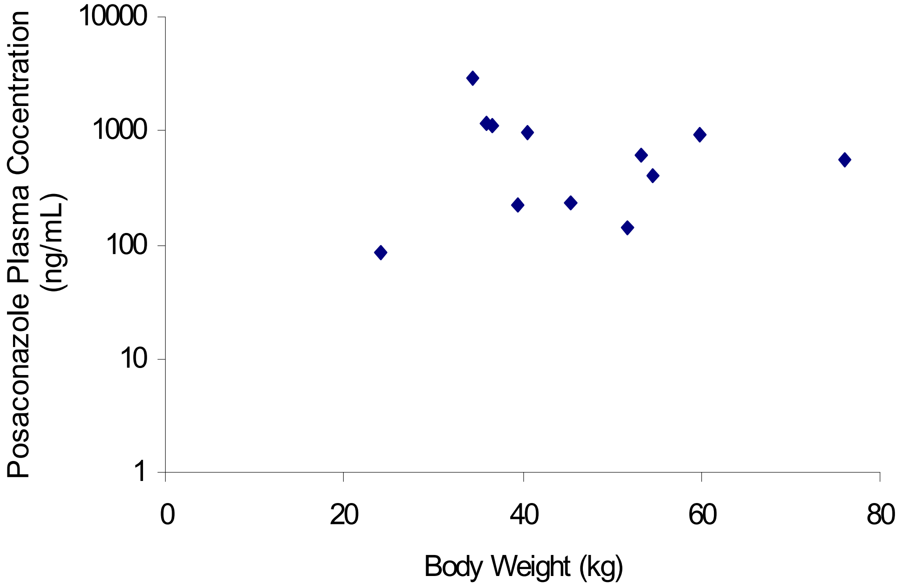

Figure 2 Comparison of Average Posaconazole Plasma Concentration by Weight

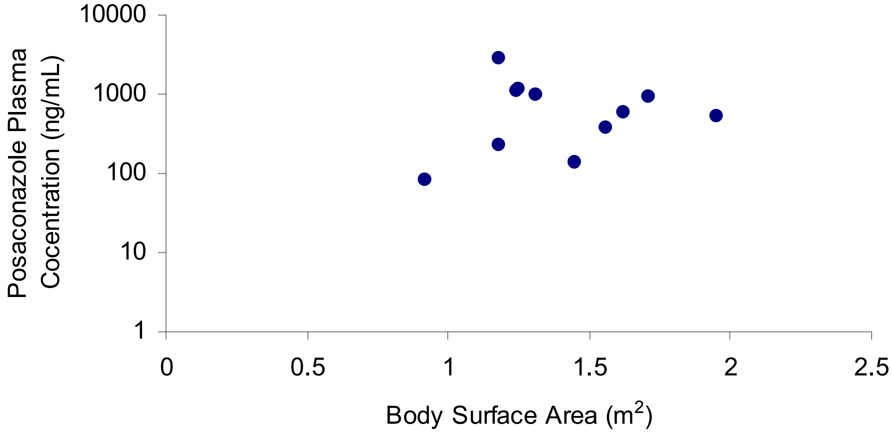

Figure 3 Comparison of Average Posaconazole Plasma Concentration by Body Surface Area

5.1.3.2 Safety and Tolerance

5.1.3.2.1 Comprehensive Clinical Program

Drug-related, adverse reactions were observed in 2,453 subjects in completed clinical trials dosed with POS up to 800 mg/day. One hundred seventy two patients received POS therapy for ≥6 months; 58 of these received POS therapy for ≥12 months. The most frequently reported adverse reactions reported across the whole population of healthy volunteers and patients were nausea (6%) and headache (6%).

Treatment-related serious adverse events reported in 428 patients with IFIs (1% each) included altered concentration of other medicinal products, increased hepatic enzymes, nausea, rash, and vomiting. Treatment-related serious adverse events reported in 605 patients treated with POS for prophylaxis (1% each) included bilirubinemia, increased hepatic enzymes, hepatocellular damage, nausea, and vomiting.

Uncommon and rare treatment related medically significant adverse events reported during clinical trials with POS have included adrenal insufficiency, allergic and/or hypersensitivity reactions.

In clinical trials, there were infrequent cases of hepatic reactions (eg, mild to moderate elevations in alanine aminotransferase [ALT], aspartate aminotransferase [AST], alkaline phosphatase, total bilirubin, and/or clinical hepatitis). The elevations in liver function tests were generally reversible on discontinuation of therapy, and in

some instances these tests normalized without drug interruption. Rarely, more severe hepatic reactions including cholestasis or hepatic failure with fatality were reported in patients with serious underlying medical conditions (eg, hematologic malignancy) during treatment with POS.

In addition, rare cases of torsades de pointes, hemolytic uremic syndrome and thrombotic thrombocytopenic purpura have been reported in patients with significant underlying medical conditions who were treated with POS.

In summary, completed and ongoing laboratory and clinical studies in a wide variety of azole susceptible and refractory fungal infections have demonstrated that POS has a broad spectrum of activity and is safe and well tolerated.

5.1.3.3 Pediatric Experience

Up to 31 MAR 2012, a total of 226 subjects <18 years of age received treatment with POS. The two youngest subjects exposed to POS were 22 and 23 months old, and were enrolled under special exemption in a compassionate-use protocol (P02095). Dose adjustments were recommended for pediatric subjects weighing less than 34 kg. The compassionate-use program (P02095) enrolled 129 pediatric subjects of different races.

A review of the safety and PK data from Age Groups 1 and 2 from the first two dose groups from the current study (P03579) was performed by the external Data Monitoring Committee and the Sponsor. No safety concerns were identified. The POS PK exposure target for the study, ~90% of subjects with POS steady-state C_{avg} in the range of 500 ng/mL to 2500 ng/mL, has not been achieved at either dose level (**Appendix 4**).

For further details regarding the safety of POS in pediatrics, please refer to Section 8.2.2.8 of the Investigator's Brochure[(8)] and **Appendix 4** for the P03579 safety and preliminary PK results.

The comparability of the PK of POS in pediatrics versus adults, and the similarity in the proportion of pediatric subjects reporting serious adverse events (SAEs) or other clinically significant adverse events versus the overall adult patient population by study, indicate that the safety profile in pediatrics appears to be comparable to that in adults.

5.2 Rationale

5.2.1 Project Rationale

IFIs are a leading cause of infectious disease mortality in immunocompromised patients. The pediatric patient population at risk for developing IFIs is similar to adults, and includes, but is not limited to allogeneic stem cell transplant recipients, patients with acute leukemias, myelodysplasia, severe aplastic anemia, and advanced stage non-Hodgkin's lymphoma. Incidence of IFIs in pediatric patients with cancer ranges from 5% to 20%,(10-15) but this incidence is higher in certain subgroups such as recipients of stem cell transplants (up to 16%),(14) patients with acute leukemia (10% to 20%),(5,6) or acute myelogenous leukemia (9%), for example.(11)

POS is a potent triazole antifungal agent with a wide spectrum of activity against both pathogenic yeasts and moulds, and may be useful in pediatric patients who are at risk for developing IFIs. Clinical studies in subjects (≥13 years of age) using POS oral therapy (P00041, P01893, C/I96-421, and P01899) support the efficacy of POS in the treatment of severely immunocompromised patients, either to prevent IFIs (prophylaxis) or as salvage therapy (to treat IFIs that have failed standard antifungal therapy).

In addition, PK, safety and efficacy data for POS in children are sparse. Limited PK data are available for pediatric subjects <7 years of age, and no PK or safety data are available for pediatric subjects <2 years of age. Neither the prophylaxis dose nor the treatment dose for A*spergillus* and *Candidiasis* have been established for patients <13 years of age, and the treatment dose for OPC has not been established for patients <18 years of age.

5.2.2 Study Rationale

Exposures to POS (dosing and levels) associated with clinical efficacy correlated well with levels at or above minimum inhibitory concentrations (MICs) for the pathogens treated or most common pathogens prevented. In a pivotal prophylaxis trial that showed survival benefit, clinical failure appeared to be correlated with the extent of POS exposure. In these studies, study efficacy was correlated with POS plasma concentrations of 500 ng/mL or greater. Thus, targeting same or better POS exposure levels in children of a similar population may confer similar or better protection and efficacy achieved in previous adult studies. In addition, a safety and PK trial of POS is an important step in understanding the safety and pharmacology of POS in children and ultimately, reducing morbidity and mortality related to IFIs in this population.

5.2.3 Study and Dose Rationale

This study is designed as a nonrandomized, multicenter, open-label, sequential dose-escalation study to evaluate the safety, tolerance, and PK of POS. It will include pediatric patients of different age groups to determine if there are differences in exposure to POS affected by differences in drug metabolism related to developmental changes in pediatric patients that may affect the primary pathway for POS metabolism, glucuronidation, and the enzyme (UGT1A4) which is not fully developed until ~2 years of age. Since factors of drug absorption, metabolism, distribution, and elimination are all affected by the age and physiologic maturity of the child, the inclusion of different age groups in this population may help to determine the dose to be used for treatment and prophylaxis of fungal infections in pediatrics.

The proposed starting dose for this trial, 12 mg/kg/day, was chosen based on previous experience with POS in adult and pediatric subjects ranging in age from 7 years to 17 years. In the treatment study (P00041) all subjects received 800 mg POS per day, or 11 mg/kg/day for a 70 kg adult. The majority of pediatric patients (8 years to 17 years of age) received the full adult dosing regimen, within the range of 15 mg/kg/day to 24 mg/kg/day. Despite slight differences in dose, the C_{avg} achieved in adults (844 ng/mL) was similar to the C_{avg} achieved in pediatrics (776 ng/mL). In the prophylaxis studies, subjects received 600 mg day, or 8.5 mg/kg/day for a 70 kg adult, which also provided similar exposure in adults (C_{avg} = 578 ng/mL) as in pediatric subjects (13 years to 17 years of age; C_{avg} = 694 ng/mL). Given that the systemic exposure to POS was similar between pediatric subjects and adults, and was not influenced by age, weight or body surface area, it is anticipated that the starting dose of 12 mg/kg/day may result in exposures that are similar to those observed in the pediatric and adult populations at this approximate dose, and for which efficacy has already been established in adults.

In addition, in healthy volunteers and patients, no increase in the incidence or severity of adverse events was observed at higher exposures compared with lower exposures and the data obtained thus far indicate that the safety profile appears to be similar between adults and children. The vast majority of SAEs observed in children were unlikely to be related to POS, reflecting the serious and advanced nature of the IFI and underlying medical illnesses. Nevertheless, the study design includes an assessment for safety for the first 6 subjects treated at each dose level for each age group employing rapid data collection and centralized analyses. When safety has been assessed for the lowest dose group by the Sponsor and the external Data Monitoring Committee, and a determination has been made that the safety data are adequate to support enrollment into the next higher dosing level, then subjects will be screened and treated. An assessment of differences in exposure between the first two dose groups will be conducted to determine whether TID dosing will be subsequently explored. Subjects in the youngest age group (3 months to <2 years of age) will not be administered POS until the safety and PK data from the two older age groups and first two dose groups have been evaluated

by the Sponsor and the external Data Monitoring Committee. Therefore, key decision points for advancing the dosing to the next dosing group will be based on the combination of frequency and severity of untoward effects.

6.0 STUDY OBJECTIVES

6.1 Primary Objective

The primary objective of this study is to evaluate the PK of POS administered orally at three dosage levels to immunocompromised children with neutropenia or expected neutropenia aged 3 months to <18 years.

6.2 Secondary Objective(s)

The secondary objective of this study is to evaluate the safety and tolerability of POS administered orally at three dosage levels to immunocompromised children with neutropenia or expected neutropenia aged 3 months to <18 years, and to compare the exposures to POS in pediatric subjects to those from an adult population with similar underlying conditions.

7.0 INVESTIGATIONAL AND ANALYSIS PLAN

7.1 Design of the Study/Methodology

This will be a non-randomized, multicenter, open-label, sequential dose-escalation PK study. Subjects enrolled will be immunocompromised children with neutropenia or expected neutropenia between the ages of 3 months to <18 years. Those febrile subjects with proven IFI prior to entry will not be enrolled. For any subject who meets institutional criteria to start standard empirical or pre-emptive antifungal therapy or who has a proven breakthrough fungal infection, POS will be discontinued and pre-emptive or empirical therapy with standard of care will be initiated. A breakthrough fungal infection is defined as microbiological or histological evidence of a deep mycosis developing while a subject is receiving study drug. Subjects must receive one or more doses of POS to be evaluable for toxicity.

If a subject in a given dosage level and age group receives fewer than 14 consecutive doses for BID dosing or 21 consecutive doses for TID dosing, or fails to complete Day 8 trough PK sample collection for reasons other than drug intolerance, a replacement subject may be entered at that dosage level and age

group. PK samples will be drawn on Days 1 and 7 in subjects weighing >6.5 kg; Age Group 3 subjects (3 months to <2 years of age) weighing <6.5 kg will have PK samples drawn on Day 7, but no PK samples will be taken on Day 1. Additionally, in all subjects, POS minimum observed plasma concentration (C_{min}) or trough samples (immediately prior to dosing) will be collected on Days 3, 5, 8, 14, and 28 or within 24 hours after the last dose of study drug administration for early discontinuation. In addition, vital signs and laboratory blood tests will be performed throughout the treatment period and at 9 days ±2 days of follow-up to assess the safety and tolerance of POS.

Subjects will not be re-enrolled onto this study. If a subject is assigned a subject number, but does not receive study drug, the subject number will not be used again. All subjects may remain on study drug for at least 7 days and until recovery from neutropenia (absolute neutrophil count [ANC]>500/mm^3) or for a maximum duration of 28 days.

Oral POS will be administered at one of three dosage levels in the 2 older age groups (Age Group 1 [2 years to <7 years] and Age Group 2 [7 years to <18 years]) and at one of two dosage levels in the youngest age group (Age Group 3 [3 months to <2 years of age]). Study drug will not be administered to the higher dosage group in a given age group until after safety data obtained in at least 6 completed subjects in the lower dosage group in a corresponding age group are reviewed independently by the external Data Monitoring Committee and the Sponsor. Additionally, the subjects in the youngest age group (3 months to <2 years of age) will not be enrolled until all safety and PK data obtained from the two older age groups in the first two dosage levels are reviewed independently by the Sponsor and external Data Monitoring Committee.

For Age Group 1 (2 years to <7 years) and Age Group 2 (7 years to <18 years), the first dose group (Dose Group 1) will receive a total of 12 mg/kg/day of oral POS, divided into 2 daily doses (BID), up to a maximum of 800 mg per day. Within a given age group, escalation to the 18 mg/kg/day can initiate if no more than 2 out of the 6 subjects experience Grade 3 and Grade 4 toxicities in the same organ class which pursuant to protocol item **Section 7.7.2.2** result in definitive discontinuation of study drug treatment and are believed to be potentially cause-related to study drug. The second dose group (Dose Group 2) will receive 18 mg/kg/day of oral POS, divided BID, up to a maximum of 1200 mg per day. For Age Groups 1 and 2, a third dose group (Dose Group 3) will receive 18 mg/kg/day orally divided into 3 doses (TID), up to a maximum of 1200 mg/day. If 4 of 12 subjects experience Grade 3 and Grade 4 toxicities in the same organ class which pursuant to protocol item **Section 7.7.2.2** result in definitive discontinuation of study drug treatment and are believed to be potentially cause-related to study drug at a given dose level, then the maximum tolerated dose (MTD) will have been reached.

For Age Group 3 (3 months to <2 years of age), there will only be two dose groups, Dose Groups 1 and 2; subjects will be administered as 12 mg/kg/day (divided TID) or 18 mg/kg/day (divided TID), in Dose Group 1 and 2, respectively.

The PK criteria are based on data from the adult prophylaxis and treatment studies in which doses above 800 mg per day were not found to be beneficial due to the plateau in POS exposure, which may also occur in pediatric subjects.

The 24 subjects in the youngest age group (Age Group 3 [3 months to <2 years of age]; n=12/dosage group) will receive POS in the same manner using the same criteria for dose escalation and dose-limiting toxicity, and starting with the same total daily dose as the two older age groups. Based on the review of the safety and PK data in the older age groups, the youngest age group will start with a total daily dose divided TID, instead of BID.

All POS doses will be based on the actual body weight at baseline which should be continued through the treatment period.

Escalation to the next higher dosage level will be permitted after mutual agreement by the Sponsor and the external Data Monitoring Committee.

All subjects in a given dosage group do not have to be enrolled and assessed before escalation to the next dosage level. Age Groups 1 and 2 will dose-escalate independently of each other.

7.1.1 Study Plan

7.1.1.1 Screening

Within 10 days prior to treatment, the investigator or qualified designee shall discuss with the parent(s) or legal guardian of each subject, and with each subject as appropriate, the nature of the study, its requirements, and its restrictions. Written informed consent will be obtained from the parent or legal guardian of each subject prior to any study related procedures being performed, and a signed copy will be given. Assent will be obtained from minors according to institutional practices. The inclusion/exclusion criteria will be reviewed, a demographic profile, complete medical history, medication review, physical examination and other assessments delineated in **Section 2.2**, Study Flow Chart, (eg, height determination, vital signs [blood pressure, heart rate and temperature]) will be performed. Blood samples will be collected for evaluation of eligibility criteria and for safety assessments (eg, pregnancy test, clinical safety laboratories, etc). A screening number will be assigned to the subject on completion of the study-specific informed consent. Assessment of SAEs after signature of the Informed Consent will also be recorded.

7.1.1.2 Baseline

The investigator or designee will review the inclusion/exclusion criteria and record medications taken within the last 14 days (chemotherapeutic agents taken within 30 days of the first dose of study drug). A physical examination, vital signs, 12-lead electrocardiogram [ECG], and chest radiograph (as clinically indicated to evaluate for fungal infection) will be performed, as well as an assessment of AEs. ANC will be determined. Weight will be measured.

7.1.1.3 Treatment Day(s)

Subjects will initiate treatment following standard intensive induction or consolidation chemotherapy cycle. Subjects will receive their treatment at approximately the same time every day on Days 1 through 28. Each dose will be administered approximately 12 hours apart for BID regimens and approximately 8 hours apart for TID regimens. Laboratory safety tests (eg, hematology, blood chemistries, and ECG), vital signs, ANC, adverse events, concomitant medications, and dietary food intake assessment will be collected according to the schedule in **Section 2.2**, Study Flow Chart. Meals, including formula or breast milk, will be provided in accordance with the meals section of the protocol **Section 7.4.2.2.1**, Diet. For subjects weighing >6.5 kg, serial PK samples will be obtained from prior to dosing until 12 hours after the morning dose on Days 1 and 7 for BID dosing; age Group 3 subjects (3 months to <2 years of age) weighing <6.5 kg will have PK samples drawn on Day 7, but no PK samples will be taken on Day 1. If TID dosing is required, the 12-hour sample will not be obtained, and the 8-hour sample following TID dosing must be taken prior to the next dose.

In all subjects, POS C_{min} or trough samples (immediately prior to dosing) will be drawn on Days 3, 5, 8, 14 and 28, or within 24 hours after the last dose of study drug administration for early discontinuation.

On the last day, adverse events, concomitant medications, and dietary food intake assessment will be recorded, a blood sample will be collected for laboratory safety tests, and vital signs and body weight will be recorded.

7.1.1.4 Last Study Visit

Follow-up will be conducted 9 days ±2 days after the last day of study drug administration. Adverse events and concomitant medications will be recorded, a

sample will be collected for laboratory safety tests, and vital signs will be recorded. ANC will be determined as clinically indicated.

7.1.1.5 Survival Assessment

Survival assessment, if the subject is alive or dead, will be performed any day from Days 60 to 70. If the subject dies, the date of death will be recorded.

7.1.2 Plasma/Serum Samples

For all subjects >6.5 kg, approximately 25 mL of blood will be collected from each subject:

- 15 samples/subject, 1 mL/sample for PK,
- 9 samples/subject, 1 mL/sample for safety, and
- 1 sample/female of childbearing potential, 1 mL/sample for pregnancy test.

For subjects in Age Group 3 weighing <6.5 kg, approximately 18 mL of blood will be collected from each subject, as no PK samples will be collected on Day 1.

- 9 samples/subject, 1 mL/sample for PK,
- 9 samples/subject, 1 mL/sample for safety.

7.2 Participation in and Completion of the Study

The subject is considered to be enrolled in the study when the subject signs the study specific informed consent. All subjects will have their information entered into the screening log maintained by the study site, and will have information (demographics and reason for screening failure) entered into the eCRF.

The subject is considered to have completed the study upon the completion of the last protocol-specified contact (eg, visits or telephone contacts).

Study treatment may be discontinued prior to completion for the reasons described in **Section 7.3.3**, Subject Discontinuation Criteria. For those subjects who do not complete the study, subject participation will be considered terminated upon the

completion of the last visit or contact (eg, phone contact with the investigator or qualified designee).

The overall study ends when the last remaining subject has completed or has been discontinued from the study.

Once a subject has ended participation in the study, study drug will no longer be available to the subject and he/she will revert to standard care according to his/her personal physician.

7.3 Study Population

The 3 age groups selected, 3 months to <2 years, 2 years to <7 years, and 7 years to <18 years represent distinct groups based on epidemiology characteristics as well as potential differences in age-related maturation that may affect the PK of drugs. Pediatric subjects with neutropenia or those expected to be neutropenic (ANC $\leq 500/mm^3$), following standard intensive induction or consolidation chemotherapy will be selected for the study. Approximately 96 PK-evaluable subjects will be enrolled among the study sites.

7.3.1 Subject Inclusion Criteria

The subject must meet **ALL** the criteria listed below for entry:

1. Children of either sex and of any race, 3 months to <18 years of age.
2. Subjects' parent or legally authorized representative must be willing to give written informed consent. Assent will be obtained from minors according to institutional practices.
3. Subjects must have documented or anticipated neutropenia (ANC $\leq 500/mm^3$ [0.5×10^9/L]) expected to last for at least 7 days and only in the following clinical situations:
   a. Acute leukemia (including new and relapse),
   b. Myelodysplasia,
   c. Severe aplastic anemia,
   d. Autologous HSCT recipients,
   e. High risk neuroblastoma,
   f. Advanced stage non-Hodgkin's lymphoma,
   g. Recipients of allogeneic HSCT during the pre-engraftment period (neutropenia period).

4. Male and female subjects of child-bearing potential must agree to use a medically accepted method of contraception throughout the study and for at least 30 days after stopping the medication, unless they are surgically or medically sterile or agree to abstain from sexual intercourse. Acceptable methods of contraception include **2** of the following:
   a. Condoms (male or female) with spermicide,
   b. Diaphragm or cervical cap (if acceptable according to local standard of care) with spermicide (female),
   c. Hormonal contraceptives or intrauterine device with spermicide (female).

7.3.2 Subject Exclusion Criteria

The subject will be excluded from entry if **ANY** of the criteria listed below are met:

1. Subjects with proven IFI, as defined by the Mycoses Study Group (MSG)/ European Organization for Research and Treatment of Cancer (EORTC) criteria (**Appendix 3**), prior to study entry.
2. Subjects with Grade 3 or Grade 4 nausea and/or vomiting at Screening.
3. Subjects who have received POS within the past 10 days prior to Screening.
4. Subjects receiving prohibited drugs (**Table 3**).
5. Subjects whose laboratory tests are outside normal limits, as follows:
   a. AST or ALT >5 times the upper limit of normal (ULN)
   b. Serum total bilirubin >2.5 x ULN
   c. Calculated creatinine clearance <30 mL/min. Creatinine clearance will be calculated using the following equation:

      Creatinine clearance = k*height (cm)/serum creatinine (mg/dL)

      Where k = 0.45 for a full term baby less than 1 year old; 0.55 for children up to 12 years old; 0.55 for females between the ages of 13 and 21 years; 0.7 for males between the ages of 13 and 21 years.
6. Subjects with QTc prolongation:
   a. Symptomatic QTc prolongation >450 msec (males) or >470 msec (females);
   b. Any QTc prolongation of >500 msec.
7. Subjects who are unable to receive study drug enterally.
8. Female subjects who are pregnant, intend to become pregnant during the course of the study, or are breast-feeding.
9. Subjects with any clinically significant condition or situation, other than the condition being studied that, in the opinion of the investigator, would interfere with the study evaluations or optimal participation in the study, including receiving less than 7 days of POS.

10. Subjects with a history of anaphylaxis attributed to the azole class of antifungal agents.
11. Subjects who have already participated in this study or are participating in any Phase 1 clinical study or any study for a medication that has not yet received regulatory approval. **Note**: If the medication has received a regulatory approval for use in adults, then the medication would be considered to have received a regulatory approval for the purpose of this criterion. Any medication received by eligible subjects must also be aligned with the protocol guidance for prohibited medications (Table 3).
12. Subjects who are part of the study staff personnel or family members of the study staff personnel.

7.3.3 Subject Discontinuation Criteria

Study treatment may be discontinued during the study for any of the following reasons:

- Serious or life-threatening adverse event (AE);
- The subject experiences Grade 3 or 4 toxicity related to the study drug meeting the criteria in **Section 7.7.2.2.2**;
- Failure to comply with the dosing, evaluations, or other requirements of the study;
- Request of the subject or subject's parent or legally authorized representative (subjects have the right to discontinue treatment at any time for any reason);
- Pregnancy;
- Initiation of systemic antifungal agents for empiric or pre-emptive therapy, including agents containing amphotericin B (AMB), echinocandins, systemic azole and triazole antifungal agents or other investigational antifungal drugs;
- Other systemic antifungal agents are indicated for proven IFI;
- Subjects who, following Baseline require any of the prohibited medications listed in **Table 3** of Section 7.4.2.1.1, Medications Prohibited Prior to Study Drug Administration and During the Study Treatment Phase;
- Any situation or condition which may threaten the subject's health or well-being by continuing in the study;
- Death;
- The subject has either recovered from neutropenia (ANC >500/mm^3 [0.5 x 10^9/L]) or received a maximum of 28 days of therapy with POS;
- Administrative (eg, study termination).

A subject who develops a superficial fungal infection (eg, cutaneous fungal infection, thrush or candidal vaginitis) may be treated with topical antifungal agents (nystatin and/or azole formulations) and continued on study drug if in the judgment of the investigator there is no evidence of systemic involvement or more extensive mucosal involvement that would require more specific systemic antifungal therapy. Superficial candidiasis (including signs and symptoms and supporting laboratory findings) must be noted on the eCRF.

It is the right and the duty of the investigator or subinvestigator to interrupt treatment of any subject if he/she feels that study discontinuation is necessary to protect the subject, or that there are unmanageable factors, that may interfere significantly with the study procedures and/or the interpretation of results.

If a subject discontinues prior to completion of the study, the reason for the discontinuation will be obtained. The date of the last dose of study medication and the date of the last assessment and/or contact will be obtained. This information will be documented in the appropriate section of the eCRF. A follow-up contact (telephone or visit) will be arranged as appropriate.

At the time of discontinuation, every effort should be made to ensure all procedures and evaluations scheduled for the final study visit are performed (**Section 2.2**, Study Flow Chart and **Section 7.6**, Study Procedures). For all discontinued subjects, AEs should be recorded and medication compliance should be assessed. Any returned drug should be inventoried.

7.3.4 Replacement of Subjects

If a subject in a given dosage level and age group receives fewer than 14 consecutive doses for BID dosing or 21 doses for TID dosing, or fails to complete Day 8 trough PK sample collection for reasons other than drug intolerance, a replacement subject may be entered at that dosage level and age group.

After consultation between the Sponsor and the principal investigator, enrollment may be extended to replace subject(s) discontinued during the study.

7.4 Treatments

7.4.1 Study Treatments

Upon acceptance into the study, once all the inclusion criteria are met and none of the exclusion criteria are met on Day 1, subjects (2 years to <18 years of age) will be

enrolled. Twelve subjects per dose group will be enrolled in the following age groups: 2 years to <7 years (Age Group 1) and 7 years to <18 years (Age Group 2).

The first dose group of 24 subjects in Age Groups 1 and 2 (2 years to <18 years) will receive POS at a total dose of 12 mg/kg/day, divided twice daily (BID), up to a maximum of 800 mg per day. If no more than 2 of the first 6 subjects in a given age group experience Grade 3 and Grade 4 toxicities in the same organ class which pursuant to protocol item **Section 7.7.2.2** result in definitive discontinuation of study drug treatment and are believed to be potentially cause-related to study drug, initiation of the second dose group may begin. The second dose group of 24 subjects in Age Groups 1 and 2 will receive POS at a dose of 18 mg/kg/day, divided twice daily (BID), up to a maximum of 1200 mg per day. The third dose group for Age Groups 1 and 2 will receive 18 mg/kg/day orally divided into 3 doses (TID), up to a maximum of 1200 mg/day.

The 24 subjects in the youngest age group (Age Group 3 [3 months to <2 years]; n=12/dosage group) will receive POS in the same manner using the same criteria as the two older age groups, starting at the same time as the third dose group in the 2 older age groups (Age Groups 1 and 2). Based on the review of the safety and PK data in the older age groups, the youngest age group (Age Group 3) will start with a total daily dose divided TID, instead of BID. The starting dose for the youngest age group will be 12 mg/kg/day divided into 3 doses, up to a maximum of 800 mg per day. If no more than 2 of the first 6 subjects in a given age group experience Grade 3 and Grade 4 toxicities in the same organ class which pursuant to protocol item **Section 7.7.2.2** result in definitive discontinuation of study drug treatment and are believed to be potentially cause-related to study drug, initiation of the second dose group may begin. The second dose group of 12 subjects in Age Group 3 will receive POS at a dose of 18 mg/kg/day divided into 3 doses, up to a maximum of 1200 mg per day. There will only be 2 dose groups for Age Group 3. If the target POS exposure level is not achieved with POS at a dose of 18 mg/kg/day divided TID in this youngest age group, there will not be further dose escalation for this age group.

In all treatment groups, subjects will receive study drug for at least 7 days, and until recovery from neutropenia (ANC>500/mm^3) or a maximum duration of 28 days, whichever occurs first. All POS doses will be based on actual body weight at baseline which should be continued throughout the treatment period. If four of twelve subjects experience Grade 3 and Grade 4 toxicities in the same organ class which pursuant to protocol item **Section 7.7.2.2** result in definitive discontinuation of study drug treatment and are believed to be potentially cause-related to study drug, then dose limiting toxicity (DLT) will be reached.

7.4.1.1 Treatments Administered

POS oral suspension dosing will be administered using the Sponsor-supplied 1-mL, 3-mL, or 10-mL oral dosing syringe. For infants, doses may be administered by gavage using an appropriately sized oral dosing syringe provided.

Six different sizes of oral dosing syringes (0.5 mL, 1.0 mL, 3.0 mL, 5.0 mL, 10.0 mL, and 20.0 mL) were evaluated in a dose recovery study by the Sponsor, and there was no significant difference found in the recovery of POS oral suspension from these sizes of oral syringes relative to control samples. Various sizes of syringes allow for accurate dosing of POS using the current pediatric dosing procedure.[(17)]

For a subject that has a feeding tube made of polyvinyl chloride (PVC), silicone, or polyurethane inserted for nutritional support and who does not have any contraindication for enteral feeding, POS oral suspension can be administered via the enteral feeding tube (e.g. gastric or nasogastric tube). Feeding tubes made of PVC, silicone, or polyurethane have been evaluated in a study by the Sponsor and were found to be compatible with POS oral suspension.[(17)] There is no information on compatibility or dose recovery with feeding tubes made of other materials.

POS oral doses will be administered within approximately 10 minutes after completion of a meal or oral liquid nutritional supplement, or in the case of infants, after a bottle or breast-feeding. For subjects with enteral feeding tubes, POS oral suspension administration must be followed by a flush of the tube, preferably with the same infusate given for feeding (nutritional supplement). The flush volume should be at least the same volume as the volume of the enteral tube (e.g., 10 mL) taking in consideration the diameter of the tube, manufacturer's specifications, and the local standard of care.

Each dose should be administered at approximately the same time each day, approximately 12 hours apart for subjects receiving BID dosing and approximately every 8 hours for subjects receiving TID dosing. On days when trough sampling is done, meals and dose will be held until the predose blood sample has been obtained. A dietary food intake assessment will be performed on Days 1 through 28 or last day of study drug treatment. See **Appendix 2**, Posaconazole Oral Administration, for the detailed POS oral treatment preparation and administration procedures.

<u>Dosing Groups for Age Group 1 (2 years to <7 years) and Age Group 2 (7 years to <18 years)</u>

Group 1: POS oral suspension 12 mg/kg/day orally divided into 2 doses (BID), up to a maximum of 800 mg per day.

Group 2: POS oral suspension 18 mg/kg/day orally divided into 2 doses (BID), up to a maximum of 1200 mg per day.

Group 3: POS oral suspension 18 mg/kg/day orally divided into 3 doses (TID), up to a maximum of 1200 mg per day.

Dosing Groups for Age Group 3 (3 months to <2 years)

Group 1: POS oral suspension 12 mg/kg/day orally divided into 3 doses (TID), up to a maximum of 800 mg per day.

Group 2: POS oral suspension 18 mg/kg/day orally divided into 3 doses (TID), up to a maximum of 1200 mg per day.

There is no planned third dosing group for Age Group 3.

7.4.1.2 Timing of Dose for Each Subject

All POS oral doses will be taken with food or oral liquid nutritional supplement containing fat calories (eg, Ensure®, Sustacal®, etc). For infants who are being fed either formula or breastmilk exclusively, POS oral doses should be taken after a feeding. This is consistent with the conditions in which studies of the absorption, metabolism, and elimination of oral ^{14}C POS were performed. POS oral doses will be administered within approximately 10 minutes after completion of a meal or oral liquid nutritional supplement. If for any reason the timing of the medication needs to be adjusted after the first dose, the dose time may be adjusted by ± 6 hours for BID dosing and ± 3 hours for TID dosing.

Subjects should be observed for 30 to 60 minutes after the first dose for tolerance of study drug. Subjects who vomit within 30 minutes of POS oral administration should be given a single replacement dose. Repeated episodes of vomiting should be indicated on the Adverse Event eCRF and the Dosing eCRF.

7.4.1.3 Method of Treatment Assignment, Randomization, and/or Stratification

No randomization will be performed. Subjects will be stratified into the following age groups:

Age Group 1: 2 years to <7 years of age

Age Group 2: 7 years to <18 years of age

Age Group 3: 3 months to <2 years of age

7.4.1.4 Management of Blinding of Study Treatments

This is an open-label study with no blinding.

7.4.1.5 Investigational Product

The investigator shall take responsibility for and shall take all steps to maintain appropriate records and ensure appropriate supply, handling, storage, distribution, and usage of these materials in accordance with the protocol and any applicable laws and regulations.

The following Investigational product will be used in the study and will be supplied in sufficient quantities for subjects and any replacement subjects.

- POS Oral Suspension 40 mg/mL, will be supplied as 105-mL fill in 120-mL bottle.

7.4.1.5.1 Identity of Investigational Product(s)

Please see the Investigator's Brochure for description of investigational drug product.[(8)] Subjects will receive the following antifungal agent:

- POS suspension is supplied in 40 mg/mL strength.

7.4.1.5.2 Source

The Sponsor will provide the investigational product for this study.

7.4.1.5.3 Labeling, Storage and Dispensing

Each POS oral suspension bottle will be labeled in a standard open-label fashion in accordance with Good Manufacturing Practice (GMP) for clinical trials and country specific guidelines. The label on each bottle will include the drug name, strength,

study number, a unique alpha-numeric number (Packaging Request number or Lot number), storage conditions and an investigational use statement. Additional country-specific requirements may be added to the label. The label on each POS oral suspension bottle will include the following instructions: Shake well before use. Take with meals or immediately after eating. Spaces will be provided on each oral bottle label for the recording of the study site, patient number, date dispensed, amount to be administered and bottle number.

Study drug supplies must be stored in a secure, limited-access location under the storage conditions specified on the drug supply label.

Receipt and dispensing of study medication must be recorded by an authorized person at the investigator's site.

The investigator agrees neither to dispense the study drug from, nor store it at any site(s) other than those listed on the Form Food and Drug Administration (FDA) 1572 or Investigator’s Agreement. The investigator agrees that study drug(s) will be dispensed by the investigator or subinvestigator(s) named on the Form FDA 1572 or Investigator’s Agreement, or their qualified designees. The investigator, subinvestigators, or qualified designees also agree that the study drug(s) will be dispensed only to study subjects or parents or legal guardians of subjects from whom written informed consent has been obtained, and have met all entry criteria. Clinical supplies may not be used for any purpose other than that stated in the protocol.

An adequate quantity of study drug will be provided to replace units damaged in shipment/handling, to replace subjects meeting the entry criteria or to enroll additional subjects at intermediate or higher dose levels.

7.4.1.5.4 Packaging

Study drug will be packaged in bulk, 6 bottles per box.

7.4.1.5.5 Drug Accountability

The subjects or their parent(s) or legal guardians will be instructed to return all unused and partially used test articles at all protocol-specified visits for drug inventory and assessment of subject compliance.

An accurate and current accounting of the dispensing of study drug(s) for each subject will be maintained on an ongoing basis by a member of the study site staff in

a test article accountability ledger (TAAL) or equivalent document and the eCRF and will be verified by the Sponsor's study monitor.

All drug supplies, including all containers of study drug, whether empty or containing unused or partially used study drug, must be returned to the Sponsor or its designee, unless investigators are instructed otherwise by the Sponsor or its designee. The Sponsor's study monitor will provide instructions on the return of all drug supplies. Under instructions from the Sponsor the investigator or designee may destroy unused or partially used study drug provided the investigator retains sufficient records of the destruction of the study drug. A final inventory of the total amount of drug received at each study site against the amount used and returned must be recorded in the Test Article Summary Inventory Record (TASIR) or equivalent document.

Inventory records must be readily available for inspection by the study monitor and/or auditor, and open to government inspection at any time.

7.4.2 Other Treatments

7.4.2.1 Prior and Concomitant Medications

All prior medication taken by the subject 14 days prior to treatment intervention and all concomitant therapy taken by the subject during the study are to be recorded on the eCRF. Chemotherapeutic agents used for any chemotherapy regimen within 30 days of the first day of dosing will be recorded on the eCRF. The identity of the therapy, the dates started and stopped (or notation of "continuing" if that is the case), and the reason for use must be recorded. The use of any concomitant medication must relate to an AE or the subject's medical history.

Patients receiving other antifungal agents as prophylactic therapy must discontinue these treatments prior to study drug administration. No other systemic antifungal agents may be administered during the Treatment Phase without Sponsor approval.

Nasal sprays of AMB and aerosolized AMB are prohibited during the Treatment Phase. If subjects are on such treatment before study entry, such drugs must be discontinued prior to study drug administration. Investigational drugs (ie, other drugs not yet approved for marketing by the FDA or local health authorities) are also prohibited during the Treatment Phase and up to 30 days after discontinuation of study drug.

Topical nonabsorbable antifungals may be used for the treatment of oropharyngeal candidiasis, vaginal candidiasis, or cutaneous fungal infection. These include: oral AMB, miconazole (oral or topical), nystatin (oral or topical), oral or topical clotrimazole. All other antifungal therapies must be approved by the Sponsor prior

to use. No other topical or oral antifungal agents may be used as prophylactic treatments (eg, clotrimazole as prophylaxis in patients with mucositis). **Table 3** lists the other prohibited medications and recommended washout periods to be observed prior to initiation of POS oral. The washout period for POS after discontinuation of POS oral is approximately 7 days. Subjects should be monitored for untoward reactions if any of the prohibited medications are administered during the POS washout period (7 days post treatment) and AEs related to potential drug interactions should be reported in the eCRF.

The following recommendations should be followed for other concurrent medications:

Ciclosporin: In heart transplant patients on stable doses of ciclosporin, POS 200 mg once daily increased ciclosporin concentrations requiring dose reductions of up to 29%. Cases of elevated ciclosporin levels resulting in SAEs, including nephrotoxicity were reported in clinical efficacy studies. Monitoring of ciclosporin blood levels should be performed upon initiation, during coadministration, and upon discontinuation of POS treatment, with adjustment of ciclosporin doses as necessary.

Tacrolimus: POS increased C_{max} and AUC of tacrolimus (0.05 mg/kg body weight single dose) by 121 % and 358 %, respectively. Clinically significant interactions resulting in hospitalization and/or POS discontinuation were reported in clinical efficacy studies. When initiating POS treatment in patients already receiving tacrolimus, the dose of tacrolimus dose should be reduced (eg, to about one third of the current dose). Thereafter blood levels of tacrolimus should be monitored carefully during coadministration, and upon discontinuation of POS, and the dose of tacrolimus should be adjusted as necessary.

Sirolimus: The co-administration of POS and sirolimus is not recommended. However, if it is required, when initiating therapy in patients already taking sirolimus, the dose of sirolimus should be reduced (eg, to about 1/10 of the current dose) with frequent monitoring of sirolimus whole blood trough concentrations. Sirolimus concentrations should be performed upon initiation, during co-administration, and at discontinuation of posaconazole treatment, with sirolimus doses reduced accordingly.

Antiretroviral Agents: As HIV protease inhibitors and non-nucleoside reverse transcriptase inhibitors (NNRTIs) are CYP3A4 substrates, it is expected that POS will increase plasma levels of these antiretroviral agents. Patients should be carefully monitored for any occurrence of toxicity during the coadministration of POS and these agents. Note that coadministration of POS with atazanavir, efavirenz, fosamprenavir, or ritonavir is not permitted during the treatment phase of the study (**Table 3**).

Midazolam and Other Benzodiazepines Metabolized by CYP3A4: POS 200 mg orally once daily increased the AUC of midazolam by 83% following intravenous (IV) administration. Due to the inhibition of intestinal CYP3A4 by POS, an even greater effect of POS on the AUC of midazolam is expected following oral administration. Dose adjustments should be considered for all benzodiazepines that are metabolised through CYP3A4 (eg, midazolam, triazolam, alprazolam) during coadministration with POS.

Calcium Channel Blockers Metabolized Through CYP3A4 (eg, diltiazem, verapamil, nifedipine, nisoldipine): Frequent monitoring for AEs and toxicity related to calcium channel blockers is recommended during co-administration with POS. Dose adjustment of calcium channel blockers may be required.

Digoxin: Administration of other azoles has been associated with increases in digoxin levels. Therefore, POS may increase plasma concentration of digoxin and digoxin levels need to be monitored when initiating or discontinuing POS treatment.

Sulfonylureas: Glucose concentrations decreased in some healthy subjects when glipizide was coadministered with POS. Monitoring of glucose concentrations is recommended in diabetic patients.

7.4.2.1.1 Medications Prohibited Prior to Study Drug Administration and During the Study Treatment Phase

The medications prohibited prior to study drug administration and during the treatment phase of the study are listed in **Table 3**.

Table 3 Prohibited Medications Prior to Study Drug Administration and During the Study Treatment Phase

Protocol No. P03579

Prohibited Medications During the Study Treatment Phase and Prior to Study Drug Administration	Washout Period[a]
Systemic antifungal therapy (oral, intravenous or inhaled) for the treatment of the IFI	30 days
Medications that are known to interact with azoles and may lead to life-threatening side effects: astemizole, cisapride, ebastine, halofantrine, pimozide, quinidine, and terfenadine	10 days (astemizole)<br>24 hours (others)
Medications known to lower the serum concentration/efficacy of azole antifungals: barbiturates, carbamazepine, cimetidine, isoniazid, phenytoin, rifabutin, rifampin, and St. John’s Wort (hypericum perforatum)	24 hours
Vinca alkaloids (vincristine, vinblastine, or other licensed or investigational members of this class)	24 hours
Ergot alkaloids (ergotamine, dihydroergotamine or other licensed or investigational members of this class)	2 days
Sirolimus (CYP3A4 substrate)	24 hours
Anthracycline-based chemotherapy	24 hours
Prophylactic use of antifungal drugs	24 hours
Investigational drugs (new chemical or biological entities; Phase 1/2A): investigational use of approved products or chemotherapy regimens may be permitted with the approval of the Sponsor’s project physician prior to use	30 days
Trisenox (arsenic trioxides)	30 days
Amsacrine	24 hours
Disopyramide	4 days
Bepridil	10 days
Pentamidine	3 days
Ibutilide	3 days
Haloperidol	10 days
HMG-CoA reductase inhibitors metabolized via CYP3A4 (eg, simvastatin, lovastatin and atorvastatin)	24 hours
Cyclophosphamide	24 hours
Atazanavir, efavirenz, fosamprenavir, and ritonavir	24 hours

CYP = cytochrome P450; HMG-CoA = 3-hydroxy-3-methylglutaryl coenzyme A; IFI = invasive fungal infection; QTc = QT interval corrected for rate; TdP = torsade de pointes.

[a] These waiting times should be observed prior to study drug administration in subjects receiving a prohibited drug as prior therapy. No concurrent use of prohibited medications is permitted. Deviations from these washout periods must be approved by the Sponsor prior to use of study drug or prohibited agent.

7.4.2.1.2 Medications Allowed During the Study

The medications allowed during the study are presented in **Table 4**. Medications prohibited during this study are summarized in **Table 3**:

Table 4 Allowed Medications

Protocol No. P03579

Medication Names
Acetaminophen (paracetamol)
Prophylactic Antibacterial Antibiotics[a]
Empiric Antibacterial Therapy[a]
Topical nonabsorbable antifungals for treatment of superficial infections[b]
Oral or injectable hormonal contraceptives[c]
Medications necessary to treat adverse events, medical emergencies, or underlying disease

a Institutional guidelines should be followed.

b These agents may be used for the treatment of oropharyngeal candidiasis, vaginal candidiasis, or cutaneous fungal infection.

c For those subjects using oral or injectable hormonal contraception, a barrier method of birth control is necessary (eg, condom in combination with spermicide).

Clinical and/or QTc monitoring is recommended when the study drug is coadministered with one of the following drugs that have reported a potential risk of torsades de pointes: antiarrhythmics (amiodarone, dofetilide, procainamide, sotalol), chlorpromazine, clarithromycin, domperidone, droperidol, levomethadyl, mesoridazine, methadone, erythromycin, sparfloxacin, or thioridazine.

The drugs listed below are permitted, although their efficacy and safety should be clinically monitored and/or serum levels followed with appropriate dosage adjustments as necessary at the initiation of study drug, periodically during treatment, and after discontinuation of study drug:

- Oral hypoglycemic agents
- Digoxin
- Coumadin-type anticoagulants
- Calcium channel blockers
- Theophylline
- Antiretroviral therapy (eg, tenofovir).

POS interferes with the hepatic clearance of triazolam and midazolam, and thus, may enhance the sedative effects of these agents. Therefore, these agents are not allowed unless monitoring is provided for excessive sedation.

Use of omeprazole or other proton-pump inhibitors or cimetidine for stress ulcer prophylaxis or treatment are not permitted during the Treatment Phase of the study.

7.4.2.2 Dietary, Tobacco, Alcohol, and Other Restrictions

7.4.2.2.1 Diet

Meals (breakfast, lunch, dinner and snacks) will be of similar nutritional composition for all subjects/groups, with the possible exception of infants being fed formula or breast-milk exclusively, and will be provided around the same time each day.

When meal and blood draw times coincide, blood will be drawn BEFORE the meal is provided.

Water may be consumed ad libitum.

POS oral will be administered with food or oral nutritional supplement containing fat calories (eg, Ensure, Sustacal, etc). POS oral doses will be administered within approximately 10 minutes after completion of the meal or oral nutritional supplement.

7.4.2.2.2 Tobacco

The enrollment of patients who are smokers is not expected to occur in this trial.

7.4.2.2.3 Alcohol

The enrollment of subjects who consume alcohol is not expected to occur in this trial.

7.4.2.2.4 Exercise

Subjects should refrain from rigorous exercise or physical exertion 48 hours prior to entering into the study and during the study.

7.4.2.2.5 Other Restrictions

None.

7.4.3 Procedures for Monitoring Subject Compliance

At all protocol-specified visits, the investigator or qualified designee is to note in the administration record and in the appropriate section of the eCRF whether treatment had been taken per protocol in the preceding interval. If not, the date(s) and reason for each deviation must be recorded. Space is provided on the eCRF, for explanatory comments. In addition, the study staff will maintain an ongoing record of the dispensing, administration and the return (if appropriate) of all study medication for each subject on the TAAL or equivalent document that will be verified by the Sponsor's study monitor.

7.5 Blood Sampling

For subjects weighing >6.5 kg, PK samples will be drawn on Days 1 and 7, immediately prior to oral administration of POS, and at approximately 3, 5, 8, and 12 hours from the time of oral administration of the morning dose for BID dosing. If TID dosing is required, the 12-hour time point will not be collected, and the 8-hour sample for TID must be taken prior to the next dose. The same criteria apply to subjects in Age Group 3 weighing <6.5 kg with the notable exception that no Day 1 PK samples will be collected for these subjects.

In all subjects, POS C_{min} or trough samples (immediately prior to dosing) will be obtained on Days 3, 5, 8, 14 and 28, or within 24 hours after the last dose of study drug for early discontinuation. In addition, laboratory blood tests will be performed at Screening and Baseline and throughout the treatment period on Days 1, 3, 5, 7, 14, 21, and 28 or last day of study drug administration, and at 9 ±2 days of follow-up to assess the safety and tolerance of POS.

7.6 Study Procedures

An overview of the study is provided in the Study Design Diagram in **Section 2.1**. The Study Flow Chart in **Section 2.2** summarizes the study procedures and the timing of the procedures to be performed at each visit. Individual study procedures

are described below. With the exception of samples taken postdose for PK analysis, blood samples for laboratory tests are to be taken prior to study drug administration at each visit.

For details of the procedures for assessment and reporting of AEs, **Section 7.7.2.2**, Assessment and Reporting of Adverse Events.

In order to minimize variability of evaluations, it is preferred that the same individuals perform the same types of evaluations on the same equipment for all subjects at each study site.

At the time of discontinuation, every effort should be made to ensure all procedures and evaluations scheduled for the End of Treatment visit are performed (**Section 2.2**, Study Flow Chart). For all discontinued subjects, AEs should be recorded and medication compliance should be assessed. Any returned drug should be inventoried.

- **Explain Study and Obtain Written Informed Consent:** Screening.

  The investigator or qualified designee will explain the study and all study requirements to the subject and the subject's parent(s) or legal guardian, answer all questions, and obtain written informed consent from the subject's parent(s) or legal guardian before performing any study-related procedure. A copy of the informed consent will be given to the subject and/or the subject's parent(s) or legal guardian. Assent will be obtained from minors according to institutional practices.

- **Screening Number Assignment:** Screening.

  Subjects are to be assigned a screening number when the informed consent is signed.

- **Review Inclusion/Exclusion Criteria:** Screening, Baseline.

  The inclusion and exclusion criteria will be reviewed by the investigator or qualified designee to ensure that the subject qualifies for the study.

- **Demographic Profile:** Screening.

  The demographic profile will include the subject's date of birth, age, ethnicity, gender, and race.

- **Medical History:** Screening.

  A detailed medical history will be obtained by the investigator or qualified designee. Subject history should include information on family history and personal history, history of diseases including past history of hepatitis, allergies or reactions to medications and food, seizures, and previous surgery, trauma, syncope, arrhythmias. Any clinically relevant changes found at any time during the study will be recorded as adverse events.

- **Physical Examination:** Screening, Baseline.

  The principal investigator or designee will perform a physical exam at the times indicated in **Section 2.2**, Study Flow Chart. Any medical conditions found at the Screening and Baseline exams will be recorded on the eCRF in the Medical History module. The status of the condition will be evaluated as "resolved", "stable" or "unstable". Any unstable medical condition will require a comment. An exacerbation of a pre-existing condition or any new finding that occurs during the study will be captured as an adverse event and recorded in the Adverse Event module of the eCRF.

- **Body Weight (kg) Without Shoes:** Baseline, Day 28 or End of Treatment.

  Body weight at each required visit should be performed on the same scale for the same individual. Measurements should be recorded to the nearest kg.

- **Height Measurements (cm) Without Shoes:** Screening.

  Height measurements will be recorded to the nearest cm.

- **Vital Signs:** Screening, Baseline, Days 1, 3, 5, 7, 14, 21, 28 or End of Treatment, Last Study Visit.

  The pretreatment vital signs will be considered the Baseline values.

  Vital signs will be obtained by the principal investigator or designee. Subjects will be in a supine position for at least 3 minutes. Systolic and diastolic blood pressure (mm Hg), pulse rate (bpm) and body temperature (°C or °F) will be obtained according to local standard procedure and will be recorded.

  If the scheduled time for vital sign measurements coincides with a blood collection, the vital signs should be performed prior to the blood collection, or at least 5 minutes afterwards. This same timing for obtaining the vital signs (before or after the blood collection) should be used for all vital sign measurements.

  Body temperature obtained within one hour of blood product transfusions should not be included on the eCRF. On the required days, the maximum daily temperature (outside of blood product transfusions) will be collected and recorded on the eCRF.

- **Clinical Assessment of Electrocardiograms:** Baseline, Day 3.

  Two baseline ECGs must be performed at least 5 minutes apart at Baseline Visit for purposes of protocol eligibility.

  Prior to study drug administration, all ECGs should be reviewed for clinically significant abnormalities and reported as normal or with findings that are not clinically significant.

  ECGs should also be performed as clinically indicated for the evaluation of AEs.

  ECGs performed after the Baseline Visit should be done at the same time of day (AM or PM) that baseline ECGs were performed and prior to a meal.

  A standard 12-lead ECG, reporting ventricular rate, PR, QRS, QT, and QTc intervals, will be performed using equipment provided by the Sponsor for this purpose. Any clinically significant abnormality must be followed until stabilization or return to Baseline.

Any change in the QTc interval of greater than 30 msec post Baseline (when compared to Baseline) will require a repeat ECG, and a determination of serum potassium (K), calcium (Ca), magnesium (Mg), and electrolyte replacement, if necessary. Additional evaluation may be required, and the principal investigator should determine if discontinuation of study drug or other evaluation is required in consultation with the project physician. If the QTc interval change from Baseline is greater than 60 msec, study drug should be interrupted while evaluation and treatment of other etiologies is ongoing (**Section 7.3.3**, Subject Discontinuation Criteria).

During the Treatment Phase, if a QTc interval is found to be abnormal (symptomatic QTc intervals greater than 450 msec [males] or 470 msec [females]) after repeated ECG for confirmation a cardiology consultation is to be requested to determine possible etiologies in addition to or other than study drug should also be performed at the same time (eg, review of other concomitant drugs, and determination of serum Mg, Ca, and K levels). The investigator will determine if discontinuation of study drug is warranted after clinical examination of the subject and pertinent laboratory evaluation, and discussion of the case with the Sponsor's project physician.

ECGs performed will be transferred to a blinded third-party for an evaluation of the QT, QTc (Fridericia and Bazett), PR, and QRS intervals and ventricular rate, as well as an overall clinical interpretation. The final results of the third-party analysis will be considered the definitive ECG data and will be the only ECG data used in the analysis.

Note: When the collection of vital signs, ECGs, and PK samples coincide, the blood samples for PK determination (so that the PK samples are collected on time) should be collected first, then the vital signs, and then the ECG. It is preferred that the ECG be performed at the same time each day (eg, morning) to reduce diurnal variation.

For screening and unscheduled ECGs, a ventricular rate of 60 through the upper limit by age will be considered normal sinus rhythm, a rate <60 bpm will be considered sinus bradycardia, and a rate 10% above the age specific limit will be considered sinus tachycardia.

- **Chest Radiograph:** Baseline.

  A chest radiograph will be performed as clinically indicated to evaluate for fungal infection.

- **Pregnancy Tests:** Screening.

  A serum human chorionic gonadotropin (hCG) test must be performed on females of childbearing potential at Screening. Results must be available prior to dosing. Subjects with a positive pregnancy test at Screening or prior to the first dose will not be allowed to enter into the study (screen failure). Positive results will be recorded on the Adverse Event section eCRF. The pregnancy will be reported and monitored according to **Section 7.7.2.2.7**, Reporting of Pregnancies.

- **Clinical Laboratory Safety Tests (Hematology and Serum Chemistry):** Screening, Days 1, 3, 5, 7, 14, 21, 28 or End of Treatment, Last Study Visit.

  The following will be performed according to standard laboratory procedures. If possible, samples should be collected in the fasted state. (After an overnight fast for morning sample.)

Hematology	Chemistry
RBC	Sodium
Hematocrit	Potassium
Hemoglobin	Chloride
Platelets	Glucose
WBC	Blood urea nitrogen (BUN) or UREA
Neutrophils	Creatinine
Lymphocytes	Calcium
Monocytes	Total protein
Basophils	Albumin
Eosinophils	AST (SGOT)
	ALT (SGPT)
	Total bilirubin
	Alkaline phosphatase
	Uric acid
	Magnesium

  Safety laboratory tests will be performed. If during the trial any laboratory result is outside the reference range and is considered to be clinically significant by the investigator, the test should be repeated at appropriate time intervals until it returns to baseline or becomes a clinically insignificant finding. Any adverse clinically significant change will be recorded on the eCRF as an Adverse Event.

- **Absolute Neutrophil Count:** Baseline, Days 1, 3, 5, 7, 14, 21, 28 or End of Treatment, Last Study Visit.

  ANC will be assessed for all subjects at specified visits as indicated in **Section 2.2**.

- **Blood Samples for Determination of Plasma Concentrations of SCH 56592:** Days 1 and 7 for subjects weighing >6.5 kg; Day 7 only for subjects in Age Group 3 (3 months to <2 years) weighing <6.5 kg.

  One milliliter (1 mL) of blood will be collected at the approximate specified time points (0 [predose], and at approximately 3, 5, 8, and 12 hours after administration of the morning dose for BID dosing) indicated in **Section 2.2**, Study Flow Chart, into the appropriate tubes (**Appendix 1**, Specimen Handling and Shipping Instructions, for sample acquisition, shipping and labeling instructions). If TID dosing is required, the 12-hour time point will not be collected, and the 8-hour sample for TID must be taken prior to the next dose.

  Sample collection times will be recorded.

- **Blood Samples for Pharmacokinetic Trough Concentrations:** Days 3, 5, 8, 14 and 28, or within 24 hours after the last dose of study drug for early discontinuation.

  POS C_{min} or trough samples (immediately prior to the morning dose) will be obtained on Days 3, 5, 8, 14 and 28, or within 24 hours after the last dose of study drug for early discontinuation.

  One milliliter (1 mL) of blood will be collected at the specified time points indicated in **Section 2.2**, Study Flow Chart, into the appropriate tubes (**Appendix 1**, Specimen Handling and Shipping Instructions, for sample acquisition, shipping and labeling instructions).

- **Study Drug Administration:** All visits during the Treatment Phase of the study.

  Refer to **Section 7.4**, Treatments, for study treatments to be administered. The time of dose administration should be recorded in eCRF for each on site dose administration.

- **Record Adverse Events:** Screening, Baseline, all visits during the Treatment Phase of the study, Last Study Visit.

  SAEs will be captured beginning with signature of the informed consent until 30 days after administration of the last dose of study drug.

  See **Section 7.7.2.2**, Assessment and Reporting of Adverse Events.

- **Record Concomitant Medications:** Screening, Baseline, all visits during the Treatment Phase of the study, Last Study Visit.

  Review of all appropriate medication washout times will be discussed with the subject at Screening. All medications used during the 14 days prior to first drug administration or treatment intervention will be recorded on the eCRF. Chemotherapy used within 30 days prior to first drug administration or treatment intervention will be recorded on the eCRF.

  See **Section 7.4.2**, Non-study Treatments. See **Section 7.3.3**, Discontinuation Criteria.

- **Dietary Food Intake Assessment:** All visits during the Treatment Phase of the study.

  Each subject and/or the subject's parent(s) or legal guardian will keep a diary of food consumed with study drug administration throughout the treatment period. For bottle fed infants, the number of feedings, timing and amount consumed should be recorded. For infants who are breastfed exclusively, the timing of each feeding should be recorded. A dietitian/knowledgeable healthcare provider will analyze the information in the food intake diary to summarize the quantity (full intake, partial diet, or fasting) of food consumed and to estimate the percent of fat consumed by the subject with administration of each dose. This information will be entered by study center personnel into the eCRF module.

- **Survival Assessment:** Any day from Days 60 to 70.

  Survival assessment, if the subject is alive or dead, will be performed any day from Days 60 to 70. If the subject dies, the date of death will be recorded.

7.7 Study Analysis Plan

7.7.1 Pharmacokinetics

Plasma SCH 56592 concentration data will be used to estimate the following primary PK variables for the determination of bioavailability comparisons:

C_{min} -	POS trough level immediately before a subject receives the dose on the day specified in the protocol.
C_{max} -	Maximum plasma concentration.
T_{max} -	Time of maximum plasma concentration.
AUC(tf) -	Area under the plasma concentration versus time curve from time 0 to the time of the final quantifiable sample.
AUC(τ) -	Area under the plasma concentration versus time curve during a dosing interval (τ) at the steady state.
CL/F -	Total apparent body clearance at the steady state.

The current blood samples schedule is an optimized schedule based on the adult steady-state PK data. If all blood samples can be collected at steady-state as

scheduled (Day 7), the steady-state AUC will be calculated using the non-compartmental trapezoidal method.(16)

If more than one blood sample will be available for a patient, the concentration values will be averaged (C_{avg}). If data allow a calculation of AUC using a non-compartmental trapezoidal method,(16) the C_{avg} will also be calculated by dividing the AUC with dosing interval.

7.7.2 Safety

7.7.2.1 Specification of Safety Variables

Safety variables to be assessed include: AEs, hematology and blood chemistry, vital signs, physical examinations, and ECGs. The key AEs to be evaluated for this study are treatment-emergent, treatment-related cardiac events, hepatic events, pulmonary embolism, and thrombocytopenia. Specifically, clinical laboratory tests, changes in vital signs, and changes from baseline ECG readings (QT/QTc intervals) will also be measured to determine the safety and tolerability of POS in each dose group.

7.7.2.2 Assessment and Reporting of Adverse Events

An adverse event (AE) is any untoward medical occurrence in a subject administered a pharmaceutical product, biologic (at any dose), or medical device, which does not necessarily have a causal relationship with the treatment. AEs may include the onset of new illness and the exacerbation of pre-existing conditions.

Additionally, any event that is associated, or observed in conjunction, with a product overdose (whether accidental or intentional) or a product abuse and/or withdrawal is also considered an AE.

All AEs must be recorded in the subject's medical records and on the eCRF. The onset and end dates, severity and relationship to study drug will be recorded for each AE. The severity of the AE will be assessed according to specific guidelines (**Section 7.7.2.2.1**, Assessment of Adverse Event Severity and Relationship to Treatment). Any action or outcome (eg, hospitalization, discontinuation of therapy, etc) will also be recorded for each AE.

Subjects and/or the subject's legal guardian will be questioned and/or examined by the investigator or a qualified designee for evidence of AEs. The questioning of subjects with regard to the possible occurrence of AEs will be generalized such as,

"How have you been feeling since your last visit?" The presence or absence of specific AEs should not be elicited from subjects.

7.7.2.2.1 Assessment of Adverse Event Severity and Relationship to Treatment

Where the determination of AE severity rests on medical judgment, the determination of severity must be made with the appropriate involvement of a medically-qualified investigator.

The National Cancer Institute's (NCI) Common Terminology Criteria for Adverse Events (CTCAE) grading system will be used for grading severity of AEs (see http://ctep.cancer.gov/reporting/ctc.html). The site will obtain the current version of the grading system, confirm the version with the study monitor and make a copy available to the Institutional Review Board (IRB)/Ethics Committee (EC). For AEs not covered by this grading system, the following definitions will be used:

Mild:	awareness of sign, symptom, or event, but easily tolerated;
Moderate:	discomfort enough to cause interference with usual activity and may warrant intervention;
Severe:	incapacitating with inability to do usual activities or significantly affects clinical status, and warrants intervention;
Life-Threatening:	immediate risk of death.

A medically-qualified investigator must assess the relationship of any AE to the use of study drug, based on available information, using the following guidelines:

Unlikely related:	no temporal association, or the cause of the event has been identified, or the drug, biological, or device cannot be implicated;
Possibly related:	temporal association, but other etiologies are likely to be the cause; however, involvement of the drug, biological, or device cannot be excluded;

Probably related: temporal association, other etiologies are possible, but unlikely.

The expectedness of an adverse reaction shall be determined in each country according to the specified reference document containing safety information. The reference document containing safety information for the investigational medicinal product (IMP), Posaconazole, Aqueous Oral Suspension, in this current study is to be the most recent Investigator's Brochure for Posaconazole, SCH 56592, Aqueous Injectable Suspension, Aqueous Oral Suspension.[(8)]

7.7.2.2.2 Monitoring Adverse Events

Subjects having AEs will be monitored with relevant clinical assessments and laboratory tests, as determined by the investigator. The investigator or qualified designee is expected to report ongoing AEs at completion of the clinical study to the primary care physician who will determine the need for and provide standard medical care.

Any actions taken and follow-up results must be recorded either on the appropriate page of the eCRF or in a follow-up letter to the Sponsor, as well as in the subject's source documentation. Follow-up laboratory results should be filed with the subject's source documentation.

For all AEs that require the subject to be discontinued from the study, relevant clinical assessments and laboratory tests will be repeated as clinically appropriate, until final resolution or stabilization of the event(s).

Adverse event reporting will be conducted as follows:

- **Infections**
  All infections or clinically diagnosed infections (ie, no pathogen identified) are considered AEs. (The infection diagnosis should be recorded on the AE screen, not the individual signs and symptoms of infection [eg, pneumonia or bacteremia, not dyspnea or positive blood culture]).
- **Laboratory Abnormalities**
  Laboratory abnormalities which have clinical manifestations or which require an intervention should be recorded on the AE screen; use a clinical term if applicable.

- **Minor or Routine Surgical Procedures**

  All minor surgical procedures (unless pre-planned as part of routine care, eg, Hickman catheter placement) and the reason for the procedure are AEs. (For example, removal of a Hickman catheter for thrombosis after study drug has been given would be reported as an AE, ie, incision and drainage of abscess requiring admission to the hospital would be indicated as an SAE with outcomes of hospitalization and additional therapy.)

- **Body Weight**

  Greater than 10% change in body weight (gain or loss) from Baseline will be considered an AE unless an increase in body weight is predicted based on standard growth charts applicable to the study center population.

Grade 4 Adverse Events

The following guidelines will be used regarding continuation of treatment:

- If considered probably related to study drug, subject will be discontinued from treatment.
- If considered possibly related to study drug: the subject will be discontinued from treatment, except in the case of AEs for which a relationship to the primary disease and/or other concomitant drugs is equally likely. In these cases, a decision to discontinue study drug or interrupt study drug will be made by the investigator, in consultation with the project physician or designee. If the AE again worsens to Grade 4 after study drug has been reintroduced, the subject must be discontinued from treatment.
- If considered unlikely to be related to study drug: study drug may be continued at the discretion of the investigator, in consultation with the project physician or designee.

Grade 3 Adverse Events

Outcomes for **Grade 3** AEs include the following:

- If a subject experiences a **Grade 3** AE considered at least possibly or probably related to study drug, a decision to continue, interrupt or discontinue study drug will be made by the investigator (in consultation with project physician or designee), taking into account the event severity, clinical significance, treatment options, and likelihood of relationship to study drug versus underlying disease and/or other concomitant drugs.

- If a subject experiences a **Grade 3** AE considered unlikely to be related to study drug, study drug may be continued.

Less Severe Adverse Events

Interruption or discontinuation of study drug may be appropriate in some cases for less severe AEs, which are medically significant. In such cases, the investigator should consult with the project physician or designee to decide the most appropriate course of action with regard to dosing.

7.7.2.2.3 Known Adverse Events Relating to the Underlying Clinical Condition

The following AEs are expected with myelodysplastic syndromes and their treatment: pancytopenia (leukopenia, anemia, and/or thrombocytopenia); manifestations of anemia such as pallor, fatigue, and dyspnea on exertion; manifestations of thrombocytopenia such as petechiae, easy bruising, epistaxis, gingival bleeding, or prolonged bleeding from trauma sites; hepatomegaly, splenomegaly, bone pain, elevated lactate dehydrogenase (LDH) serum levels, and increased uric acid levels. Other manifestations may be pathognomonic of specific primary diagnoses. Alopecia and mucositis are expected AEs with most chemotherapeutic regimens. Any AE which is worse than expected with the primary disease or its treatment (ie, greater severity or more prolonged in duration) should be reported as an AE in the eCRF. All expected Grade 4 AEs, other than alopecia, will also be reported in the eCRF and may require reporting as an SAE.

Infectious complications are common and expected during the course of the primary disease, and are considered AEs.

7.7.2.2.4 Known Potential Toxicities of Study Drug

Clinical studies show that POS demonstrates a favorable safety profile in its oral formulation and appears to be similar to FLU in the cumulative experience from randomized controlled clinical trials in treatment and prophylaxis. The anticipated side effects from POS (treatment-related, treatment-emergent adverse events) are gastrointestinal events (nausea, vomiting, abdominal pain), hepatic events (including asymptomatic elevations in transaminases and/or bilirubin), headache, and asthenia. These side effects are anticipated to be mild and self limited with conservative medical therapy.

Refer to the Investigator's Brochure for additional information on AEs observed to date.(8) The AEs mentioned under this section still need to be recorded in the subject's medical records and on the eCRF regardless of causality.

7.7.2.2.5 Definition of Serious Adverse Events

A serious adverse event (SAE) is any adverse drug or biologic or device experience occurring at any dose that results in any of the following outcomes:

- death;
- life-threatening AE (ie, one that places the subject, in the view of the initial reporter, at immediate risk of death from the AE as it occurs);
- persistent or significant disability/incapacity;
- requires in-patient hospitalization (ie, admission), or prolongs hospitalization;
- congenital anomaly or birth defect.

Additionally, **important medical events** that may not result in death, be life-threatening, or require hospitalization may be considered an SAE when, based upon appropriate medical judgment, they may jeopardize the subject and may require medical or surgical intervention to prevent one of the outcomes listed in this definition. Examples of such medical events include allergic bronchospasm requiring intensive treatment in an emergency room or at home, blood dyscrasias or convulsions that do not result in in-patient hospitalization, or the development of drug dependency or drug abuse.

All SAEs, whether or not deemed drug-related or expected, **must be reported** by the investigator or qualified designee to the Sponsor's designated safety/compliance officer **within 1 working day of first becoming aware of the event**. If the report is given to the Sponsor via telephone rather than in writing on the form designated for SAE reporting, a full description of the event and any sequelae, including the investigator-determined causality to study drug must be provided, so that the appropriate written report can be completed by the designated Sponsor contact. SAEs that occur at any time after the inclusion of the subject in the study up to 30 days after the subject completes study treatment or discontinues the study must be reported. In the specific circumstance of screen failures, SAEs must be collected from the time of consent signing until the subject is considered a screen failure.

Reports of all **SAEs** must be communicated as soon as possible to the appropriate Institutional Review Board (IRB) or Independent Ethics Committee (IEC) and/or reported in accordance with local laws and regulations.

7.7.2.2.6 Reporting of Subject Death

The **death of any subject** after enrollment or within 30 days after the subject completes study treatment or discontinues the study regardless of the cause, **must be reported** by the investigator or qualified designee to the Sponsor’s designated safety/compliance officer **within 1 working day of first becoming aware of the death**. If the report is given via telephone rather than in writing on the form designated for SAE reporting, a full description of the circumstances, including the investigator-determined causality to study drug must be provided, so that the appropriate written report can be completed by the designated Sponsor contact.

If an autopsy is performed, the report must be provided to the Sponsor.

Reports of all deaths, must be communicated as soon as possible to the appropriate IRB or IEC and/or reported in accordance with local law and regulations.

7.7.2.2.7 Reporting of Pregnancies

Although **not** considered an SAE (unless an event occurs with a serious outcome), pregnancy information on clinical study subjects is collected by the Sponsor's Drug Safety Surveillance (DSS) department. If a female subject or the female partner of a male subject becomes pregnant during the course of the study, the investigator or qualified designee must contact the Sponsor’s designated safety/compliance officer within 5 working days of the investigator or qualified designee first becoming aware of the pregnancy. If an SAE occurs in conjunction with the pregnancy, then the reporting time frame for an SAE (1 working day) must be met. The Sponsor’s representative will provide instructions on how to collect pregnancy information. Follow-up information on the outcome of the pregnancy should also be forwarded to the Sponsor.

7.7.2.2.8 Preplanned Hospitalizations or Procedures

During the study, if a subject has a hospitalization or procedure (eg, chemotherapy, placement of central catheter, elective surgery) that was scheduled prior to the subject entering the study (ie, before the subject signed the informed consent) for an event/condition that occurred before the study, the hospitalization is considered a therapeutic intervention and not the result of an SAE. However, if the event/condition worsens during the study, it must be reported as an AE (or SAE, if the event/condition results in a serious outcome such as hospitalization).

7.7.2.2.9 Reports of Overdose

An overdose of POS is any dose more than 1600 mg orally per day.

Information on overdoses in clinical subjects is collected by the Sponsor's DSS department. Should a subject experience an overdose during the course of the study (whether symptomatic or not), the investigator or qualified designee must contact the Sponsor's designated safety/compliance officer, within 5 working days of the investigator or qualified designee first becoming aware of the overdose. Follow-up information on the outcome of the overdose should be forwarded to the Sponsor.

Any event associated with, or observed in conjunction with, a product overdose (whether accidental or intentional) or a product abuse and/or withdrawal is considered an AE and should be reported as such (**Section 7.7.2.2**). If an SAE occurs in conjunction with the overdose, then the reporting time frame for an SAE (1 working day) must be met. The Sponsor's representative will provide instructions on how to collect this information.

7.7.2.2.10 Protocol-Specific Exceptions to SAE Reporting to Drug Safety Surveillance

The following will **not** be considered SAEs:

1. An event that results in hospitalization or prolongs an existing hospitalization will not be considered a serious adverse event if the only reason for the hospitalization was:
   a. administration of chemotherapy
   b. transfusion of blood products
   c. administration of study procedures
   d. placement of a permanent intravenous catheter
   e. hospice placement for terminal care
   f. outpatient hospitalization for procedures such as elective day surgery or hospitalization due to convenience purposes (eg, transportation difficulties)
2. Any Grade 3 or 4 leukopenia, absolute neutropenia, or thrombocytopenia (regardless of baseline value) will not be considered a serious adverse event, even in the event of medical intervention to prevent medically significant sequelae.

3. Any Grade 1, 2, or 3 decrease in hemoglobin will not be considered a serious adverse event.

If an abnormal laboratory value included in item numbers 2 or 3 resulted in a clinical event, then the clinical event and not the abnormal laboratory value must be recorded as an AE in the eCRF. This clinical event must be reviewed to determine if it meets the criteria for an SAE.

7.7.2.3 Reporting of Investigational Medicinal Product Quality Complaints

Any defect or possible defect in an investigational medicinal product (defined as a pharmaceutical form of an active substance or placebo being tested or used as a reference in a clinical trial) must be reported by the investigator or qualified designee to the Sponsor's designated safety coordinator **within 1 working day** of first becoming aware of the possible defect. This report to the Sponsor may be made by telephone or by faxing the Research and Development / Commercialization Quality (RCQ) Clinical Supply Complaint GCP Inquiry Form to the designated Sponsor representative. The product and packaging components in question, if available, must be stored in a secure area under specified storage conditions until it is determined whether the product is required to be returned for investigation of the defect. If the product complaint is associated with an SAE, the SAE must be reported separately in accordance with the protocol, and the SAE report should mention the product quality complaint.

7.7.2.4 Data Monitoring Committee

The safety of subjects in this current trial will be monitored on an ongoing basis by the Sponsor and by an external Data Monitoring Committee.

8.0 STATISTICAL AND ANALYTICAL PLANS

Approximately 96 PK-evaluable subjects (12 subjects each in Age Groups 1, 2, and 3 for Dose Groups 1 and 2 plus 12 subjects each in Age Groups 1 and 2 for Dose Group 3) are expected to be enrolled.

8.1 Data Sets

The PK analysis will only include subjects who received at least one dose of drug. All subjects who received at least one dose of the drug will be included in the safety analyses.

8.2 Demographic and Other Baseline Characteristics

Demographic variables (eg, sex, race, age, weight) will be listed and summarized using descriptive statistics for the entire study population and for each treatment.

8.3 Pharmacokinetic Analyses

8.3.1 Pharmacokinetic Parameters

Single dose and multiple dose plasma concentrations and derived PK parameters for POS will be listed and summarized by dose and age group using descriptive statistics.

The PK parameters, C_{avg} (and C_{max} and AUC if data allow), will be log transformed for statistical analyses. Point estimates and 90% confidence intervals will be provided for each age and dose level. If the confidence intervals indicate that ages do not produce a statistical difference on a PK parameter, then age groups will be pooled. To assess preliminary dose proportionality, log transformed, dose normalized C_{avg} (and C_{max} and AUC if data allow) will be analyzed using analysis of variance (ANOVA) extracting the effect due to treatment. Ratio estimates and 90% confidence intervals will be calculated for the differences between doses and ages (if age groups are not pooled). The steady state analysis will be conducted using available PK trough values.

Comparison of the exposure from previous experience in adults will be conducted at steady state using graphics. A comparison between adult and pediatric patients with respect to the proportion of patients with steady-state C_{avg} greater than or equal to 500 ng/mL will also be conducted. If appropriate, comparisons between BID and TID dosing with respect to the steady-state C_{avg} and the proportion of patients achieving exposure above 500 ng/mL will be made. Preliminary analysis will include examining the PK parameters for extreme values by reviewing the standardized ranges of deviations from the expected value derived from the model to see if any value exceeds 3. The impact of any outlier on the results of the analyses will be evaluated.

8.4 Safety

8.4.1 Adverse Events

All AEs noted during the study will be listed. The number of subjects reporting each AE (by dose and age group) and severity will also be presented. Treatment emergent and treatment-related AEs will be tabulated by body system/organ class.

8.4.2 Clinical Laboratory Tests

The results of hematology and blood chemistry will be listed for each subject. Laboratory values outside the normal ranges will be flagged. If applicable, summary statistics (mean, range, change from baseline) by age/dose group will be provided for each time point.

8.4.3 Vital Signs

Systolic and diastolic blood pressures, heart rate, and body temperature will be listed for each subject. If applicable, summary statistics (mean changes, % change from baseline, and absolute change from baseline) by age/dose group.

8.4.4 Physical Examination

The results of the physical examinations at Screening and/or Baseline will be listed in the medical history listing. Post-baseline findings of the physical examinations that meet the criteria of an AE (**Section 7.7.2.2**, Assessment and Reporting of Adverse Events) will be listed in the relevant AE listings.

8.4.5 Electrocardiogram

The results of the ECG will be listed for each subject. If applicable, summary statistics of ECG results will be provided by age/dose group.

8.4.6 Other Safety

Key toxicities, including hepatotoxicity and nephrotoxicity, will be tabulated by dose and age groups. If 4 or more out of 12 subjects within a dose/age group experience Grade 3 and Grade 4 toxicities in the same organ class which pursuant to protocol item **Section 7.7.2.2** result in definitive discontinuation of study drug treatment and are believed to be potentially cause-related to study drug, then DLT is reached. Assuming the chance of having grade 3/4 toxicity for a subject is 50%, then the probability of having 0, 1, 2, 3 or 4 subjects experiencing grade 3/4 toxicity within a dose/age group is 0%, 0%, 2%, 7% and 19%, respectively.

8.5 Determination of Sample Size/Power/Level of Significance

The planned maximum sample size is 96 PK-evaluable subjects (12 subjects each in Age Groups 1, 2, and 3 for Dose Groups 1 and 2 plus 12 subjects each in Age Groups 1 and 2 for Dose Group 3). With a goal of achieving a C_{avg} in the targeted (500 ng/mL-2500 ng/mL) range in ~90% of subjects, a dose may be considered a success from a statistical point of view if it is observed that at least 10 out of 12 subjects (≥83%) fall within the range for a particular age group. The probability of observing 83% or higher is 89%, 74%, or 56% if the true probability of being within the range is 90%, 85%, or 80%, respectively.

8.6 Interim Analysis

Statistical analysis may be conducted after completion of each group as well as those analyses specified in **Section 2.1.1**.

Importantly, a PK and safety analysis of all available TID data across age groups will be performed after Age Group 2 Dose Group 3 completes enrollment of 12 PK-evaluable subjects.

8.7 Other Analyses

Not applicable.

9.0 ADHERENCE TO ETHICAL, REGULATORY, AND ADMINISTRATIVE CONSIDERATIONS

The study must be conducted in accordance with Good Clinical Practice (GCP) as outlined in the International Conference on Harmonisation of Technical Requirements for Registration of Pharmaceuticals for Human Use (ICH) Guidelines, E6 Good Clinical Practice: Consolidated Guidance and other applicable laws and regulations. In addition, the study must be conducted in accordance with: (i) the USA Code of Federal Regulations (CFR) if the study is conducted under a USA IND, regardless of the country involved; (ii) the European Union (EU) Clinical Trial Directive (CTD) and local regulations if the study is conducted in the EU; and (iii) any specific local regulations if the study is conducted elsewhere.

9.1 Ethical Conduct of the Study

9.1.1 Independent Ethics Committee or Institutional Review Board

Prior to initiation of the trial at any site, the trial, including the protocol, informed consent, and other trial documents must be approved by an appropriate Institutional Review Board (IRB) or Independent Ethics Committee (IEC). The IRB/IEC must be constituted according to applicable regulatory requirements. As appropriate, amendments to the protocol must also be approved by the IRBs/IECs before implementation at the sites. The IRB/IEC approval should be obtained in writing, clearly identifying the trial, the documents reviewed (including informed consent), and the date of the review. The trial as described in the protocol (or amendment), informed consent, and other trial documentation may be implemented only after all the necessary approvals have been obtained and the Sponsor has confirmed that it is acceptable for the investigator to do so.

In the event that the IRB/IEC requires changes in the protocol, the Sponsor shall be advised and must approve the changes prior to implementation. The investigator shall not modify the study described in the protocol once finalized and after approval by the IRB/IEC without the prior written approval of Sponsor.

In countries where the investigator submits the study protocol and statement of informed consent to the IRB/IEC, the investigator or qualified designee will forward the approvals to the Sponsor.

9.1.2 Subject Information and Consent

The details of the protocol must be discussed with each potential subject and/or the subject's parent(s) or legal guardian, and written informed consent must be obtained

for all subjects before any study-related procedure is performed. In obtaining informed consent, the information must be provided in language and terms understandable to the subject. The subject, or the subject's legal representative, must give their written consent to participate in the trial. The signed and dated consent form itself must be retained by the investigator as part of the trial records. A copy of the signed and dated consent form must be given to the subject. The consent form must include all of the required elements of informed consent in accordance with ICH Guidelines E6 and local laws. In addition, the Sponsor specifically requests that the consent form identify it as the Sponsor and state that use of the drug is experimental and the side effects of the drug are not completely known. The consent form must be approved by the appropriate IRB/IEC and Sponsor before study initiation at a study site. Any subsequent changes to the approved informed consent form must be reviewed and approved by the appropriate IRB/IEC and Sponsor before implementation.

9.1.3 Protocol-Related Regulatory and Ethical Considerations/Issues

None.

9.2 Reporting to Sponsor

The investigator will ensure that there are sufficient time, staff, and facilities available for the duration of the trial to conduct and record the trial as described in the protocol and according to all applicable guidances, laws, and regulations.

Any records or documents used as the source of information in the eCRF (called the “subject source data”) are to be retained for review by authorized representatives of the Sponsor or a regulatory agency.

The investigator must maintain records and data during the trial in compliance with all applicable legal and regulatory requirements. The investigator is to provide subject data on completed eCRFs, according to the Sponsor’s instructions and compliant with GCP practices. eCRFs and the electronic database from the trial are the exclusive property of Sponsor.

All data collection forms, be they CRFs, electronic Case Report Forms (eCRF), or other Electronic Data Capture (EDC) screens, should be completed as soon as possible after the evaluation has occurred. All dates appearing on the Sponsor's subject data collection forms for laboratory tests, cultures, and other data collected, must be the dates on which the specimens were obtained, or the procedures performed.

A CRF must be completed for all subjects as per **Section 7.2**, Participation in and Completion of the Study. The Sponsor must not collect subject names, initials, or other personal information that is beyond the scope of the trial from any subject. Subjects are not to be identified by name or initials on the CRF or any trial documents. The only acceptable identification for a subject that may appear on a CRF or trial document is the unique subject identification number. The investigator must maintain contact information for each participant so that all can be quickly contacted by the investigator, if necessary.

A CRF or screening log with a minimum of the following information shall be completed for subjects who fail screening: (1) demographics, (2) subject status, (3) reason for screen failure, and (4) serious adverse events. Data are to be collected from the time the informed consent form is signed until the subject is determined to have failed screening.

All entries into CRFs are the responsibility of the investigator and must be completed by the investigator or a qualified designee. The investigator will acknowledge in writing that he/she has verified the accuracy of the recorded data.

9.3 Publications and Other Rights

9.3.1 Rights to Publish by the Investigator

The investigator has the right to publish or publicly present the results of the study in accordance with this **Section 9.3** of the protocol. In the event that the protocol is a part of a multi-site study, it is understood that it is the intent of the Sponsor and the investigator to initially only publish or present the study results together with the other sites, unless specific written permission is obtained in advance from the Sponsor to publish separate results. The Sponsor shall advise as to the implications of timing of any publication in the event clinical trials are still in progress at sites other than the investigator's site.

The investigator agrees not to publish or publicly present any interim results of the study without the prior written consent of the Sponsor. The investigator further agrees to provide to the Sponsor 45 days prior to submission for publication or presentation, review copies of abstracts or manuscripts for publication (including, without limitation, slides and texts of oral or other public presentations and texts of any transmission through any electronic media, eg, any computer access system such as the Internet, World Wide Web, etc) that report any results of the study. The Sponsor shall have the right to review and comment with respect to publications, abstracts, slides, and manuscripts and the right to review and comment on the data analysis and presentation with regard to the following concerns:

1. proprietary information that is protected by the provisions contained in **Section 9.3.2**;
2. the accuracy of the information contained in the publication; and
3. to ensure that the presentation is fairly balanced and in compliance with FDA regulations.

If the parties disagree concerning the appropriateness of the data analysis and presentation, and/or confidentiality of the Sponsor's confidential information, investigator agrees to meet with the Sponsor's representatives at the clinical study site or as otherwise agreed, prior to submission for publication, for the purpose of making good faith efforts to discuss and resolve any such issues or disagreement.

9.3.2 Use of Proprietary or Confidential Information in a Publication

No publication or manuscript shall contain any trade secret information of the Sponsor or any proprietary or confidential information of the Sponsor and shall be confined to new discoveries and interpretations of scientific fact. If the Sponsor believes there is patentable subject matter contained in any publication or manuscript submitted for review, the Sponsor shall promptly identify such subject matter to investigator. If Sponsor requests and at Sponsor's expense, investigator shall use its best efforts to assist Sponsor to file a patent application covering such subject matter with the USA Patent and Trademark Office or through the Patent Cooperation Treaty prior to any publication.

9.3.3 Use of Trial Information in a Publication

Investigator is granted the right subject to the provisions of this protocol to use the results of all work provided by investigator under this protocol, including but not limited to, the results of tests and any raw data and statistical data generated for investigator's own teaching, research, and publication purposes only. Investigator/Institution agrees, on behalf of itself and its employees, officers, trustees, and agents, not to cause said results to be knowingly used for any commercial purpose whatsoever except as authorized by the Sponsor in writing.

9.3.4 Authorship of Publications

Authors of publications must meet the International Committee of Medical Journal Editors (ICMJE) guidelines for authorship and must satisfy the 3 criteria that follow:

1. Authors must make substantial contributions to the conception and design of the trial, acquisition of data, or analysis of data and interpretation of results;
2. Authors must draft the publication or, during draft review, provide contributions (data analysis, interpretation, or other important intellectual content) leading to significant revision of the manuscript with agreement by the other authors;
3. Authors must provide written approval of the final draft version of the publication prior to submission.

All contributors who do not meet the 3 criteria for authorship should be listed in an acknowledgments section within the publication, if allowed by the journal, per the ICMJE guidelines for acknowledgment.

9.4 Shipping of Hazardous or Dangerous Goods

It is the responsibility of the investigator to ensure that all staff personnel who will be handling, packaging, and/or shipping clinical specimens or any other hazardous or dangerous goods act in conformance with International Air Transport Association (IATA) regulations relating to the handling and shipping of hazardous or dangerous goods.

9.5 Trial Documents and Records Retention

During the trial and after termination of the trial – including after early termination of the trial – the investigator must maintain copies of all documents and records relating to the conduct of the trial. This documentation includes, but is not limited to, protocols, CRFs, advertising for subject participation, adverse event reports, subject source data, correspondence with health authorities and IRBs/IECs, consent forms, investigator's curricula vitae/biosketch, monitor visit logs, laboratory reference ranges, and laboratory certification or quality control procedures and laboratory director curriculum vitae. Subject files and other source data must be kept for the maximum period of time permitted by the hospital, institution or private practice, or as specified below. The study monitor must be consulted if the investigator wishes to assign the files to someone else, remove them to another location, or is unable to retain them for the specified period.

The investigator must retain trial records for the amount of time specified by applicable laws and regulations. At a minimum, trial records must be retained for the

amount of time specified by ICH Guidelines or the EU Good Clinical Practices Directive, whichever is longer:

1. The ICH Guidelines specify that records must be retained for a minimum of 2 years after a marketing application for the indication is approved (or not approved) or 2 years after notifying the appropriate regulatory agency that an investigation is discontinued.
2. The EU GCP Directive specifies that trial records must be retained for 5 years after the completion of the trial.

All trial documents shall be made available if required by relevant health authorities. The investigator should consult with the study monitor prior to discarding trial and/or subject files.

Sponsor will retain all Sponsor-required documentation pertaining to the trial for the lifetime of the product. Archived data may be held on microfiche or electronic record, provided that a back-up exists and that a paper copy can be obtained from it, if required.

10.0 INVESTIGATORS AND STUDY ADMINISTRATIVE STRUCTURE

10.1 Sponsor

The Sponsor of this study is indicated in **Section 1**, Title Page.

10.2 Investigators

10.2.1 Selecting Investigators

Only investigators qualified by training and experience to perform a clinical investigation with POS are selected. The Sponsor will contact and select all investigators or coinvestigators (ie, the legally responsible party[ies] at each study site), who, in turn, will select their staff.

10.2.2 Financial Disclosure Requirement

In connection with the clinical study described in the protocol, the investigator certifies that, if asked, the investigator will read and answer the Clinical Investigator Financial Certification Form truthfully and to the best of investigator's ability. Investigator also certifies that, if asked, the investigator will have any other applicable party(ies) (eg, subinvestigators) read and answer the Clinical Investigator Financial Certification Form as a condition of their participation in the study.

If the financial interests reported on the Clinical Investigator Financial Certification Form change during the course of the study or within 1 year after the last subject has completed the study as specified in the protocol, the investigator and the other applicable party(ies) are obligated to inform the Sponsor of such financial change.

10.2.3 Clinical Study Report Coordinating Investigator

A clinical study report (CSR) will be prepared by the Sponsor or its qualified designee to describe the results of the study. One of the investigators shall be selected by the Sponsor to review the CSR and provide approval of the final CSR in writing. The investigator chosen to review and approve the CSR is to be called the CSR Coordinating Investigator. A second investigator shall be selected as the Alternate CSR Coordinating Investigator. The Alternate CSR Coordinating Investigator is to review and approve the CSR should the first CSR Coordinating Investigator be unable to do so. The Sponsor is to select the CSR Coordinating Investigator and Alternate CSR Coordinating Investigator from the investigators using the following criteria:

- Expertise in the area of PK in pediatrics;
- Active participation during the trial; and/or
- Active participation during the analysis of the results.

10.3 Central Organizations

Central organizations to be used in the conduct of this trial are provided on the Contact List.

11.0 REFERENCES

1. Walsh TJ, Raad I, Patterson TF, Chandrasekar P, Donowitz GR, Graybill R, et al. Treatment of invasive aspergillosis with posaconazole in patients who are refractory to or intolerant of conventional therapy: an externally controlled trial. Clin Infect Dis 2007;44:2-12.

2. Raad II, Graybill JR, Bustamante AB, Cornely OA, Gaona-Flores V, Afif C, et al. Safety of long-term oral posaconazole use in the treatment of refractory invasive fungal infections. Clin Infect Dis 2006;42:1726-34.

3. Negroni R, Tobón A, Bustamante B, Shikanai-Yasuda MA, Patino H, Restrepo A. Posaconazole treatment of refractory eumycetoma and chromoblastomycosis. Rev Inst Med Trop S Paulo 2005;47:339-46.

4. Anstead GM, Corcovan G, Lewis J, Berg D, Graybill JR. Refractory coccidioidomycosis treated with posaconazole. Clin Infect Dis 2005;40:1770-6.

5. Cornely OA, Maertens J, Winston DJ, Perfect J, Ullmann AJ, Walsh TJ, et al. Posaconazole vs. fluconazole or itraconazole prophylaxis in patients with neutropenia. N Engl J Med 2007;356:348-59.

6. Ullmann AJ, Lipton JH, Vesole DH, Chandrasekar P, Lanston A, Tarantolo SR, et al. Posaconazole or fluconazole for prophylaxis in severe graft-versus-host disease. N Engl J Med 2007;356:335-47.

7. Vazquez JA, Skiest DJ, Nieto L, Northland R, Sanne I, Gogate J, et al. A multicenter randomized trial evaluating posaconazole versus fluconazole for oropharyngeal candidiasis in subjects with HIV/AIDS. Clin Infect Dis 2006;42:1179-86.

8. Investigator's Brochure for SCH 56592, January 2013.

9. Krishna G, Sansone-Parsons A, Martinho M, Kantesaria B, Pedicone L. Posaconazole plasma concentrations in juvenile patients with invasive fungal infection. Antimicrob Agents Chemother 2007;51(3):812-8.

10. Wiley JM, Smith N, Leventhal M, Graham ML, Strauss LC, Hurwitz CA, et al. Invasive fungal disease in pediatric acute leukemia patients with fever and neutropenia during induction chemotherapy: a multivariate analysis of risk factors. J Clin Oncol 1990;8:280-6.

11. Rosen GP, Nielsen K, Glenn S, Abelson J, Deville J, Moore TB. Invasive fungal infections in pediatric oncology patients: 11-year experience at a single institution. J Pediatr Hematol Oncol 2005;27:135-40.

12. Castagnola E, Cesaro S, Giacchino M, Livadiotti S, Tucci F, Zanazzo G, et al. Fungal infections in children with cancer. A prospective, multicenter surveillance study. Ped Inf Dis J 2006;25:634-9.

13. Hovi L, Saxen H, Saarinen-Pihkala UM, Vettenranta K, Meri T, Richardson M. Prevention and monitoring of invasive fungal infections in pediatric patients with cancer and hematologic disorders. Pediatr Blood Cancer 2007;48:28-34.

14. Hovi L, Saarinen-Pihkala UM, Vettenranta K, Saxen H. Invasive fungal infections in pediatric bone marrow transplant recipients: single center experience of 10 years. Bone Marrow Transpl 2000;26:999-1004.

15. Dvorak CC, Steinbach WJ, Brown JM, Agarwal R. Risks and outcomes of invasive fungal infections in pediatric patients undergoing hematopoietic cell transplantation. Bone Marrow Transpl 2005;36:621-9.

16. Gibaldi M, Perrier D. Pharmacokinetics. 2nd ed. New York: Marcel Dekker, Inc., 1982:409-17.

17. TRPT-002545. Pharmaceutical Sciences Technical Report. Posaconazole oral suspension pediatric device dose recovery study report. Summit (NJ): Merck Sharp & Dohme Corp.; 2012 Dec.

Appendix 1 Specimen Handling and Shipping Instructions

Specimen shipping and handling will be done according to the instructions provided by the Sponsor or the Sponsor's designee.

Appendix 2 Posaconazole Oral Administration

The POS oral dosing will be administered using the Sponsor-supplied 1-mL, 3-mL, or 10-mL oral dosing syringe.

As this is an open label study, use one bottle for one subject from the bulk supply. The bulk supply will be packaged with 6 bottles per box. Each bottle will be used as an individual treatment bottle.

Posaconazole Oral Treatment Preparation and Administration

1. Take one bottle of POS oral suspension from the bulk supply. Select the bottle from the batch with the shortest expiration date. Inspect the bottle for any breakage or leakage. If either has occurred, record this on the TASIR form. Do not use this bottle and return it to the bulk supply. Immediately complete an RCQ Clinical Supply Complaint GCP Inquiry form and fax to the Sponsor.
2. Inspect all bottles in the box to ensure that POS has not come in contact with the other bottle labels. If the label of any bottle is contaminated and not readable, record this on the TASIR and do not use this bottle. Place an "X" or other identifier to note this unused bottle and return it to the shelf.
3. For an intact bottle, write the subject number, site number, date dispensed, bottle number, and amount to be administered on the bottle in the lines provided.
4. Remove the cap. Weigh the full bottle and record the weight to 2 decimal places (00.00) on the TAAL form. ***Note:*** *Weighing of the bottles is recommended but not required. If unable to follow this compliance/reconciliation check, then the site will need to discuss an alternate method with the site's local Sponsor monitor. The alternate method needs to be approved and documented in the site's materials and on the final study drug compliance/reconciliation documentation.*
5. Replace the cap and shake the bottle thoroughly to prevent normal separation of contents.
6. Remove the cap and place the bottle adapter snugly into the neck of the bottle.
7. Draw air into the dosing syringe and insert syringe into the bottle adapter.

   Invert the bottle, push air into the bottle, and draw the calculated dose of study drug (mL), into dosing syringe.

   Turn bottle upright and remove the dosing syringe.

   Remove the adapter, replace the bottle cap and place syringe tip onto syringe.

   Administer dose and discard syringes as per the hospital's infectious disease policy.

Notes:

- Subsequent doses:
  1. For each subsequent dose, draw the calculated dose of study drug (mL) into dosing syringe.
  2. Allow variation as per the medication administration schedule.
  3. Continue to shake bottle prior to each dose and fill the calculated dose (mL) into dosing syringe until bottle is empty or subject stops dosing.
- When bottle is empty or subject stops oral dosing:
  1. Remove the cap and weigh the bottle.
  2. Record the weight on the TAAL form. ***Note:*** *Weighing of the bottles is recommended but not required. If unable to follow this compliance/reconciliation check, then the site will need to discuss an alternate method with the site's local Sponsor monitor. The alternate method needs to be approved and documented in the site's materials and on the final study drug compliance/reconciliation documentation.*
  3. If subject will continue dosing, select another bottle from the bulk supply and repeat steps.
  4. Store all bottle (used and unused) for verification by the monitor.

Appendix 3 Defining Opportunistic Invasive Fungal Infections

(European Organization for Research and Treatment of Cancer/Invasive Fungal Infections Cooperative Group, National Institute of Allergy and Infectious Diseases Mycoses Study Group)

MAJOR ARTICLE

Defining Opportunistic Invasive Fungal Infections in Immunocompromised Patients with Cancer and Hematopoietic Stem Cell Transplants: An International Consensus

S. Ascioglu,[1] J. H. Rex,[2] B. de Pauw,[1] J. E. Bennett,[2] J. Bille,[1] F. Crokaert,[1] D. W. Denning,[1] J. P. Donnelly,[1] J. E. Edwards,[2] Z. Erjavec,[1] D. Fiere,[1] O. Lortholary,[1] J. Maertens,[1] J. F. Meis,[1] T. F. Patterson,[2] J. Ritter,[1] D. Selleslag,[1] P. M. Shah,[1] D. A. Stevens,[2] and T. J. Walsh,[2] on behalf of the Invasive Fungal Infections Cooperative Group of the European Organization for Research and Treatment of Cancer and Mycoses Study Group of the National Institute of Allergy and Infectious Diseases

[1]European Organization for Research and Treatment of Cancer/Invasive Fungal Infections Cooperative Group, Brussels; and [2]National Institute of Allergy and Infectious Diseases Mycoses Study Group, National Institutes of Health, Bethesda, Maryland

During the past several decades, there has been a steady increase in the frequency of opportunistic invasive fungal infections (IFIs) in immunocompromised patients. However, there is substantial controversy concerning optimal diagnostic criteria for these IFIs. Therefore, members of the European Organization for Research and Treatment of Cancer/Invasive Fungal Infections Cooperative Group and the National Institute of Allergy and Infectious Diseases Mycoses Study Group formed a consensus committee to develop standard definitions for IFIs for clinical research. On the basis of a review of literature and an international consensus, a set of research-oriented definitions for the IFIs most often seen and studied in immunocompromised patients with cancer is proposed. Three levels of probability are proposed: "proven," "probable," and "possible." The definitions are intended for use in the context of clinical and/or epidemiological research, not for clinical decision making.

Opportunistic invasive fungal infections (IFIs) are a major cause of morbidity and mortality in immunocompromised patients. However, there still remains much uncertainty and controversy regarding the best methods for establishing the diagnosis of most IFIs. Practicing physicians approach this uncertainty by treating suspected cases empirically, whereas those who review cases for research purposes tend to accept only cases in which the diagnosis is certain. This disparity of approaches is particularly apparent in the conduct of clinical trials designed to show that a new drug exhibits sufficient efficacy.

These difficulties are not unique to the study of IFIs, and wide practice variations are known to exist in all areas of medicine [1, 2]. The uncertainty in disease definition is thought to be a key contributor to these variations [1]. Strategies to minimize such uncertainties have resulted in movements such as evidence-based medicine [3] and practice guidelines [4]. In studies in which there is no assurance that homogeneous populations are being evaluated, the selection of study subjects may be biased and, therefore, their findings cannot be used to make generalizations about cause, epidemiology, prognosis, treatment, or prevention [5]. Typically, a set of characteristic abnormalities is used for diagnosis of dis-

Received 3 November 2000; revised 14 May 2001; electronically published 26 November 2001.

Financial support: Unrestricted educational grant from Pfizer (to S.A.); European Organization for Research and Treatment of Cancer/Invasive Fungal Infections Cooperative Group (to S.A.); Aronex, Janssen Research Foundation, Liposome Company, NeXstar-Gilead, and Pfizer.

Reprints or correspondence: Dr. Thomas J. Walsh, National Cancer Institute, 9000 Rockville Pike, Bldg. 10, Rm. 13N240, Bethesda, MD 20892 (walsht@mail.nih.gov).

Clinical Infectious Diseases 2002;34:7–14
© 2002 by the Infectious Diseases Society of America. All rights reserved.
1058-4838/2002/3401-0002$03.00

eases that do not have pathognomonic signs. Such classification and diagnostic criteria have proven to be extremely useful in areas that involve rheumatic diseases, endocarditis, and psychiatric diseases [6–8]. Also, subdivision by certainty of diagnosis is useful in many situations in which definite criteria cannot be applied to all cases [9]. A series of estimates of probability (e.g., definite, proven, suspected, presumptive, and probable) is also a part of all of these systems, which is also evident from the literature on IFIs [10]. Unfortunately, even these terms may take a range of meanings, and there is thus wide variation in their interpretation [11]. The resulting array of descriptive phrases makes it difficult to pool data from multiple centers and thus hinders drug development, impedes the pace of clinical research, and perpetuates confusion in the literature. Although there are reference standards for diagnosing IFIs, these usually involve use of invasive procedures to obtain tissue specimens for culture and histological examination. Unfortunately, these procedures are not always feasible. Therefore, clinicians caring for such patients may rely on a combination of less-specific clinical, laboratory, and radiological data. Indeed, situations that present significant diagnostic uncertainty are the norm for most IFIs, and diagnostic criteria with perfect sensitivity and specificity do not exist for any IFI or, indeed, for many other diseases [12–16].

In an effort to standardize the definitions of IFIs for clinical research, the Invasive Fungal Infections Cooperative Group (IFICG) of the European Organization for Research and Treatment of Cancer (EORTC) convened a committee of EORTC/IFICG members with the goal of defining and classifying the IFIs that are commonly seen and studied in immunocompromised patients with cancer. At a later stage, members of the Mycoses Study Group (MSG) of the National Institute of Allergy and Infectious Diseases (NIAID) subsequently joined this consensus effort. This document is the final result of the combined efforts of this consensus committee, on the basis of several rounds of discussion that culminated in the approval of the document.

The committee sought to develop definitions for clinical researchers that were based on published information, deemed clinically applicable by experienced researchers, and practical within the context of the types of objectively verifiable data generated in daily clinical practice. Of importance, these guidelines should not be taken as strict rules for making or excluding the diagnosis of an IFI in clinical settings.

METHODS

Defining the problem. A systematic review of the literature for an explicit identification of major problems related to heterogeneity of immunocompromised patients with cancer who have IFIs was undertaken and is described elsewhere [10]. *Pneumocystis* infections were not considered. In brief, the abstracts of 7086 articles published from 1985 through 1997 were screened. Of these, 173 articles were finally selected because they were reports exclusively regarding clinical research on immunocompromised patients with cancer or recipients of hematopoietic stem cell transplants who also had deep-tissue fungal infections. The minimum diagnostic criteria used to include patients in the study were extracted from definitions devised by the investigators. Likewise, the criteria used to express different degrees of diagnostic probability were summarized, as were the terms most often used to express these levels of uncertainty.

Construction and function of the consensus committee. The IFICG, 1 of the 16 cooperative groups of the EORTC, created a task force in March 1997 under the chairmanship of Dr. Ben E. de Pauw. This initial committee consisted of 12 members of the IFICG from 8 different countries. Members of NIAID MSG were asked to join this committee in 1998.

The committee began with the information from the literature review, and each committee member was asked to provide comments on the terms and the construction they preferred. These comments led to revisions of the draft document in a cycle that was repeated many times until a final consensus was reached. During this process, the consensus committee also met face-to-face twice to discuss the proposal. In addition, the proposal was discussed in 3 group meetings of EORTC/IFICG, where it was open to all members of IFICG for discussion.

RESULTS

The definitions. We propose definitions for a new classification based on the level of certainty for the diagnosis of IFIs (tables 1 and 2). This proposal includes both diagnostic criteria for proven IFIs and also classification criteria for probable and possible diseases that are intended to promote a more uniform description of the patients when various research endeavors are reported.

The committee chose the terms "proven," "probable," and "possible" to express disease certainty. Although other terms could be used, our literature review showed a clear trend among investigators favoring the use of these terms. These verbal expressions of subjective probability correspond to a reasonably accurate numerical estimate [12, 14]. Three elements form the basis of the proposed definitions: host factors, clinical manifestations, and mycological results.

Host factors. We restricted the scope of the definitions to patients with cancer, whether treated or not, and to recipients of hematopoietic stem cell transplants who were suspected of having an IFI. However, because these patients do not present a single uniform entity, additional host factors

Table 1. Definitions of invasive fungal infections in patients with cancer and recipients of hematopoietic stem cell transplants.

Category, type of infection	Description
Proven invasive fungal infections	
Deep tissue infections	
Molds[a]	Histopathologic or cytopathologic examination showing hyphae from needle aspiration or biopsy specimen with evidence of associated tissue damage (either microscopically or unequivocally by imaging); or positive culture result for a sample obtained by sterile procedure from normally sterile and clinically or radiologically abnormal site consistent with infection, excluding urine and mucous membranes
Yeasts[a]	Histopathologic or cytopathologic examination showing yeast cells (*Candida* species may also show pseudohyphae or true hyphae) from specimens of needle aspiration or biopsy excluding mucous membranes; or positive culture result on sample obtained by sterile procedure from normally sterile and clinically or radiologically abnormal site consistent with infection, excluding urine, sinuses, and mucous membranes; or microscopy (India ink, mucicarmine stain) or antigen positivity[b] for *Cryptococcus* species in CSF
Fungemia	
Molds[a]	Blood culture that yields fungi, excluding *Aspergillus* species and *Penicillium* species other than *Penicillium marneffei*, accompanied by temporally related clinical signs and symptoms compatible with relevant organism
Yeasts[a]	Blood culture that yields *Candida* species and other yeasts in patients with temporally related clinical signs and symptoms compatible with relevant organism
Endemic fungal infections[c]	
Systemic or confined to lungs	Must be proven by culture from site affected, in host with symptoms attributed to fungal infection; if culture results are negative or unattainable, histopathologic or direct microscopic demonstration of appropriate morphological forms is considered adequate for dimorphic fungi (*Blastomyces, Coccidioides* and *Paracoccidioides* species) having truly distinctive appearance; *Histoplasma capsulatum* variant *capsulatum* may resemble *Candida glabrata*
Disseminated	May be established by positive blood culture result or positive result for urine or serum antigen by means of RIA [17]
Probable invasive fungal infections	At least 1 host factor criterion (see table 2); and 1 microbiological criterion; and 1 major (or 2 minor) clinical criteria from abnormal site consistent with infection
Possible[d] invasive fungal infections	At least 1 host factor criterion; and 1 microbiological or 1 major (or 2 minor) clinical criteria from abnormal site consistent with infection

[a] Append identification at genus or species level from culture, if available.
[b] False-positive cryptococcal antigen reactions due to infection with *Trichosporon beigelii* [1], infection with *Stomatococcus mucilaginosis* [2], circulating rheumatoid factor [3], and concomitant malignancy [4] may occur and should be eliminated if positive antigen test is only positive result in this category.
[c] Histoplasmosis, blastomycosis, coccidioidomycosis, and paracoccidioidomycosis.
[d] This category is not recommended for use in clinical trials of antifungal agents but might be considered for studies of empirical treatment, epidemiological studies, and studies of health economics.

need to be considered when confronted with IFIs that cannot be proven. Those additional factors (table 2) reflect the literature and the opinions of the consensus committee. The criteria for proven IFIs are likely valid for all host groups, not just patients with cancer and recipients of hematopoietic stem cell transplants.

Mycological evidence. Mycological evidence begins with the specimen. Thus, specimens obtained from normally sterile but clinically abnormal sites were rated to be more reliable than were those obtained from adjacent normal sites or sites normally colonized with resident commensal flora. These specimens were considered necessary for proving IFIs. Hence, the mycological evidence acquired by means of either direct examination or culture of specimens from sites that may be colonized (e.g., sputum, bronchoalveolar lavage fluid, or sinus aspirate) were thought only to support the diagnosis, not prove it. Similarly, with the sole exception of *Cryptococcus neoformans,* indirect tests to detect antigen were considered to be suggestive but not conclusive. Thus, the nature and quality of the specimen and the use of direct and indirect mycological techniques were incorporated into each of the criteria.

Clinical features. An attempt was made to distinguish between evidence of abnormality of an organ or organ system that was consistent with an IFI from evidence that could be associated with another infective process. For example, evidence of abnormal appearance by radiological or other imaging was given a much higher rating than were other, less specific signs, such as pleural rub. Symptoms and some other clinical features

Table 2. Host factor, microbiological, and clinical criteria for invasive fungal infections in patients with cancer and recipients of hematopoietic stem cell transplants.

Type of criteria	Criteria
Host factors	Neutropenia (<500 neutrophils/mm^3 for >10 days)
	Persistent fever for >96 h refractory to appropriate broad-spectrum antibacterial treatment in high-risk patients
	Body temperature either >38°C or <36°C and any of the following predisposing conditions: prolonged neutropenia (>10 days) in previous 60 days, recent or current use of significant immunosuppressive agents in previous 30 days, proven or probable invasive fungal infection during previous episode of neutropenia, or coexistence of symptomatic AIDS
	Signs and symptoms indicating graft-versus-host disease, particularly severe (grade ≥2) or chronic extensive disease
	Prolonged (>3 weeks) use of corticosteroids in previous 60 days
Microbiological	Positive result of culture for mold (including *Aspergillus*, *Fusarium*, or *Scedosporium* species or Zygomycetes) or *Cryptococcus neoformans* or an endemic fungal pathogen[a] from sputum or bronchoalveolar lavage fluid samples
	Positive result of culture or findings of cytologic/direct microscopic evaluation for mold from sinus aspirate specimen
	Positive findings of cytologic/direct microscopic evaluation for mold or *Cryptococcus* species from sputum or bronchoalveolar lavage fluid samples
	Positive result for *Aspergillus* antigen in specimens of bronchoalveolar lavage fluid, CSF, or ≥2 blood samples
	Positive result for cryptococcal antigen in blood sample[b]
	Positive findings of cytologic or direct microscopic examination for fungal elements in sterile body fluid samples (e.g., *Cryptococcus* species in CSF)
	Positive result for *Histoplasma capsulatum* antigen in blood, urine, or CSF specimens [17]
	Two positive results of culture of urine samples for yeasts in absence of urinary catheter
	Candida casts in urine in absence of urinary catheter
	Positive result of blood culture for *Candida* species
Clinical	Must be related to site of microbiological criteria and temporally related to current episode
Lower respiratory tract infection	
Major	Any of the following new infiltrates on CT imaging: halo sign, air-crescent sign, or cavity within area of consolidation[c]
Minor	Symptoms of lower respiratory tract infection (cough, chest pain, hemoptysis, dyspnea); physical finding of pleural rub; any new infiltrate not fulfilling major criterion; pleural effusion
Sinonasal infection	
Major	Suggestive radiological evidence of invasive infection in sinuses (i.e., erosion of sinus walls or extension of infection to neighboring structures, extensive skull base destruction)
Minor	Upper respiratory symptoms (e.g., nasal discharge, stuffiness); nose ulceration or eschar of nasal mucosa or epistaxis; periorbital swelling; maxillary tenderness; black necrotic lesions or perforation of hard palate
CNS infection	
Major	Radiological evidence suggesting CNS infection (e.g., mastoiditis or other parameningeal foci, extradural empyema, intraparenchymal brain or spinal cord mass lesion)
Minor	Focal neurological symptoms and signs (including focal seizures, hemiparesis, and cranial nerve palsies); mental changes; meningeal irritation findings; abnormalities in CSF biochemistry and cell count (provided that CSF is negative for other pathogens by culture or microscopy and negative for malignant cells)
Disseminated fungal infection	Papular or nodular skin lesions without any other explanation; intraocular findings suggestive of hematogenous fungal chorioretinitis or endophthalmitis
Chronic disseminated candidiasis	Small, peripheral, targetlike abscesses (bull's-eye lesions) in liver and/or spleen demonstrated by CT, MRI, or ultrasound, as well as elevated serum alkaline phosphatase level; supporting microbiological criteria are not required for probable category
Candidemia	Clinical criteria are not required for probable candidemia; there is no definition for possible candidemia

[a] *H. capsulatum* variant *capsulatum*, *Blastomyces dermatitidis*, *Coccidioides immitis*, or *Paracoccidioides brasiliensis*.
[b] See table 1 footnote *b* for causes of false-positive reactions that must be considered and eliminated from consideration.
[c] In absence of infection by organisms that may lead to similar radiological findings including cavitation, such as *Mycobacterium*, *Legionella*, and *Nocardia* species.

were also regarded as less specific and only supportive. Thus, 2 levels of evidence (major and minor) were incorporated into the concept of clinical features of IFIs.

DISCUSSION

The consensus for many of the definitions was determined after extensive debate. In many cases, that debate is instructive and elements of it are reviewed here.

Proven category of infections. The "proven" category consists of criteria that allow IFIs to be diagnosed with certainty and that differentiates between deep-tissue infections and fungemia. There was general agreement among committee members that the highest level of certainty in diagnosing an invasive fungal infectious disease is attained by establishing the presence of fungi in tissue by biopsy or a needle aspirate. However, the committee also agreed that demonstration of infection either by culture or histological examination was sufficient to be able to distinguish molds from yeasts and that both, although desirable, were not strictly necessary.

A branching septate mold in tissue is most commonly *Aspergillus* species. However, other organisms, including hyaline and dematiaceous molds, may be morphologically indistinguishable from *Aspergillus* species. A Fontana-Masson stain may help to distinguish hyaline from dematiaceous organisms. By comparison, the histological appearance of the ribbon-like broad coenocytic (sparsely septated) hyphal structures of Zygomycetes is usually readily distinguishable from *Aspergillus* species and other septated molds. The histological appearance of the endemic dimorphic fungi—*Histoplasma capsulatum,* as small intracellular budding yeasts; *Coccidioides immitis,* as spherules; *Paracoccidioides brasiliensis,* as large yeasts with multiple daughter yeasts in a "pilot wheel configuration"; and *Blastomyces dermatitidis,* as thick-walled broad-based budding yeasts—is sufficiently distinctive as to permit a definitive diagnosis as proven fungal infection caused by 1 of these pathogens. Whenever culture is possible, a specific diagnosis to the species level should be provided.

We propose that either typical microscopic findings or the detection of antigen in CSF be accepted as diagnostic for cryptococcosis in an immunocompromised host in the appropriate clinical setting [18]. It is, however, important to be aware of the rare but definite causes of a false-positive cryptococcal antigen result, including infection caused by *Trichosporon* species [19], infection with *Stomatococcus mucilaginosis* [20], presence of circulating rheumatoid factor [21], and concomitant malignancy [22].

For the purposes of these definitions, the committee preferred the term "fungemia" to "bloodstream fungal infections," because this avoids the impression that isolating a fungus from culture of blood signifies an infection that is confined to the bloodstream. For *Fusarium* species and *Penicillium marneffei,* fungemia, more likely than not, represents deep-tissue infection. Culture of *Aspergillus* species and other *Penicillium* species from the blood may represent serious disease but is more likely to represent specimen contamination; therefore, it is not taken as proof of diseases for purposes of clinical research.

Probable and possible categories of infection. Patients in these categories present enough information suggesting an IFI to warrant some form of empirical antifungal therapy. These patients are frequently characterized by being febrile, despite receipt of broad-spectrum antibiotics, and they may have a potential focus of infection. A definitive tissue diagnosis for radiologically demonstrable lesions, if present, is not considered feasible. Many independent reviewers would dismiss these cases for lack of convincing evidence. The problem of uncertainty cannot be understated or disregarded as if it does not exist, because both clinicians and researchers are regularly confronted with it. Rather, we sought to incorporate uncertainty into our quest for diagnosis and translate it into probabilities as accurately as possible [15, 16]. For a case of IFI to be considered "probable," each of the 3 elements of host factor, clinical features, and mycological evidence has to be present [23]. By contrast, a patient who has at least 1 criterion from the host factors category but who does not have clinical features or mycological evidence has a case that can be classified only as "possible." Because this is the least specific category, but one often used in clinical practice to treat patients empirically, we do not recommend its use in clinical trials of antifungal agents. Rather, it might be considered in studies of empirical treatment, epidemiological studies, and studies of health economics.

Aspergillosis. Because culture has such a poor sensitivity in the diagnosis of invasive aspergillosis, reliance on culture alone results in substantial underdiagnosis. On the other hand, cultures that yield *Aspergillus* species do not always reflect invasive disease, because colonization can occur in immunocompromised patients, and false-positive results that result from environmental contamination are occasionally a problem. Thus, the committee strongly supported the concept of a proven mold infection on the basis of the findings of histopathology or microscopy without necessarily requiring culture confirmation. The committee also proposed use of *Aspergillus* antigen testing as a finding that would support a probable diagnosis. The detection of *Aspergillus* antigenemia has been shown in experimental assays to correlate with clinical diagnosis and response to antifungal therapy [24, 25]. However, the clinical utility of these assays has been limited, in part because of their lack of widespread availability. Recently, a sandwich ELISA technique that uses a monoclonal antibody to galactomannan has been developed [26]. This assay (licensed by Sanofi Diagnostics Pasteur to Bio-Rad Laboratories) has been used most extensively in western Europe. Recent prospective studies of

hematology patients have demonstrated a sensitivity and specificity of >90% with this method [27]. Of note, there have been false-positive reactions, with recommendations from the manufacturer to consider the test a true-positive result only when >1 sample is positive. The use of PCR to detect invasive fungal pathogens, including *Aspergillus* species, has been reported, but false-positive results can occur, and a standardized commercial method is not available [28]. Thus, the committee decided that at the present time, the routine use of PCR in the diagnosis of invasive aspergillosis cannot be recommended.

Candidemia. Although other presentations are possible and are recognized by the definitions, candidemia is taken as a key sign of disseminated candidiasis. However, significant controversy surrounds the interpretation of positive blood culture results for candidemia [29]. In reviewing these controversies, the committee recognized that not all groups of patients with candidemia have the same risk of clinically significant widespread dissemination. The principal classification factor is the presence or absence of neutropenia. Patients with neutropenia have a much higher rate of provable visceral dissemination and a much higher mortality rate [30]. Although similar diagnostic criteria are applied to the 2 groups, patients with and without neutropenia cannot rationally be aggregated for purposes of data analysis. Also of note, candidemia, rather than being too nonspecific, is instead a marker (although insensitive) of deeply invasive candidiasis—that is, culture of blood samples often yields negative results in the presence of deep visceral candidiasis [31].

The second major subdivision of patients with candidemia revolves around the presence or absence of a central venous catheter. Broad dismissal of positive results of culture of blood from patients who have a central venous catheter in place is inappropriate. However, it is certainly possible that specimens drawn through a catheter would become contaminated during the collection process. The committee considered addressing this with requirements for specific numbers of blood cultures but thought that this was impractical. Although increasing numbers of positive blood culture results have been correlated with the likelihood of significant invasion [32], final blood culture data may not exist at the time a patient is considered for eligibility in a trial. In a related problem, the relevance of the site from which the culture-positive blood specimen was obtained was extensively discussed. Cultures obtained via peripheral venipuncture or through a newly inserted catheter would seem preferable on the general grounds that the catheters, rather than the patient, may be the cause of a culture-positive specimen. However, it is also clear that catheters may represent a major intravascular nidus of infection and that sampling via the catheter may provide early indication of a problem that has yet to reach a stage at which it can be detected in a more dilute specimen obtained from a remote site. In addition, records on the precise site at which a blood specimen was obtained are not consistently available or accurate. A comprehensive review of 155 episodes of catheter-related candidemia in patients with cancer found that, regardless of whether blood cultures were obtained from a central catheter or peripheral site, the frequency of autopsy-proven candidiasis was the same [32]. Thus, the committee elected to establish no specific requirements for the site from which the specimen was obtained. To resolve all of these challenges, heavy reliance was placed on the requirement for concomitant signs of infection.

Patients with candidemia who have temporally related signs of infection are always considered to have a significant, proven infection. Given that the signs and symptoms are incorporated into the definition of proven candidemia, the category of probable candidemia relies on host factors and microbiological factors. Patients with neutropenia, patients with graft-versus-host disease, or patients receiving corticosteroids who have candidemia without signs of infection are considered to have probable disease. But, for example, patients who have neutropenia and candidemia, who do not have signs of infection, and who do not also meet ⩾1 of the other "host factors" criteria are considered to have a disease process that cannot reasonably be studied within the guidelines of this document. Although such patients should probably still receive some form of therapy [33], their relevance to a clinical trial is uncertain. The committee decided not to incorporate and define a "possible" category for candidemia. The definition for chronic disseminated candidiasis (hepatosplenic candidiasis) likewise required a modification because of the infrequency of microbiological support for this condition. For this diagnosis, the committee decided not to define a "possible" category, and for chronic disseminated candidiasis only, a microbiological criterion is not required for a "probable" diagnosis in a suitable host.

Other forms of invasive candidiasis. Other forms of invasive candidiasis are handled in 2 ways. First, any situation in which a biopsy of a normally sterile site shows *Candida* species by culture or histopathologic examination will qualify as proven candidiasis. Because of the many possible permutations (e.g., candidemia, candidemia with hepatosplenic involvement, hepatosplenic involvement without candidemia), the definitions do not specifically contain a list of possible relevant categories. Rather, these are all grouped under the general title of "proven invasive candidiasis."

More challenging are the scenarios in which *Candida* species are isolated from such specimens as urine, sputum, or wound drainage. A very conservative approach was taken here. Although a rational physician might well chose to give antifungal therapy to a patient with fever and *Candida* species in the urine (or sputum, or wound drainage), this scenario is simply too ill-defined for study in a clinical trial.

Comparison with earlier NIAID MSG definitions. The

proposed definitions in this manuscript differ from the earlier NIAID MSG definitions by focusing on the specific issues of oncology and hematopoietic stem cell transplantation. They recognize that the interplay of host factors and clinical manifestations may enhance the diagnostic probability of an IFI. The committee recognized that not all patients with neutropenia have the same risk for development of IFIs [34]. For example, recovering *Aspergillus* species from the respiratory secretions of a patient with profound neutropenia who has acute leukemia or a patient receiving high dosages of corticosteroids carries much more significance for the development of invasive aspergillosis than it does when *Aspergillus* species are recovered from a patient with lung cancer and transient immunosuppression [35]. The new system now also incorporates the use of galactomannan antigen in defining invasive aspergillosis.

Limitations of these definitions for clinical practice. These definitions should not be used to guide clinical practice. There are frequent clinical situations in the "possible" category in which therapy is warranted on empirical grounds. The objective of this project was to develop definitions that would identify reasonably homogeneous groups of patients for clinical research, as well as to foster international collaboration, design of clinical trials, and interpretation of new therapeutic interventions for management of IFIs in patients receiving cancer chemotherapy and those undergoing hematopoietic stem cell transplantation.

Acknowledgments

We acknowledge the contributions of P. Ljungman, M. Nucci, J. E. Patterson, G. Petrikkos, M. A. Pfaller, M. G. Rinaldi, and C. Viscoli, who took part in the consensus committee meetings or sent us comments.

References

1. Eddy DM. Probabilistic reasoning in clinical medicine: problems and opportunities. In: Kahneman D, Slovic P, Tverksy A, eds. Judgement under uncertainty: heuristics and biases. New York: Cambridge University Press, **1982**:249–67.
2. Chassin MR, Kosecoff J, Park RE, et al. Does inappropriate use explains geographic variations in the use of health care services? A study of three procedures. JAMA **1987**; 258:2533–7.
3. Sackett DL, Rosenberg WM, Gray JA, Haynes RB, Richardson WS. Evidence based medicine: what it is and what it isn't. BMJ **1996**; 312: 71–2.
4. Woolf SH, Grol R, Hutchinson A, Eccles M, Grimshaw J. Potential benefits, limitations, and harms of clinical guidelines. BMJ **1999**; 318: 527–30.
5. Hyams KC. Developing case definitions for symptom-based conditions: the problem of specificity. Epidemiol Rev **1998**; 20:148–56.
6. International Study Group for Behçet's Disease. Evaluation of diagnostic ('classification') criteria in Behçet's disease—towards internationally agreed criteria. Br J Rheumatol **1992**; 31:299–328.
7. Hunder GG, Arend WP, Bloch DA, et al. The American College of Rheumatology 1990 criteria for the classification of vasculitis. Arthritis Rheum **1990**; 33:1065–7.
8. Bayer AS, Ward JI, Ginzton LE, Shapiro SM. Evaluation of new clinical criteria for the diagnosis of infective endocarditis. Am J Med **1994**; 96: 211–9.
9. Kelsey JL, Whittemore AS, Evans AS, Thompson WD. Prospective cohort studies: planning and execution. In: Methods in observational epidemiology, 2nd ed. New York: Oxford University Press, **1996**: 87–112.
10. Ascioglu S, de Pauw BE, Donnelly JP, Collette L. Reliability of clinical research on invasive fungal infections: a systematic review of the literature. Med Mycol **2001**; 39:35–40.
11. Wallsten TS, Budescu DV, Rapoport A, Zwick R, Forsyth B. Measuring the vague meanings of probability terms. J Exp Psychol Gen **1986**; 115: 348–65.
12. White E. The effect of misclassification of disease status in follow-up studies: implications for selecting disease classification criteria. Am J Epidemiol **1986**; 124:816–25.
13. Hunder GG. The use and misuse of classification and diagnostic criteria for complex diseases. Ann Intern Med **1998**; 129:417–8.
14. Bryant G, Norman GR. Expressions of probability: words and numbers [letter]. N Engl J Med **1980**; 302:411.
15. Kassirer JP. Our stubborn quest for diagnostic certainty. N Engl J Med **1989**; 320:1489–91.
16. Moskowitz AJ, Kuipers BJ, Kassier JP. Dealing with uncertainty, risks, and tradeoffs in clinical decisions: a cognitive science approach. Ann Intern Med **1988**; 108:435–49.
17. Durkin MM, Connolly PA, Wheat LJ. Comparison of radioimmunoassay and enzyme-linked immunoassay methods for detection of *Histoplasma capsulatum* var. *capsulatum* antigen. J Clin Microbiol **1997**; 35:2252–5.
18. Walsh TJ, Chanock SJ. Diagnosis of invasive fungal infections: advances in nonculture systems. Curr Clin Top Infect Dis **1998**; 18:101–53.
19. Walsh TJ, Melcher GP, Lee JW, Pizzo PA. Infections due to *Trichosporon* species: new concepts in mycology, pathogenesis, diagnosis, and treatment. Curr Top Med Mycol **1993**; 5:79–113.
20. Chanock SJ, Toltzis P, Wilson C. Cross-reactivity between *Stomatococcus mucilaginosus* and latex agglutination for cryptococcal antigen [letter]. Lancet **1993**; 342:1119–20.
21. Engler HD, Shea YR. Effect of potential interference factors on performance of enzyme immunoassay and latex agglutination assay for cryptococcal antigen. J Clin Microbiol **1994**; 32:2307–8.
22. Hopfer RL, Perry EV, Fainstein V. Diagnostic value of cryptococcal antigen in the cerebrospinal fluid of patients with malignant disease. J Infect Dis **1982**; 145:915.
23. Bloch DA, Moses LE, Michel BA. Statistical approaches to classification. Arthritis Rheum **1990**; 33:1137–44.
24. Patterson JE, Zidouh A, Miniter P, Andriole VT, Patterson TF. Hospital epidemiologic surveillance for invasive aspergillosis: patient demographics and the utility of antigen detection. Infect Control Hosp Epidemiol **1997**; 18:104–8.
25. Francis P, Lee JW, Hoffman A, et al. Efficacy of unilamellar liposomal amphotericin B in treatment of pulmonary aspergillosis in persistently granulocytopenic rabbits: the potential role of bronchoalveolar lavage D-mannitol and galactomannan as markers of infection. J Infect Dis **1994**; 169:356–68.
26. Verweij PE, Poulain D, Obayashi T, Patterson TF, Denning DW, Ponton J. Current trends in the detection of antigenaemia, metabolites, and cell wall markers for the diagnosis and therapeutic monitoring of fungal infections. Med Mycol **1998**; 36(Suppl 1):146–55.
27. Maertens J, Verhaegen J, Demuynck H, et al. Autopsy-controlled prospective evaluation of serial screening for circulating galactomannan by a sandwich enzyme-linked immunosorbent assay for hematological patients at risk for invasive aspergillosis. J Clin Microbiol **1999**; 37: 3223–8.
28. Einsele H, Hebart H, Roller G, et al. Detection and identification of

fungal pathogens in blood by using molecular probes. J Clin Microbiol **1997**; 35:1353–60.

29. Fraser VJ, Jones M, Dunkel J, Storfer S, Medoff G, Dunagan WC. Candidemia in a tertiary care hospital: epidemiology, risk factors, and predictors of mortality. Clin Infect Dis **1992**; 15:414–21.
30. Anaissie EJ, Rex JH, Uzun Ö, Vartivarian S. Predictors of adverse outcome in cancer patients with candidemia. Am J Med **1998**; 104: 238–45.
31. Berenguer J, Buck M, Witebsky F, Stock F, Pizzo PA, Walsh TJ. Lysis-centrifugation blood cultures in the detection of tissue-proven invasive candidiasis: disseminated versus single organ infection. Diagn Microbiol Infect Dis **1993**; 17:103–9.
32. Lecciones JA, Lee JW, Navarro EE, et al. Vascular catheter–associated fungemia in patients with cancer: analysis of 155 episodes. Clin Infect Dis **1992**; 14:875–83.
33. Edwards JE. Editorial response: should all patients with candidemia be treated with antifungal agents? Clin Infect Dis **1992**; 15:422–3.
34. Walsh TJ, Hiemenz J, Pizzo PA. Evolving risk factors for invasive fungal infections: all neutropenic patients are not the same [editorial]. Clin Infect Dis **1994**; 18:793–8.
35. Yu VL, Muder RR, Poorsatttar A. Significance of isolation of *Aspergillus* from the respiratory tract in diagnosis of invasive pulmonary aspergillosis: results of a three-year prospective study. Am J Med **1986**; 81: 249–54.

Appendix 4 Summary of Safety and POS Preliminary Pharmacokinetic Data from P03579

As of 25-Jan-2013, a total of 81 subjects, 2 to <18 years of age, were enrolled and treated in the study P03579. A review of the safety and PK data from the first two dose groups was performed by the external Data Monitoring Committee and the Sponsor. No safety concerns were identified. The POS PK exposure target for the study, ~90% of subjects with POS steady-state C_{avg} in the range of 500 ng/mL to 2500 ng/mL has not been achieved at either dose level. Summaries of the PK and safety data are provided in the below tables.

The preliminary PK results of POS plasma concentrations in P03579 subjects from Age Group 1 (2 to <7 years of age) Dose Groups 1 and 2, and Age Group 2 (7 to <18 years of age) Dose Groups 1 and 2, are shown in Table 5.

Table 5 A Summary of POS Plasma Concentrations in Pediatric Subjects

Protocol No. P03579

Dose (mg/kg/day, divided BID)	Age Range (yr)	N	Mean C_{avg} (%CV) (ng/mL)	Range C_{avg} (ng/mL)	Subjects (n/N %) with C_{avg} 500 ng/mL-<2500 ng/mL	Subjects (n/N %) with C_{avg} ≥500 ng/mL
12	2 -< 7	11	749 (121)	34.6 - 3350	36% (4/11)	45% (5/11)
	7 -< 18	14	1050 (76)	65.6 - 2420	64% (9/14)	64% (9/14)
18	2 -< 7	6	542 (57)	48.3 - 903	67% (4/6)	67% (4/6)
	7 -< 18	12	1240 (113)	182 - 4660	50% (6/12)	67% (8/12)

BID= twice a day; C_{avg}=AUCtf/tf; CV=coefficient of variation, expressed as a percent (%); n=number of subjects achieving the PK target; N=total number of subjects; PK=pharmacokinetic; tf=time of last quantifiable concentration within a dosing interval; yr=years.

Note: Data were summarized based on all subjects from the dose group of 12 mg/kg/day and Day-7 PK evaluable subjects from the dose group of 18 mg/kg/day.

Source Data: P03579 data on file. Kenilworth (NJ): Merck Sharp & Dohme Corp.; 2013 Feb.

An overview of the available safety data (through 25-Jan-2013) from P03579 is provided in Table 6.

Table 6 Summary of Adverse Events by Category, All Treated Subject

Protocol No. P03579

	Number (%) of Subjects				
	Dose Group 1 (12 mg/kg/ day BID) /Age Group 1 (2 yrs-<7 yrs) n=22	Dose Group 1 (12 mg/kg/ day BID) /Age Group 2 (7 yrs-<18 yrs) n=21	Dose Group 2 (18 mg/kg/ day BID) /Age Group 1 (2 yrs-<7 yrs) n=10	Dose Group 2 (18 mg/kg/ day BID) /Age Group 2 (7 yrs-<18 yrs) n=28	Total n=81
Category					
Treatment Emergent AE	21 (95)	21 (100)	9 (90)	26 (93)	77 (95)
Treatment-Related Treatment Emergent AE	11 (50)	5 (24)	3 (30)	16 (57)	35 (43)
Serious AE	5 (23)	3 (14)	1 (10)	7 (25)	16 (20)
Death	0	0	1 (10)	1 (4)	2 (2)
Severe/Life-Threatening Treatment Emergent AE	14 (64)	10 (48)	3 (30)	16 (57)	43 (53)
Study Drug Discontinuation due to AE	7 (32)	9 (43)	1 (10)	8 (29)	25 (31)

AE=Adverse Event; BID= twice a day; yrs=years of age.

Note: Deaths are also included in Serious AE count.

Source Data: P03579 data on file. Kenilworth (NJ): Merck Sharp & Dohme Corp.; 2013 Mar.

The Medical Dictionary for Regulatory Activities (MedDRA), version 15.1, was used for AE coding. AEs were summarized by MedDRA System Organ Class (SOC) and preferred term. Based on available safety data (through 25-Jan-2013), a summary of TEAEs organized by MedDRA SOC is provided for P03579 in Table 7. The most frequently (>20% of subjects) reported TEAEs were pyrexia, vomiting, thrombocytopenia, mucosal inflammation, anemia, and nausea.

Table 7 Summary of Treatment-Emergent Adverse Events, All Treated Subject

Protocol No. P03579

System Organ Class<br>Preferred Term	Number (%) of Subjects									
	Dose Group 1 (12 mg/kg/ day BID) /Age Group 1 (2 yrs-<7 yrs) n=22		Dose Group 1 (12 mg/kg/ day BID) /Age Group 2 (7 yrs-<18 yrs) n=21		Dose Group 2 (18 mg/kg/ day BID) /Age Group 1 (2 yrs-<7 yrs) n=10		Dose Group 2 (18 mg/kg/ day BID) /Age Group 2 (7 yrs-<18 yrs) n=28		Total n=81	
Subjects Reporting Any Adverse Event	21	(95)	21	(100)	9	(90)	26	(93)	77	(95)
Blood And Lymphatic System Disorders	10	(45)	9	(43)	1	(10)	14	(50)	34	(42)
Anaemia	5	(23)	3	(14)	1	(10)	8	(29)	17	(21)
Coagulopathy	0		0		0		2	(7)	2	(2)
Febrile Neutropenia	2	(9)	4	(19)	0		7	(25)	13	(16)
Granulocytopenia	0		0		0		1	(4)	1	(1)
Leukopenia	5	(23)	0		0		1	(4)	6	(7)
Lymphopenia	2	(9)	0		0		0		2	(2)
Neutropenia	3	(14)	0		0		2	(7)	5	(6)
Thrombocytopenia	8	(36)	7	(33)	1	(10)	7	(25)	23	(28)
Cardiac Disorders	4	(18)	3	(14)	1	(10)	3	(11)	11	(14)
Bradycardia	0		1	(5)	0		0		1	(1)
Pericardial Effusion	0		1	(5)	0		0		1	(1)
Sinus Tachycardia	1	(5)	0		0		0		1	(1)
Tachycardia	3	(14)	2	(10)	1	(10)	2	(7)	8	(10)
Ventricular Extrasystoles	0		0		0		1	(4)	1	(1)
Ear And Labyrinth Disorders	1	(5)	1	(5)	0		0		2	(2)

Table 7 Summary of Treatment-Emergent Adverse Events, All Treated Subject

Protocol No. P03579

System Organ Class<br>Preferred Term	Number (%) of Subjects<br>Dose Group 1 (12 mg/kg/ day BID) /Age Group 1 (2 yrs-<7 yrs)<br>n=22		Dose Group 1 (12 mg/kg/ day BID) /Age Group 2 (7 yrs-<18 yrs)<br>n=21		Dose Group 2 (18 mg/kg/ day BID) /Age Group 1 (2 yrs-<7 yrs)<br>n=10		Dose Group 2 (18 mg/kg/ day BID) /Age Group 2 (7 yrs-<18 yrs)<br>n=28		Total<br>n=81	
Ear Pain	1	(5)	1	(5)	0		0		2	(2)
Eye Disorders	2	(9)	3	(14)	2	(20)	0		7	(9)
Conjunctival Haemorrhage	0		1	(5)	0		0		1	(1)
Dry Eye	0		0		1	(10)	0		1	(1)
Eye Pain	0		1	(5)	0		0		1	(1)
Eye Pruritus	1	(5)	0		2	(20)	0		3	(4)
Eye Swelling	1	(5)	0		0		0		1	(1)
Photophobia	0		0		1	(10)	0		1	(1)
Pupillary Reflex Impaired	0		1	(5)	0		0		1	(1)
Gastrointestinal Disorders	16	(73)	14	(67)	4	(40)	18	(64)	52	(64)
Abdominal Discomfort	0		0		0		1	(4)	1	(1)
Abdominal Pain	7	(32)	6	(29)	0		2	(7)	15	(19)
Abdominal Pain Upper	2	(9)	0		0		1	(4)	3	(4)
Abdominal Tenderness	0		0		0		1	(4)	1	(1)
Anal Fissure	0		1	(5)	0		1	(4)	2	(2)
Anal Pruritus	1	(5)	0		0		0		1	(1)
Aphthous Stomatitis	0		1	(5)	0		0		1	(1)
Chapped Lips	0		1	(5)	0		1	(4)	2	(2)
Constipation	0		4	(19)	0		1	(4)	5	(6)

Table 7 Summary of Treatment-Emergent Adverse Events, All Treated Subject

Protocol No. P03579

System Organ Class<br>Preferred Term	Number (%) of Subjects									
	Dose Group 1 (12 mg/kg/ day BID) /Age Group 1 (2 yrs-<7 yrs)		Dose Group 1 (12 mg/kg/ day BID) /Age Group 2 (7 yrs-<18 yrs)		Dose Group 2 (18 mg/kg/ day BID) /Age Group 1 (2 yrs-<7 yrs)		Dose Group 2 (18 mg/kg/ day BID) /Age Group 2 (7 yrs-<18 yrs)		Total	
	n=22		n=21		n=10		n=28		n=81	
Diarrhoea	5	(23)	3	(14)	0		6	(21)	14	(17)
Dry Mouth	1	(5)	0		0		0		1	(1)
Enteritis	0		0		1	(10)	0		1	(1)
Flatulence	0		0		1	(10)	0		1	(1)
Gastrooesophageal Reflux Disease	0		1	(5)	0		1	(4)	2	(2)
Haematemesis	1	(5)	0		0		0		1	(1)
Lip Dry	0		0		1	(10)	0		1	(1)
Nausea	5	(23)	3	(14)	0		9	(32)	17	(21)
Oral Pain	0		2	(10)	0		0		2	(2)
Proctalgia	1	(5)	0		0		0		1	(1)
Stomatitis	3	(14)	0		2	(20)	4	(14)	9	(11)
Tongue Coated	1	(5)	1	(5)	0		0		2	(2)
Upper Gastrointestinal Haemorrhage	1	(5)	0		0		0		1	(1)
Vomiting	8	(36)	3	(14)	2	(20)	11	(39)	24	(30)
General Disorders And Administration Site Conditions	18	(82)	14	(67)	1	(10)	19	(68)	52	(64)
Catheter Site Discharge	0		1	(5)	0		0		1	(1)
Catheter Site Erythema	2	(9)	1	(5)	0		1	(4)	4	(5)
Catheter Site Haematoma	0		1	(5)	0		0		1	(1)
Catheter Site Haemorrhage	0		1	(5)	0		0		1	(1)

Table 7 Summary of Treatment-Emergent Adverse Events, All Treated Subject

Protocol No. P03579

	Number (%) of Subjects									
System Organ Class	Dose Group 1 (12 mg/kg/ day BID) /Age Group 1 (2 yrs-<7 yrs)		Dose Group 1 (12 mg/kg/ day BID) /Age Group 2 (7 yrs-<18 yrs)		Dose Group 2 (18 mg/kg/ day BID) /Age Group 1 (2 yrs-<7 yrs)		Dose Group 2 (18 mg/kg/ day BID) /Age Group 2 (7 yrs-<18 yrs)		Total	
Preferred Term	n=22		n=21		n=10		n=28		n=81	
Catheter Site Pain	1	(5)	1	(5)	0		0		2	(2)
Chest Pain	1	(5)	0		0		0		1	(1)
Chills	1	(5)	1	(5)	0		1	(4)	3	(4)
Device Occlusion	0		1	(5)	0		2	(7)	3	(4)
Fatigue	1	(5)	0		0		3	(11)	4	(5)
Local Swelling	1	(5)	0		0		0		1	(1)
Mass	0		1	(5)	0		0		1	(1)
Mucosal Inflammation	9	(41)	3	(14)	1	(10)	8	(29)	21	(26)
Multi-Organ Failure	0		0		0		1	(4)	1	(1)
Oedema	0		0		0		2	(7)	2	(2)
Oedema Peripheral	1	(5)	0		0		1	(4)	2	(2)
Pain	0		0		1	(10)	2	(7)	3	(4)
Pyrexia	14	(64)	8	(38)	1	(10)	8	(29)	31	(38)
Hepatobiliary Disorders	0		1	(5)	1	(10)	2	(7)	4	(5)
Hepatic Vein Occlusion	0		0		0		1	(4)	1	(1)
Hepatobiliary Disease	0		0		1	(10)	0		1	(1)
Hepatomegaly	0		1	(5)	0		0		1	(1)
Hyperbilirubinaemia	0		0		0		1	(4)	1	(1)
Venoocclusive Liver Disease	0		0		1	(10)	0		1	(1)

Table 7 Summary of Treatment-Emergent Adverse Events, All Treated Subject

Protocol No. P03579

System Organ Class / Preferred Term	Number (%) of Subjects									
	Dose Group 1 (12 mg/kg/ day BID) /Age Group 1 (2 yrs-<7 yrs) n=22		Dose Group 1 (12 mg/kg/ day BID) /Age Group 2 (7 yrs-<18 yrs) n=21		Dose Group 2 (18 mg/kg/ day BID) /Age Group 1 (2 yrs-<7 yrs) n=10		Dose Group 2 (18 mg/kg/ day BID) /Age Group 2 (7 yrs-<18 yrs) n=28		Total n=81	
Immune System Disorders	1	(5)	1	(5)	0		0		2	(2)
Anaphylactic Reaction	0		1	(5)	0		0		1	(1)
Drug Hypersensitivity	1	(5)	0		0		0		1	(1)
Infections And Infestations	6	(27)	8	(38)	4	(40)	6	(21)	24	(30)
Alpha Haemolytic Streptococcal Infection	0		2	(10)	0		0		2	(2)
Bacteraemia	0		1	(5)	0		1	(4)	2	(2)
Catheter Site Cellulitis	1	(5)	0		0		0		1	(1)
Cellulitis	0		0		1	(10)	0		1	(1)
Central Nervous System Infection	0		0		0		1	(4)	1	(1)
Clostridium Difficile Colitis	1	(5)	0		0		0		1	(1)
Fungal Infection	0		0		0		1	(4)	1	(1)
Gastroenteritis Norovirus	0		0		0		1	(4)	1	(1)
Kidney Infection	1	(5)	0		0		0		1	(1)
Klebsiella Sepsis	0		0		0		1	(4)	1	(1)
Localised Infection	0		1	(5)	0		0		1	(1)
Lung Infection	0		1	(5)	0		0		1	(1)
Mucosal Infection	1	(5)	0		0		0		1	(1)
Oral Candidiasis	0		1	(5)	0		0		1	(1)
Oral Herpes	0		0		0		1	(4)	1	(1)

Table 7 Summary of Treatment-Emergent Adverse Events, All Treated Subject

Protocol No. P03579

	Number (%) of Subjects									
System Organ Class	Dose Group 1 (12 mg/kg/ day BID) /Age Group 1 (2 yrs-<7 yrs)		Dose Group 1 (12 mg/kg/ day BID) /Age Group 2 (7 yrs-<18 yrs)		Dose Group 2 (18 mg/kg/ day BID) /Age Group 1 (2 yrs-<7 yrs)		Dose Group 2 (18 mg/kg/ day BID) /Age Group 2 (7 yrs-<18 yrs)		Total	
Preferred Term	n=22		n=21		n=10		n=28		n=81	
Pneumonia Klebsiella	0		0		0		1	(4)	1	(1)
Puncture Site Infection	1	(5)	0		0		0		1	(1)
Rhinitis	1	(5)	0		0		0		1	(1)
Sepsis	0		1	(5)	0		1	(4)	2	(2)
Sinusitis	0		0		1	(10)	0		1	(1)
Staphylococcal Sepsis	0		0		0		1	(4)	1	(1)
Staphylococcal Skin Infection	0		1	(5)	0		0		1	(1)
Streptococcal Bacteraemia	0		1	(5)	0		0		1	(1)
Streptococcal Infection	0		0		1	(10)	1	(4)	2	(2)
Upper Respiratory Tract Infection	1	(5)	0		0		0		1	(1)
Urinary Tract Infection	0		1	(5)	0		0		1	(1)
Viraemia	0		0		1	(10)	0		1	(1)
Vulval Cellulitis	1	(5)	0		0		0		1	(1)
Injury, Poisoning And Procedural Complications	4	(18)	2	(10)	1	(10)	5	(18)	12	(15)
Allergic Transfusion Reaction	0		0		0		2	(7)	2	(2)
Excoriation	1	(5)	0		1	(10)	0		2	(2)
Fall	1	(5)	0		0		0		1	(1)
Injury	1	(5)	0		0		0		1	(1)
Post Procedural Haemorrhage	1	(5)	0		0		0		1	(1)

Table 7 Summary of Treatment-Emergent Adverse Events, All Treated Subject

Protocol No. P03579

	Number (%) of Subjects									
System Organ Class	Dose Group 1 (12 mg/kg/ day BID) /Age Group 1 (2 yrs-<7 yrs)		Dose Group 1 (12 mg/kg/ day BID) /Age Group 2 (7 yrs-<18 yrs)		Dose Group 2 (18 mg/kg/ day BID) /Age Group 1 (2 yrs-<7 yrs)		Dose Group 2 (18 mg/kg/ day BID) /Age Group 2 (7 yrs-<18 yrs)		Total	
Preferred Term	n=22		n=21		n=10		n=28		n=81	
Procedural Pain	1	(5)	0		0		1	(4)	2	(2)
Radiation Skin Injury	0		1	(5)	0		0		1	(1)
Transfusion Reaction	1	(5)	1	(5)	0		2	(7)	4	(5)
Investigations	7	(32)	8	(38)	3	(30)	11	(39)	29	(36)
Activated Partial Thromboplastin Time Prolonged	0		0		0		1	(4)	1	(1)
Alanine Aminotransferase Increased	2	(9)	1	(5)	0		3	(11)	6	(7)
Aspartate Aminotransferase Increased	1	(5)	1	(5)	0		1	(4)	3	(4)
Aspiration Bone Marrow	0		0		0		1	(4)	1	(1)
Blood Albumin Decreased	0		0		0		1	(4)	1	(1)
Blood Creatinine Increased	1	(5)	0		0		1	(4)	2	(2)
Blood Culture Positive	0		1	(5)	0		0		1	(1)
Blood Glucose Increased	0		0		1	(10)	0		1	(1)
Blood Phosphorus Decreased	1	(5)	0		0		0		1	(1)
Blood Urea Increased	0		0		0		1	(4)	1	(1)
Blood Urine Present	0		1	(5)	0		0		1	(1)
Breath Sounds Abnormal	1	(5)	0		0		0		1	(1)
C-Reactive Protein Increased	0		1	(5)	0		0		1	(1)
Chemotherapeutic Drug Level Increased	1	(5)	0		0		0		1	(1)
Electrocardiogram QT Prolonged	0		0		0		1	(4)	1	(1)

Table 7 Summary of Treatment-Emergent Adverse Events, All Treated Subject

Protocol No. P03579

	Number (%) of Subjects									
System Organ Class	Dose Group 1 (12 mg/kg/ day BID) /Age Group 1 (2 yrs-<7 yrs)		Dose Group 1 (12 mg/kg/ day BID) /Age Group 2 (7 yrs-<18 yrs)		Dose Group 2 (18 mg/kg/ day BID) /Age Group 1 (2 yrs-<7 yrs)		Dose Group 2 (18 mg/kg/ day BID) /Age Group 2 (7 yrs-<18 yrs)		Total	
Preferred Term	n=22		n=21		n=10		n=28		n=81	
Fibrin D Dimer Increased	0		0		0		1	(4)	1	(1)
Gamma-Glutamyltransferase Increased	0		0		0		1	(4)	1	(1)
Liver Function Test Abnormal	0		0		0		1	(4)	1	(1)
Neutrophil Count Decreased	0		0		1	(10)	1	(4)	2	(2)
Occult Blood Positive	0		1	(5)	0		1	(4)	2	(2)
Oxygen Saturation Decreased	0		1	(5)	0		0		1	(1)
Platelet Count Decreased	0		0		2	(20)	1	(4)	3	(4)
Protein Total Decreased	0		1	(5)	0		0		1	(1)
Staphylococcus Test	0		0		0		1	(4)	1	(1)
Transaminases Increased	2	(9)	1	(5)	0		0		3	(4)
Weight Decreased	0		0		0		1	(4)	1	(1)
White Blood Cell Count Decreased	0		0		1	(10)	0		1	(1)
Metabolism And Nutrition Disorders	4	(18)	7	(33)	0		6	(21)	17	(21)
Decreased Appetite	1	(5)	0		0		1	(4)	2	(2)
Dehydration	0		1	(5)	0		0		1	(1)
Fluid Retention	1	(5)	3	(14)	0		1	(4)	5	(6)
Hyperglycaemia	0		0		0		1	(4)	1	(1)
Hyperkalaemia	0		1	(5)	0		1	(4)	2	(2)
Hypernatraemia	0		1	(5)	0		0		1	(1)

Table 7 Summary of Treatment-Emergent Adverse Events, All Treated Subject

Protocol No. P03579

	Number (%) of Subjects									
System Organ Class	Dose Group 1 (12 mg/kg/ day BID) /Age Group 1 (2 yrs-<7 yrs)		Dose Group 1 (12 mg/kg/ day BID) /Age Group 2 (7 yrs-<18 yrs)		Dose Group 2 (18 mg/kg/ day BID) /Age Group 1 (2 yrs-<7 yrs)		Dose Group 2 (18 mg/kg/ day BID) /Age Group 2 (7 yrs-<18 yrs)		Total	
Preferred Term	n=22		n=21		n=10		n=28		n=81	
Hyperphosphataemia	0		0		0		1	(4)	1	(1)
Hypertriglyceridaemia	0		0		0		1	(4)	1	(1)
Hypocalcaemia	0		1	(5)	0		1	(4)	2	(2)
Hypokalaemia	2	(9)	3	(14)	0		1	(4)	6	(7)
Hypomagnesaemia	1	(5)	4	(19)	0		1	(4)	6	(7)
Hyponatraemia	0		1	(5)	0		0		1	(1)
Hypophagia	0		0		0		1	(4)	1	(1)
Hypophosphataemia	2	(9)	2	(10)	0		1	(4)	5	(6)
Musculoskeletal And Connective Tissue Disorders	2	(9)	2	(10)	1	(10)	3	(11)	8	(10)
Arthralgia	0		0		0		1	(4)	1	(1)
Back Pain	1	(5)	2	(10)	1	(10)	1	(4)	5	(6)
Bone Pain	1	(5)	0		0		1	(4)	2	(2)
Myalgia	0		1	(5)	0		0		1	(1)
Nervous System Disorders	1	(5)	5	(24)	0		9	(32)	15	(19)
Convulsion	0		1	(5)	0		0		1	(1)
Dizziness	0		2	(10)	0		1	(4)	3	(4)
Headache	1	(5)	2	(10)	0		8	(29)	11	(14)
Restless Legs Syndrome	0		0		0		1	(4)	1	(1)
Psychiatric Disorders	2	(9)	0		0		1	(4)	3	(4)

Table 7 Summary of Treatment-Emergent Adverse Events, All Treated Subject

Protocol No. P03579

System Organ Class<br>Preferred Term	Number (%) of Subjects<br>Dose Group 1 (12 mg/kg/ day BID) /Age Group 1 (2 yrs-<7 yrs)<br>n=22	Dose Group 1 (12 mg/kg/ day BID) /Age Group 2 (7 yrs-<18 yrs)<br>n=21	Dose Group 2 (18 mg/kg/ day BID) /Age Group 1 (2 yrs-<7 yrs)<br>n=10	Dose Group 2 (18 mg/kg/ day BID) /Age Group 2 (7 yrs-<18 yrs)<br>n=28	Total<br>n=81
Agitation	1 (5)	0	0	0	1 (1)
Delirium	1 (5)	0	0	0	1 (1)
Depressed Mood	0	0	0	1 (4)	1 (1)
Hallucination	1 (5)	0	0	0	1 (1)
Renal And Urinary Disorders	1 (5)	3 (14)	1 (10)	3 (11)	8 (10)
Chromaturia	0	0	0	1 (4)	1 (1)
Dysuria	0	1 (5)	0	0	1 (1)
Proteinuria	1 (5)	2 (10)	0	0	3 (4)
Renal Failure	0	0	1 (10)	0	1 (1)
Renal Failure Acute	1 (5)	0	0	2 (7)	3 (4)
Reproductive System And Breast Disorders	2 (9)	0	0	0	2 (2)
Pruritus Genital	2 (9)	0	0	0	2 (2)
Respiratory, Thoracic And Mediastinal Disorders	4 (18)	5 (24)	4 (40)	6 (21)	19 (23)
Acute Respiratory Distress Syndrome	0	0	0	1 (4)	1 (1)
Atelectasis	0	1 (5)	0	0	1 (1)
Cough	2 (9)	3 (14)	1 (10)	1 (4)	7 (9)
Dysphonia	0	0	0	1 (4)	1 (1)
Dyspnoea	0	1 (5)	0	0	1 (1)
Dyspnoea Exertional	0	0	0	1 (4)	1 (1)

Table 7 Summary of Treatment-Emergent Adverse Events, All Treated Subject

Protocol No. P03579

System Organ Class<br>Preferred Term	Number (%) of Subjects									
	Dose Group 1 (12 mg/kg/ day BID) /Age Group 1 (2 yrs-<7 yrs)<br>n=22		Dose Group 1 (12 mg/kg/ day BID) /Age Group 2 (7 yrs-<18 yrs)<br>n=21		Dose Group 2 (18 mg/kg/ day BID) /Age Group 1 (2 yrs-<7 yrs)<br>n=10		Dose Group 2 (18 mg/kg/ day BID) /Age Group 2 (7 yrs-<18 yrs)<br>n=28		Total<br>n=81	
Epistaxis	1	(5)	1	(5)	0		2	(7)	4	(5)
Hypoxia	1	(5)	0		0		0		1	(1)
Nasal Congestion	0		0		1	(10)	0		1	(1)
Nasal Dryness	0		0		0		1	(4)	1	(1)
Oropharyngeal Pain	1	(5)	0		1	(10)	2	(7)	4	(5)
Painful Respiration	0		1	(5)	0		0		1	(1)
Pharyngeal Erythema	0		0		0		1	(4)	1	(1)
Pleural Effusion	0		1	(5)	1	(10)	0		2	(2)
Rales	0		1	(5)	0		0		1	(1)
Respiratory Failure	0		0		1	(10)	0		1	(1)
Rhinorrhoea	0		1	(5)	0		0		1	(1)
Tachypnoea	0		0		0		2	(7)	2	(2)
Skin And Subcutaneous Tissue Disorders	9	(41)	8	(38)	2	(20)	8	(29)	27	(33)
Alopecia	0		0		1	(10)	0		1	(1)
Dermatitis	1	(5)	0		0		1	(4)	2	(2)
Erythema	2	(9)	0		0		0		2	(2)
Exfoliative Rash	0		0		0		1	(4)	1	(1)
Nail Bed Inflammation	1	(5)	0		0		0		1	(1)
Pain Of Skin	1	(5)	0		0		0		1	(1)

Table 7 Summary of Treatment-Emergent Adverse Events, All Treated Subject

Protocol No. P03579

	Number (%) of Subjects									
	Dose Group 1 (12 mg/kg/ day BID) /Age Group 1 (2 yrs-<7 yrs)		Dose Group 1 (12 mg/kg/ day BID) /Age Group 2 (7 yrs-<18 yrs)		Dose Group 2 (18 mg/kg/ day BID) /Age Group 1 (2 yrs-<7 yrs)		Dose Group 2 (18 mg/kg/ day BID) /Age Group 2 (7 yrs-<18 yrs)		Total	
System Organ Class<br>Preferred Term	n=22		n=21		n=10		n=28		n=81	
Palmar-Plantar Erythrodysaesthesia Syndrome	0		1	(5)	0		0		1	(1)
Papule	0		1	(5)	0		0		1	(1)
Petechiae	2	(9)	2	(10)	0		0		4	(5)
Pruritus	3	(14)	5	(24)	0		3	(11)	11	(14)
Rash	4	(18)	1	(5)	1	(10)	4	(14)	10	(12)
Rash Erythematous	0		1	(5)	0		0		1	(1)
Rash Macular	0		0		0		1	(4)	1	(1)
Red Man Syndrome	0		0		0		1	(4)	1	(1)
Skin Irritation	0		0		0		1	(4)	1	(1)
Skin Mass	0		1	(5)	0		0		1	(1)
Urticaria	0		0		0		1	(4)	1	(1)
Vascular Disorders	6	(27)	4	(19)	0		8	(29)	18	(22)
Haematoma	1	(5)	1	(5)	0		1	(4)	3	(4)
Hypertension	4	(18)	1	(5)	0		3	(11)	8	(10)
Hypotension	1	(5)	3	(14)	0		4	(14)	8	(10)

AE=Adverse Event; BID= twice a day; yrs=years of age.

Source Data: P03579 data on file. Kenilworth (NJ): Merck Sharp & Dohme Corp.; 2013 Mar.

Based on available safety data (through 25-Jan-2013), a summary of treatment-related TEAEs organized by MedDRA SOC is provided for P03579 in Table 8 . The most frequently (>10% of subjects) reported treatment-related TEAEs were vomiting and nausea.

Table 8 Summary of Treatment-Related Treatment-Emergent Adverse Events, All Treated Subject

Protocol No. P03579

	Number (%) of Subjects				
System Organ Class	Dose Group 1 (12 mg/kg/ day BID) /Age Group 1 (2 yrs-<7 yrs)	Dose Group 1 (12 mg/kg/ day BID) /Age Group 2 (7 yrs-<18 yrs)	Dose Group 2 (18 mg/kg/ day BID) /Age Group 1 (2 yrs-<7 yrs)	Dose Group 2 (18 mg/kg/ day BID) /Age Group 2 (7 yrs-<18 yrs)	Total
Preferred Term	n=22	n=21	n=10	n=28	n=81
Subjects Reporting Any Adverse Event	11 (50)	5 (24)	3 (30)	16 (57)	35 (43)
Blood And Lymphatic System Disorders	1 (5)	0	0	4 (14)	5 (6)
Coagulopathy	0	0	0	2 (7)	2 (2)
Febrile Neutropenia	0	0	0	1 (4)	1 (1)
Thrombocytopenia	1 (5)	0	0	1 (4)	2 (2)
Cardiac Disorders	0	1 (5)	0	0	1 (1)
Bradycardia	0	1 (5)	0	0	1 (1)
Eye Disorders	1 (5)	0	0	0	1 (1)
Eye Pruritus	1 (5)	0	0	0	1 (1)
Gastrointestinal Disorders	8 (36)	3 (14)	2 (20)	9 (32)	22 (27)
Abdominal Pain	1 (5)	2 (10)	0	1 (4)	4 (5)
Abdominal Pain Upper	2 (9)	0	0	0	2 (2)
Diarrhoea	1 (5)	0	0	2 (7)	3 (4)
Flatulence	0	0	1 (10)	0	1 (1)

Table 8 Summary of Treatment-Related Treatment-Emergent Adverse Events, All Treated Subject

Protocol No. P03579

	Number (%) of Subjects									
System Organ Class	Dose Group 1 (12 mg/kg/ day BID) /Age Group 1 (2 yrs-<7 yrs)		Dose Group 1 (12 mg/kg/ day BID) /Age Group 2 (7 yrs-<18 yrs)		Dose Group 2 (18 mg/kg/ day BID) /Age Group 1 (2 yrs-<7 yrs)		Dose Group 2 (18 mg/kg/ day BID) /Age Group 2 (7 yrs-<18 yrs)		Total	
Preferred Term	n=22		n=21		n=10		n=28		n=81	
Gastrooesophageal Reflux Disease	0		0		0		1	(4)	1	(1)
Nausea	3	(14)	1	(5)	0		7	(25)	11	(14)
Stomatitis	1	(5)	0		2	(20)	1	(4)	4	(5)
Vomiting	6	(27)	1	(5)	0		7	(25)	14	(17)
General Disorders And Administration Site Conditions	1	(5)	0		0		2	(7)	3	(4)
Fatigue	0		0		0		2	(7)	2	(2)
Oedema Peripheral	1	(5)	0		0		0		1	(1)
Infections And Infestations	1	(5)	0		0		0		1	(1)
Rhinitis	1	(5)	0		0		0		1	(1)
Injury, Poisoning And Procedural Complications	1	(5)	0		0		0		1	(1)
Post Procedural Haemorrhage	1	(5)	0		0		0		1	(1)
Investigations	5	(23)	2	(10)	0		5	(18)	12	(15)
Alanine Aminotransferase Increased	2	(9)	1	(5)	0		2	(7)	5	(6)
Aspartate Aminotransferase Increased	1	(5)	1	(5)	0		1	(4)	3	(4)
Blood Phosphorus Decreased	1	(5)	0		0		0		1	(1)
Chemotherapeutic Drug Level Increased	1	(5)	0		0		0		1	(1)
Electrocardiogram QT Prolonged	0		0		0		1	(4)	1	(1)
Gamma-Glutamyltransferase Increased	0		0		0		1	(4)	1	(1)
Liver Function Test Abnormal	0		0		0		1	(4)	1	(1)

Table 8 Summary of Treatment-Related Treatment-Emergent Adverse Events, All Treated Subject

Protocol No. P03579

System Organ Class<br>Preferred Term	Number (%) of Subjects									
	Dose Group 1 (12 mg/kg/ day BID) /Age Group 1 (2 yrs-<7 yrs)		Dose Group 1 (12 mg/kg/ day BID) /Age Group 2 (7 yrs-<18 yrs)		Dose Group 2 (18 mg/kg/ day BID) /Age Group 1 (2 yrs-<7 yrs)		Dose Group 2 (18 mg/kg/ day BID) /Age Group 2 (7 yrs-<18 yrs)		Total	
	n=22		n=21		n=10		n=28		n=81	
Transaminases Increased	2	(9)	1	(5)	0		0		3	(4)
Metabolism And Nutrition Disorders	1	(5)	0		0		0		1	(1)
Decreased Appetite	1	(5)	0		0		0		1	(1)
Hypokalaemia	1	(5)	0		0		0		1	(1)
Hypophosphataemia	1	(5)	0		0		0		1	(1)
Nervous System Disorders	0		0		0		3	(11)	3	(4)
Headache	0		0		0		3	(11)	3	(4)
Reproductive System And Breast Disorders	1	(5)	0		0		0		1	(1)
Pruritus Genital	1	(5)	0		0		0		1	(1)
Respiratory, Thoracic And Mediastinal Disorders	1	(5)	0		0		0		1	(1)
Cough	1	(5)	0		0		0		1	(1)
Skin And Subcutaneous Tissue Disorders	1	(5)	0		1	(10)	1	(4)	3	(4)
Rash	1	(5)	0		1	(10)	1	(4)	3	(4)

AE=Adverse Event; BID= twice a day; yrs=years of age.

Source Data: P03579 data on file. Kenilworth (NJ): Merck Sharp & Dohme Corp.; 2013 Mar.
